# Accessing unexplored regions of sequence space in directed enzyme evolution via insertion/deletion mutagenesis

**DOI:** 10.1038/s41467-020-17061-3

**Published:** 2020-07-10

**Authors:** Stephane Emond, Maya Petek, Emily J. Kay, Brennen Heames, Sean R. A. Devenish, Nobuhiko Tokuriki, Florian Hollfelder

**Affiliations:** 1 Department of Biochemistry, University of Cambridge, Cambridge, CB2 1GA UK; 2 Michael Smith Laboratories, University of British Columbia, Vancouver, BC V6T 1Z4 Canada; 3 Present Address: Evonetix Ltd, Coldhams Business Park, Norman Way, Cambridge, CB1 3LH UK; 4 Present Address: Cancer Research UK Beatson Institute, Glasgow, G61 1BD UK; 5 Present Address: Institute for Evolution and Biodiversity, Westfälische Wilhelms-Universität, Hüfferstrasse 1, 48149 Münster, Germany; 6 Present Address: Fluidic Analytics, The Paddocks Business Centre, Cherry Hinton Road, Cambridge, CB1 8DH UK

**Keywords:** Proteins, Protein design, Synthetic biology

## Abstract

Insertions and deletions (InDels) are frequently observed in natural protein evolution, yet their potential remains untapped in laboratory evolution. Here we introduce a transposon-based mutagenesis approach (TRIAD) to generate libraries of random variants with short in-frame InDels, and screen TRIAD libraries to evolve a promiscuous arylesterase activity in a phosphotriesterase. The evolution exhibits features that differ from previous point mutagenesis campaigns: while the average activity of TRIAD variants is more compromised, a larger proportion has successfully adapted for the activity. Different functional profiles emerge: (i) both strong and weak trade-off between activities are observed; (ii) trade-off is more severe (20- to 35-fold increased *k*_cat_/*K*_M_ in arylesterase with 60-400-fold decreases in phosphotriesterase activity) and (iii) improvements are present in *k*_cat_ rather than just in K_M_, suggesting adaptive solutions. These distinct features make TRIAD an alternative to widely used point mutagenesis, accessing functional innovations and traversing unexplored fitness landscape regions.

## Abstract (web summary)

Insertions/Deletions (InDels) remain an untapped source of protein diversity in laboratory evolution. The method TRIAD generates libraries of random variants with short in-frame InDels using transposons, allowing a comparison of their evolutionary potential with widely-used point mutant libraries.

## Introduction

Directed evolution aims at identifying proteins with new functional traits by mimicking the natural process of genetic variation through mutations, followed by selection of improved variants. A major challenge for the success of this approach remains that only a very small fraction of the theoretically possible sequence space is accessible experimentally during any screening or selection process, so the type of library determines the success of directed evolution and the features of the functional proteins arising from such protein engineering. Expanding the diversity and the quality of gene libraries has been a major research focus to increase the chances of identifying new variants with desired functions. So far, most directed evolution (and, more generally, protein engineering) experiments have been performed using point substitutions for gene diversification, while insertions or deletions (InDels) remain an overlooked source of variation despite their frequent and functionally beneficial occurrence in natural protein evolution^1^. Combinatorial approaches to incorporate InDels at predefined positions, based on phylogenetic and/or structural analyses, have been developed to alter catalytic specificities of enzymes^2–4^ or to improve the binding affinities of engineered antibodies^5,6^. While several methods for incorporating InDels randomly within a gene of interest have been developed, they show many limitations in terms of library quality and diversity. Most of these approaches generate frame-shifting InDels at high frequency (>66%) (e.g., using error-prone DNA polymerases^7,8^, terminal deoxynucleotidyl transferase^9^, exonucleases^10,11^, tandem duplication insertion^12^ or truncation^13^) and result in libraries that mostly consist of non-functional variants, which must be removed by high-throughput selection or screening. Methods based on the use of engineered transposons are designed to avoid frameshifts but so far have been limited to the generation of deletions^14,15^ or insertions of fixed length and defined sequences^16^.

In the present work, a strategy for random introduction of single short InDels of one, two or three nucleotide triplets (±3, 6 or 9 bp, which maintain the overall protein reading frame and generate both between-codon and cross-codon mutations) into a given DNA sequence (dubbed TRIAD: Transposition-based Random Insertion And Deletion mutagenesis) was established and validated by generating libraries of InDel variants of *Brevundimonas diminuta* phosphotriesterase (*wt*PTE), a highly efficient enzyme hydrolyzing the pesticide paraoxon^17^ with promiscuous esterase and lactonase activities^18^. The resulting TRIAD libraries were used to investigate the fitness effects of short InDels on *wt*PTE and compare it to that of substitutions. Moreover, screening these libraries for improved arylesterase activity revealed several hits that would have been inaccessible using traditional and widely used point substitution mutagenesis approaches, demonstrating that the introduction of InDels can harvest functional diversity in previously unexplored regions of protein sequence space.

## Results

### A strategy for creation of random InDel libraries

**Fig. 1:**
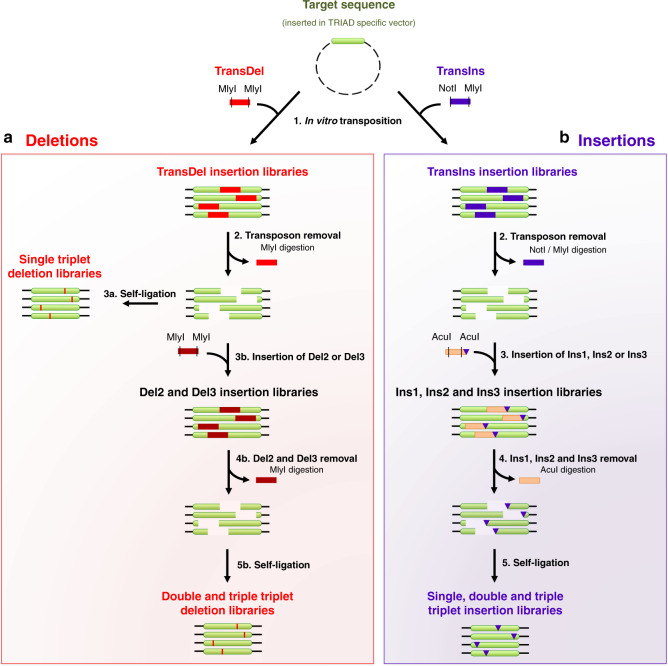
Schematic outline of TRIAD. **a** Generation of deletion libraries. Step 1: The TransDel insertion library is generated by in vitro transposition of the engineered transposon TransDel into the target sequence on circular plasmid DNA. Step 2: MlyI digestion removes TransDel together with 3 bp of the original target sequence and generate a single break per variant. Step 3a: self-ligation results in the reformation of the target sequence minus 3 bp, yielding a library of single variants with a deletion of one triplet^14^. Step 3b: DNA cassettes dubbed Del2 and Del3 are then inserted between the break in the target sequence to generate Del2 and Del3 insertion libraries. Step 4b: MlyI digestion removes Del2 and Del3 together with 3 and 6 additional bp of the original target sequence, respectively. Step 5b: self-ligation results in the reformation of the target sequence minus 6 and 9 bp, yielding libraries of single variants with a deletion of 2 and 3 triplets, respectively. Deletions are indicated by red vertical lines. **b** Generation of insertion libraries. Step 1: The TransIns insertion library is generated by in vitro transposition of the engineered transposon TransIns into the target sequence. Step 2: digestion by NotI and MlyI removes TransIns. Step 3: DNA cassettes dubbed Ins1, Ins3 and Ins3 (with, respectively, 1, 2 and 3 randomized NNN triplets at one of their extremities; indicated by purple triangles) are then inserted between the break in the target sequence to generate the corresponding Ins1, Ins2 and Ins3 insertion libraries. Step 4: *Acu*I digestion and 3′-end digestion by the Klenow fragment remove the cassettes, leaving the randomized triplet(s) in the original target sequence. Step 5: Self-ligation results in the reformation of the target sequence plus 3, 6 and 9 random bp, yielding libraries of single variants with an insertion of 1, 2 and 3 triplets, respectively.

**Fig. 2:**
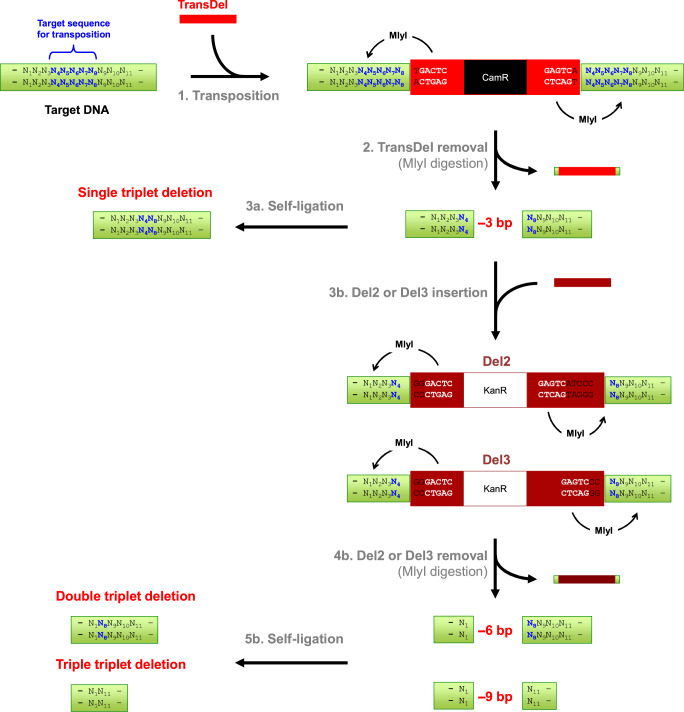
Mechanism for the generation of single, double and triple triplet nucleotide deletions. The target sequence is located on a plasmid with ampicillin resistance (bla) and neither the target sequence nor the plasmid contain any MlyI, NotI or AcuI restriction sites. Step 1. Two MlyI recognition sites (5′GAGTC(N)_5_↓) are positioned at each end of TransDel, 1 bp away from the site of transposon insertion. Transposition with TransDel results in the duplication of 5 bp (N_4_N_5_N_6_N_7_N_8_) of the target DNA at the insertion point. TransDel carries a selection marker (resistance gene against chloramphenicol; CamR) enabling the recovery of in vitro transposition products after transformation into *E. coli*. Step 2. MlyI digestion removes TransDel together with 8 bp of the target DNA (4 bp at each end), leaving blunt ends and resulting in the removal of a contiguous 3 bp sequence from the target DNA (N_5_N_6_N_7_). Step 3a. Self-ligation reforms the target DNA minus 3 bp, as previously described^14^. Step 3b. Alternatively, blunt-ended cassettes Del2 or Del3 are ligated into the gap left upon TransDel removal for the generation of 6 and 9 bp deletions, respectively. Both Del2 and Del3 also contain two MlyI recognition sites advantageously positioned towards the ends of the cassettes. These cassettes also contain a different marker than TransDel (resistance gene against kanamycin; KanR) to avoid cross-contamination. Step 4b. MlyI digestion removes Del2 and Del3 together with, respectively, 3 and 6 additional bp of the original target DNA. In the case of Del2, MlyI digestion results in the removal of a 3 bp sequence (N_2_N_3_N_4_) on one side of the cassette. In the case of Del3, MlyI digestion results in the removal of two 3 bp sequence (N_2_N_3_N_4_) on both side of the cassette (N_2_N_3_N_4_ and N_8_N_9_N_10_). Step 5b. Self-ligation reforms the target DNA minus 6 or 9 bp.

**Fig. 3:**
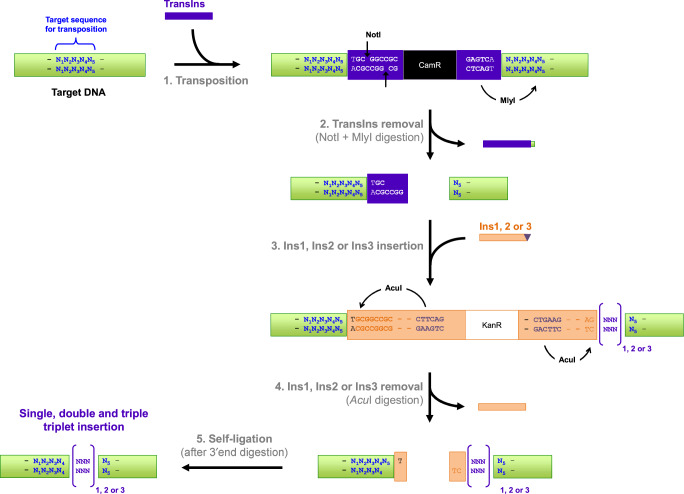
Mechanism for the generation of single, double and triple randomized triplet nucleotide insertions. Step 1. TransDel is an asymmetric transposon with MlyI at one end and NotI at the other end. Both recognition sites are positioned 1 bp away from TransIns insertion site. Upon transposition, 5 bp (N_1_N_2_N_3_N_4_N_5_) of the target DNA are duplicated at the insertion point of TransIns. Step 2. Double digestion with NotI and MlyI results in the removal of TransIns. Digestion with MlyI removes TransIns with 4 bp (N_1_N_2_N_3_N_4_) of the duplicated sequence at the transposon insertion site. Digestion with NotI leaves a 5′, 4-base cohesive overhang. Step 3. DNA cassettes Ins1, Ins2 and Ins3 (Ins1/2/3) carrying complementary ends are ligated in the NotI/MlyI digested TransIns insertion site. Ins1, Ins2 and Ins3 carry, respectively, 1, 2 and 3 randomized bp triplets at their blunt-ended extremities ([NNN]_1, 2 or 3_; indicated in purple). Ins1/2/3 contain two AcuI recognition sites (5′CTGAAG(16/14)) strategically positioned towards their ends. One site is located so that AcuI will cleave at the point where the target DNA joins Ins1/2/3. The other site is positioned so that AcuI will cut inside Ins1/2/3 to leave the randomized triplet(s) with the target DNA. Step 4. Digestion with AcuI removes Ins1/2/3 leaving 3′, 2-base overhangs with the target DNA (i.e., 5′N_5_T on one end and 5′TC on the end carrying the randomized triplet(s)). Digestion with the Large Klenow fragment generates blunt ends by removing the overhangs. This step also enables to discard the extra nucleotide (N_5_) from the sequence duplicated during the transposition. Step 5. Self-ligation reforms the target DNA with one, two or three randomized nucleotide triplets.

TRIAD consists of a single transposition reaction followed by successive cloning steps for the generation of deletions or insertions (Fig. 1; see also Supplementary Fig. S1 for a more detailed illustration). TRIAD’s first step is an in vitro Mu transposition reaction^19^ that ultimately determines the location of the forthcoming single InDel event in each variant. The reaction is performed using engineered mini-Mu transposons, dubbed TransDel and TransIns (Supplementary Fig. S2a), that are inserted randomly within the target DNA sequence during the first step of TRIAD, resulting in the generation of transposon insertion libraries. The ends of TransDel and TransIns were designed to bring about deletion and insertion libraries, respectively. TransDel is functionally equivalent to the previously described MuDel transposon^14^ with recognition sites for the type IIS restriction enzyme MlyI at both ends. The positioning of MlyI sites within TransDel enables the deletion of 3 bp at random positions within the target sequence upon MlyI digestion and self-ligation (Fig. 2), as previously described^14^. This strategy was extended to the generation of longer contiguous deletions (i.e., −6 and −9 bp) with a second stage, involving the insertion and subsequent MlyI-mediated removal of custom-made cassettes (dubbed Del2 and Del3; Figs. 1a and 2). For the generation of insertions, a transposon, TransIns, was designed as—in contrast to TransDel—an asymmetric transposon (Fig. 1b and Fig. 3), bearing different end sequences (NotI on one end and MlyI on the other). The latter site marks subsequent insertion sites for the ligation of custom-made shuttle cassettes: Ins1, Ins2 and Ins3 carrying one, two and three randomized nucleotide triplets, respectively. Further digestion using a type IIS restriction enzyme (AcuI) removes the shuttle sequence but leaves triplet insertions behind (Figs. 1b and 3).

### Generation of random InDel libraries by TRIAD

To validate TRIAD, we generated InDel libraries from the gene encoding a highly expressed variant of phosphotriesterase (*wt*PTE) that had been previously used as starting point to generate an efficient arylesterase by laboratory evolution^20,21^. Six independent libraries of *wt*PTE InDel variants were generated, comprising three deletion (−3, −6 and −9 bp) and three insertion libraries (+3, +6 and +9 bp). Without taking into account potential redundancy in the target DNA sequence, the maximal theoretical diversity of TRIAD libraries is a product of the number of positions (~1000 bp for *wt*PTE) and the diversity introduced at each position: one deletion of each length for deletion libraries and the diversity of randomized triplets (64^1^, 64^2^ and 64^3^ for one, two or three NNN triplets) for insertion libraries. Therefore, the maximal theoretical diversity for *wt*PTE is ~1000 variants in each deletion library, and 6.4 × 10^4^, ~4.1 × 10^6^ and ~2.6 × 10^8^ for +3, +6 and +9 bp insertion libraries (see Supplementary Fig. 5). However, depending on the sequence context, two or more neighbouring events may result in identical final DNA sequence, which reduces the accessible theoretical diversity. Theoretical diversities at the protein level are further reduced due to codon degeneracy and occurrence of stop codons as a result of certain InDels (Supplementary Note 1; Supplementary Fig S5b, c). Practically, the size of our libraries was limited by transformation efficiency, achieving >10^6^ variants upon transformation into *E. coli*. Therefore, all deletions as well as +3 bp insertions were oversampled such that the library diversity was maintained between transformations, while the diversity of sampled transposition sites was maintained in larger +6 and +9 bp insertion libraries, with only a fraction of theoretical library diversity generated from the outset.

### Quality assessment of TRIAD libraries

**Fig. 4:**
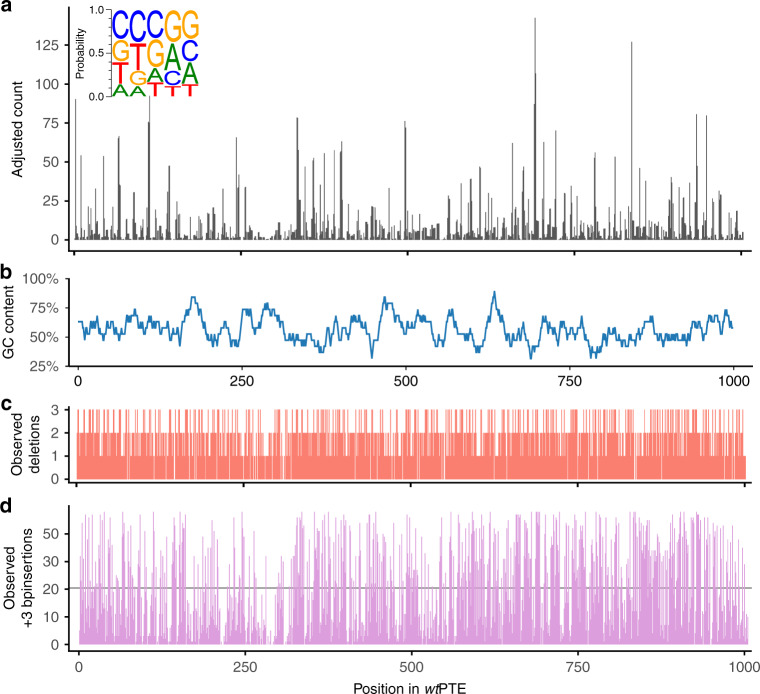
Mutagenesis efficiency of TRIAD. The composition of InDel libraries in the *wt*PTE gene was determined by deep sequencing and validated using Sanger sequences from randomly chosen variants. **a** Relative frequency of TransDel transposon insertion across *wt*PTE, derived from −3 bp deletions observed in deep sequencing and normalized for sequencing depth and InDel redundancy in DNA sequence (see Supplementary Methods 2.7). The relative transposon insertion site preference was determined by extracting the five-nucleotide target sequence around each detected −3 bp deletion (in forward and reverse complement direction, since the direction of transposon insertion is unknown). The frequency of insertion at each position was used to weigh the contribution to consensus sequence, then normalized to give the proportion of each nucleotide per position in the Mu transposon consensus sequence. **b** The GC content in the *wt*PTE gene, calculated as the moving average in a 19 bp window. **c** Distribution and number of detected distinct DNA deletions in −3, −6 and −9 bp libraries combined per *wt*PTE position. **d** Distribution and number of observed +3 bp mutations per DNA position in +3 bp library, compared with the median 20.85 variants per position (horizontal line). Due to varying InDel redundancy depending on sequence context, the theoretical DNA diversity per position is between 42 and 48 variants (see Supplementary Fig. S5). Analogous plots for +6 and +9 bp libraries are shown in Supplementary Fig. S8. Source data are available in the Source data file.

The quality of the TRIAD libraries was assessed with Sanger sequencing to obtain long read accurate information, as well as deep next-generation sequencing to quantify the library sizes, distribution and diversity of InDels over the target sequence, and the transposition bias. All 121 Sanger-sequenced variants displayed only a single modification from the initial transposon insertion, without any incidental mutations, and 90 among them showed anticipated in-frame InDel mutations (see Supplementary Note 2 for details). We then obtained a next-generation sequencing dataset containing ~1 × 10^6^ total 75-bp reads per deletion library and >3 × 10^6^ reads per insertion library (Supplementary Methods S3–S7; Supplementary Fig. S6 and Supplementary Table S4). In all libraries, the targeted in-frame InDels were found in high abundance, reaching more than 10^5^ variants detected by deep sequencing in the most diverse +6 and +9 bp libraries (>10^3^ unique deletions and >10^5^ unique insertions overall; Table 1). In agreement with Sanger sequencing of individual variants (Supplementary Table S1, Supplementary Note 2), frameshifts were rare in the −3 bp deletion library (4%) and more frequent (>20%) in the others (Supplementary Table S4b). Analysis of −3 and +3 bp libraries showed that TransDel and TransIns insertion accessed 85 and 95% of all possible DNA positions, respectively (Fig. 4c, d).

**Table 1:** Mutagenesis efficiency of TRIAD analysed by deep sequencing.

TRIAD library	Deletions			Insertions		
	−3 bp	−6 bp	−9 bp	+3 bp	+6 bp	+9 bp
Observed unique in-frame DNA InDels^a^	639	690	613	20,872	107,165	103,720
Proportion relative to theoretical DNA diversity (%)^b^	85%	92%	84%	45%	3.8%	<0.1%
Observed unique in-frame protein InDels^a^	530	562	492	8400	58,559	94,303
InDels with no adjacent amino acid substitution	302 (57%)	320 (58%)	307 (63%)	4671 (58%)	34008 (58%)	56086 (59%)
InDels with adjacent amino acid substitution^c^	223 (42%)	234 (42%)	180 (37%)	3359 (42%)	19561 (37%)	26691 (28%)
InDels resulting in truncated variants^c^	5	8	5	370	4990	11,526
Proportion relative to theoretical protein diversity (%)	90%	95%	89%	65%	24%	2%^d^

^a^Unique in-frame InDels (i.e., InDels of multiple of three nucleotides) were counted both at the DNA and the protein level.

^b^The proportion relative to the theoretical diversity accessible from the *wt*PTE sequence (both at the DNA and the protein level) was calculated as the ratio between the number of unique in-frame InDels observed by deep sequencing and the theoretical diversity for a given TRIAD library (see Supplementary Fig. S5).

^c^Adjacent amino acid substitutions and truncations (resulting from the occurrence of stop codons) arise from cross-codon transposon insertions and from insertions containing stop codons, reducing the number of observed unique protein InDels.

^d^The theoretical protein diversity of +9 bp library is estimated as 21× larger (20 amino acids and a stop codon) than the calculated diversity of +6 bp library.

Previous analysis of Mu transposon target site preference^22^ suggests a strong preference for pyrimidines in position 2 and purines in position 4 of the 5 bp transposition site, based on 806 observed transpositions. By contrast, we observed similar frequencies for most deletions, with 52% of all detected deletions having between 10 and 99 reads per variant, and only 11% of all deletions (Supplementary Table S6) occurring more frequently across all three libraries combined (200 reads or more per variant; see distribution in Supplementary Fig. S7). We extracted the weakly preferred transposition sequence to be 5′N-Py-G/C-Pu-N (see insert in Fig. 4a; Supplementary Table S5). We conclude that the sequence bias of Mu transposons is less pronounced than previously thought^22,23^ and does not clearly correlate with GC content (Fig. 4b).

Good coverage of possible positions in the insertion libraries translates into high diversity at most positions in *wt*PTE (Fig. 4d; Supplementary Fig. S8a): 10 or more distinct DNA insertions were observed between 66% (+3 bp) and 80% (+6 and +9 bp libraries) of positions; furthermore, 100 or more insertions were detected in 34% (+6 bp) and 31% (+9 bp) of positions (Supplementary Fig. S8b). While insertion libraries were sequenced with a higher loading onto the flow cell, this was still insufficient to fully capture the diversity in the +6 and +9 bp libraries (24% and 2% at the protein level, respectively; Table 1), where each variant was observed only once or twice (Supplementary Fig. S7), and so the true diversity may be higher. When the transposition event occurs across two codons, the resulting InDels may exhibit an adjacent amino acid substitution: on protein level, an average of 39% of the InDels observed in the deep sequencing dataset of *wt*PTE variants exhibited such substitutions (Table 1). No significant bias was observed in the nucleotide composition of the in-frame insertions (Supplementary Fig. S9), indicating that TRIAD generates diverse insertion variants.

Our quality assessment of the TRIAD libraries shows that—beyond a weak bias during transposon insertion—TRIAD libraries show excellent coverage of >85% of positions in the DNA sequence of *wt*PTE. These results provide evidence that the TRIAD approach leads to large and diverse libraries of InDel variants through a set of straightforward cloning procedures that spanned just over 5 days (Supplementary Fig. S1).

### Fitness effects between InDels and point substitutions

**Fig. 5:**
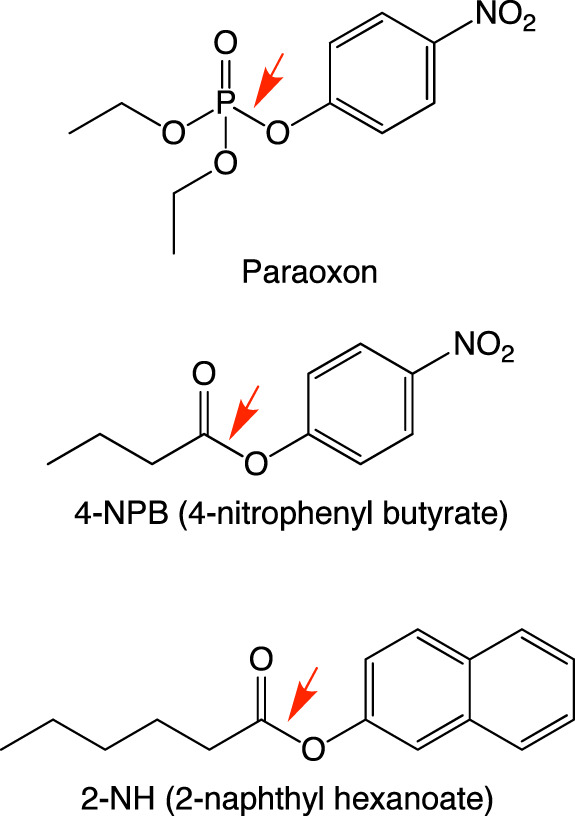
Structures of substrates. *wt*PTE catalyzes the hydrolysis of paraoxon (native substrate) and possess promiscuous activity against arylester substrates, e.g. 4-nitrophenyl butyrate and 2-naphthyl hexanoate.

**Fig. 6:**
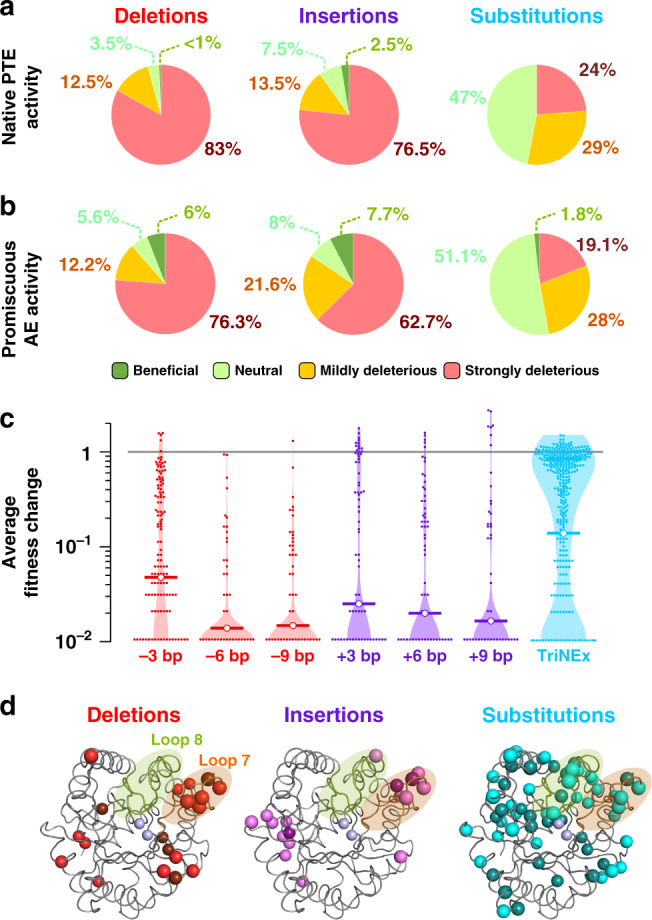
Fitness effects of InDels versus substitutions on *wt*PTE phosphotriesterase. **a** Distribution of fitness effects on native phosphotriesterase activity (paraoxon). **b** Distribution of fitness effects on promiscuous arylesterase activity (4-NPB). Fitness effects are classified as strongly deleterious (>10-fold activity decrease relative to *wt*PTE), mildly deleterious (10-fold to 1.5-fold decrease), neutral (<1.5-fold change), and beneficial (>1.5-fold increase). **c** The fitness change in relative phosphotriesterase activity by deletions, insertions and substitutions, measured as changes in initial rates as a consequence of mutations. The horizontal line indicates the geometric mean of the relative activities of the variants (see Supplementary Table S8). **d** Structural mapping of protein changes observed in variants retaining ≥50% of *wt*PTE activity level (PDB ID 4PCP). Grey spheres indicate the position of the catalytic Zn^2+^ ions, light spheres denote surface positions and dark spheres buried residues. Source data are available in the Source data file.

To compare the distribution of fitness effects of InDels vs. point substitutions, the levels of native phosphotriesterase (PTE; substrate: paraoxon; Fig. 5) and promiscuous arylesterase (AE; substrate: 4-nitrophenyl butyrate, 4-NPB; Fig. 5) activities were determined for several hundred *wt*PTE variants from each TRIAD library and from a trinucleotide substitution library (TriNEx library; Fig. 6; Supplementary Tables S8-9). Considering *wt*PTE is an evolutionarily “optimized” enzyme as a phosphotriesterase (based on the observation that it is operating near the diffusion limit for its native activity^17^), it is to be expected that very few mutations would be beneficial and that InDels are more deleterious than point substitutions overall. This expectation is underlined by the observation that 83% of deletions and 77% of insertions are strongly deleterious (<0.1 PTE activity), compared with only 24% in the substitution library (Fig. 6a). The average fitness change similarly favours substitutions and is an order of magnitude more deleterious for InDels (Fig. 6c).

However, of 485 deletions and 351 insertions assayed for PTE activity (Supplementary Tables S8-9), a total of 12 were beneficial (>1.5-fold PTE activity increase) against a background of already-high catalytic efficiency. By contrast, no beneficial substitutions were found amongst the 342 substitutions screened. Similar frequencies were observed with respect to deleterious fitness changes induced by InDels vs. point substitutions in *wt*PTE’s promiscuous arylesterase activity, with 76% of deletions and 62% of insertions strongly deleterious in comparison to only 19% of substitutions (Fig. 6b; Supplementary Table S8). The frequency of InDels beneficial for arylesterase activity was found to be at least 3-fold higher than that of beneficial substitutions (6% and 7.7% for deletions and insertions, respectively, vs. 1.8% for substitutions; Fig. 6b).

Mapping the observed mutations to the 3D structure of *wt*PTE provided insight into the location of adaptive InDels in comparison with point substitutions. While substitutions selected for ≥50% of *wt*PTE activity are found throughout the protein, the positions of InDels triggering similar functional effect appear more clustered in loops and on the surface (Fig. 6d). Analysis of surface-accessible solvent area (SASA) suggests that mutations affecting the buried residues are more detrimental than surface-exposed ones (Supplementary Fig. S10 and Supplementary Table S10). This observation holds for both InDels and substitutions. For substitutions, the correlation between SASA and fitness effects on activity is weak, while only ~20% of neutral or beneficial InDels affect buried residues (cf. ~40% of substitutions), readily explained by the larger impact of InDels on presumably optimised packing in the protein core.

To further examine the differential effects of InDels and substitutions on protein stability, changes in soluble expression and fitness (i.e., PTE activity in cell lysates) were systematically recorded for several single triplet InDel variants (from TRIAD libraries −3 and +3 bp) as well as substitution variants (from the TriNEx library) (Supplementary Note 3; Supplementary Fig. S11; Supplementary Tables S11-12). Overall, this analysis indicates that InDels are, on average, more detrimental to kinetic stability (related to folding kinetics during expression in the cell)^24^ than substitutions. Interestingly, some InDel variants were affected by significant activity loss while showing soluble expression levels similar to *wt*PTE, suggesting that the deleterious effects in these cases were caused by active site effects (resulting in lowered catalytic activity) rather than by global protein destabilisation. In these variants with similar solubility, the detrimental effect of InDels on activity was also exerted over longer average distances from the active site compared with substitutions (Supplementary Note 3; Supplementary Table S12). Taken together these data suggest that both the reduced stability of InDel variants as well as a compromised catalytic machinery lead to an, on average, more detrimental impact of InDels on enzyme fitness compared with point substitutions.

### Screening and identification of adaptive InDels in *wt*PTE

To demonstrate that TRIAD libraries allow access to functional innovation via adaptive InDels, all the libraries generated from the full-length *wt*PTE gene (six libraries in total: −3, −6, −9, +3, +6 and +9 bp) were subjected to two parallel screening campaigns to identify variants with enhanced arylesterase activity against either 4-nitrophenyl butyrate (4-NPB) or 2-naphthyl hexanoate (2-NH) (Fig. 5). Both screening campaigns consisted of a general two-step assay workflow. Upon transformation of the TRIAD libraries into *E. coli*, the resulting colonies (around 1 to 3 × 10^4^ per library) were first screened for either 1-naphthyl butyrate (prior to subsequent screening against 4-NPB in crude cell lysates) or 2-NH hydrolysis (using the FAST Red indicator that reacts with the released naphthol product). Colonies expressing an active variant (300–600 per library) were subsequently grown, lysed and tested for enzymatic activity (for either 4-NPB or 2-NH) in 96-well plates. Note that screening assays on colonies and in cell lysates were both performed after expression of *wt*PTE variants in the presence of overexpressed GroEL/ES chaperonin to buffer the destabilizing effects of adaptive mutations (Supplementary Fig. S11; Supplementary Tables S11-12), as described previously^25^.

**Fig. 7:**
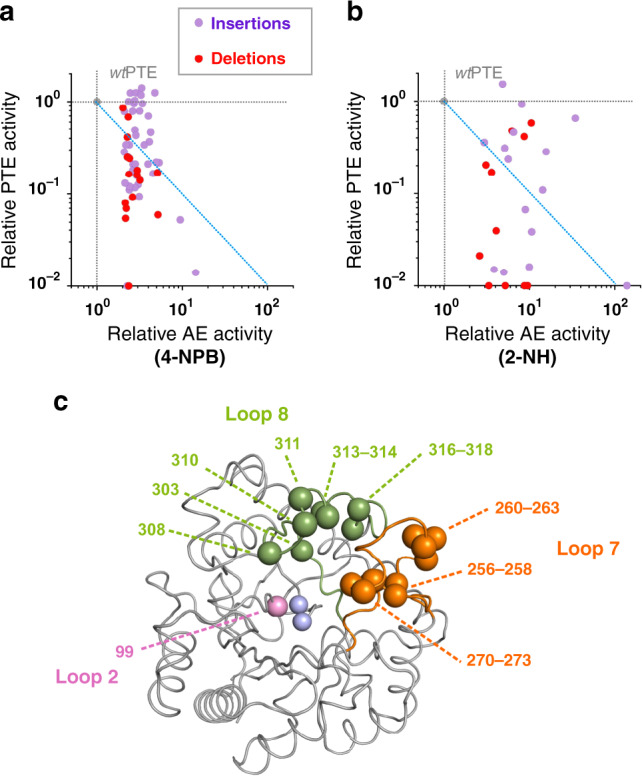
Identification of InDels improving the promiscuous arylesterase activity of *wt*PTE. **a, b** Changes in phosphotriesterase (native; PTE) and arylesterase (promiscuous; AE) activities among *wt*PTE. InDel variants identified upon screening against butyrate (4-NPB; panel **a**) and hexanoate (2-NH; panel **b**) esters, respectively. The enzymatic activities for each variant (shown as coloured dots) were measured in cell lysates and are plotted relative to those of *wt*PTE (grey dot). Data are averages of triplicate values. The dashed diagonal lines (in blue) demarcate the different trade-off regimes. Variants below the diagonal show a strong negative trade-off, with a large detriment to the original PTE activity (specificity ratio >100), as AE activity is improved. Above the diagonal variants with weak trade-off emerge as generalists (specificity ratio <100). **c** Position of the adaptive InDels in the PTE structure highlighting the frequency of mutations in loops 8 (green), 7 (orange) and 2 (pink). Grey spheres indicate the catalytic Zn^2+^ ions. Source data are available in the Source data file.

Overall, 81 hits (55 insertions and 26 deletions) were identified based on improved arylesterase activity against 2-NH or 4-NPB in cell lysates, with increases ranging from 2- to 140-fold in lysate activity compared with *wt*PTE (Table 2; Supplementary Table S13). In contrast to the adaptive substitutions previously identified^21^, these adaptive InDels appeared to have a more drastic effect on the native phosphotriesterase activity, indicating a more severe trade-off on average between maintaining original and enhancing promiscuous activity (average specificity ratio ~260; Supplementary Fig. S12). However, numerous individual mutants that do not show such strong negative trade-off were also identified (e.g., 64 variants out of 81 showed a specificity ratio <100; Fig. 7a, b; Supplementary Fig. S12).

**Table 2:** Analysis of InDel *wt*PTE variants with at least 2-fold improved arylesterase activity.

		Activity fold change relative to *wt*PTE^a^				Location of mutations^b^			
		Average effect	Median	Maximum	Minimum	Total	Loop 7	Loop 8	Other
InDels	Paraoxon	0.16	0.21	1.5	<0.01	81	58	15	8
	4-NPB	3.0	2.8	14.4	2.0	56	50	1	5
	2-NH	7.4	6.4	138.6	2.6	25	8	14	3
Deletions	Paraoxon	0.08	0.15	0.86	<0.01	26	17	4	5
	4-NPB	2.7	2.4	5.2	2.0	16	13	0	3
	2-NH	5.3	5.2	10.5	2.6	10	4	4	2
Insertions	Paraoxon	0.22	0.28	1.5	<0.01	55	41	4	10
	4-NPB	3.2	2.9	14.4	2.1	40	37	1	2
	2-NH	9.5	8.1	138.6	3	15	4	10	1

^a^Values refer to the activity change of all or AE positive variants relative to *wt*PTE obtained by comparing the initial rates *v*_0_ for the hydrolysis of paraoxon, 4-NPB or 2-NH to that of *wt*PTE at 200 μM substrate concentration, resulting in a dimensionless ratio. The average effect value was determined as the geometric mean of the relative activities of all the variants listed in Supplementary Table S13. The maximum, median and minimum changes correspond to the maximum, median and minimum relative activities for each substrate among the variants (see also Supplementary Fig. S13).

^b^The values refer to the number of insertions and/or deletions observed in the entire sequence of *wt*PTE or in specific regions (e.g., loop 7 (residues 252–278) and loop 8 (residues 299–313)).

Sequence analysis of the nature and the location of the InDels responsible for the improvement in arylesterase activity (Table 2) showed that all the adaptive InDels (apart from one double triplet nucleotide insertion, e.g., V99G/Q99aI99b) were clustered in two flexible regions of *wt*PTE, namely loop 7 (residues L252 to Q278) and loop 8 (residues S299 to P322) (Fig. 7c). Activity against 2-NH was improved by single InDels present in either loop while activity against 4-NPB was enhanced by InDels clustered in loop 7 (Table 2; Supplementary Fig. S13). Unexpectedly, the best variant (10.5-fold improvement in AE) found in the −9 bp deletion library exhibited a 12 bp deletion (presumably as a result of a rearrangement during the transposition step in the TRIAD process) resulting in a four-amino acid residue deletion (i.e., ΔA270-G273).

To further demonstrate that the identified InDels genuinely improve the arylesterase activity of *wt*PTE, the four variants exhibiting the strongest improvement against the 2-NH substrate (i.e., ΔA270-G273, P256R/G256aA256b, S256aG256b and G311a) were purified and characterized to give a 20- to 35-fold increased *k*_cat_*/K*_M_ for 2-NH, while decreasing paraoxon hydrolysis by around 100-fold (Fig. 7b; Table 3; Supplementary Fig. S15). While screening was performed in the presence of overexpressed chaperones, the kinetic and thermodynamic stability profiles of the identified hits were similar to that of the *wt*PTE parent (*T*_m_ > 75 °C and small deviations in GroEL/ES dependencies (relative to parent) between 0.9 and 1.3; Table 3; see Supplementary Figs. S14 and S16, Supplementary Tables S13-14).

**Table 3:** Properties of *wt*PTE InDel variants selected for improved arylesterase activity.

	AE			PTE			Thermal denaturation
PTE variant^a^	*k*_cat_ (s^−1^)	*K*_M_ (µM)	*k*_cat_/*K*_M_ (M^−1^ s^−1^)	*k*_cat_ (s^−1^)	*K*_M_ (µM)	*k*_cat_/*K*_M_ (M^−1^ s^−1^)	*T*_m_ (°C)^d^
*wt*PTE^b^	0.075 ± 0.004	179 ± 21	(4.2 ± 0.6) × 10^2^	1270 ± 27	57 ± 5	(2.2 ± 0.2) × 10^7^	78.1 ± 0.2
H254R^b^	0.27 ± 0.01	250 ± 32	(1.1 ± 0.2) × 10^3^	62 ± 3	7 ± 1	(8.9 ± 1.4) × 10^6^	88 ± 0.1
∆A270L271L272G273^c^	2.1 ± 0.1	258 ± 40	(8.2 ± 1.4) × 10^3^	55 ± 4	148 ± 22	(3.7 ± 0.7) × 10^5^	82 ± 1
P256R/G256aA256b^c^	4.6 ± 0.3	381 ± 59	(1.2 ± 0.3) × 10^4^	54 ± 3	988 ± 114	(5.4 ± 0.7) × 10^4^	84.3 ± 0.3
S256aG256b^c^	8.9 ± 0.7	821 ± 152	(1.1 ± 0.3) × 10^4^	13 ± 1	116 ± 21	(1.1 ± 0.3) × 10^5^	77.5 ± 0.4
G311a^c^	4.2 ± 0.2	292 ± 34	(1.5 ± 0.2) × 10^4^	39 ± 1	307 ± 25	(1.3 ± 0.2) × 10^5^	75.2 ± 0.3

AE, arylesterase (substrate: 2-NH); PTE, phosphotriesterase (substrate: paraoxon).

^a^The symbol ∆ before a residue (or a group of residues) signifies that this (or these) residue(s) have been deleted. Inserted residues are labelled using the number of the position after which they are inserted and alphabetical order (e.g., glutamine and tyrosine residues inserted in this order after the residues at position 230 would be labelled Q230aY230b).

^b^Kinetic parameters are from Tokuriki et al.^18^.

^c^See Supplementary Fig. S15 for detailed experimental conditions for Michaelis–Menten kinetics.

^d^Thermal denaturation for *wtPTE* and all InDel variants was measured with SYPRO Orange as the fluorescent probe and *T*_m_ is given as mean ± standard deviation (from six or more measurements; Supplementary Fig. S16). For variant H254R, the *T*_m_ value is from Wyganowki et al.^61^.

## Discussion

Point substitutions, small insertions and deletions account for most evolutionary changes among natural proteins^1^. The ratio of InDels to point substitutions covers a wide variety of ratios across different species, ranging from 1:5 in humans and primates^26^ to 1:20 in bacteria^27^, which indicates that InDels are typically subject to stronger purifying selection. In addition, protein sequence alignments have established that the majority of InDels fixed in protein-coding genes are short (i.e., encompassing 1–5 residues) and occur almost exclusively in loops linking secondary structure elements at the solvent-exposed surfaces of proteins^28–33^. While a large body of experimental evidence reports on the effects of substitutions, the impact of InDels on structural stability and functional divergence in protein evolution is still imperfectly understood, no doubt in part because convenient methods to introduce them in library experiments were missing. Substitutions, being merely side-chain alterations, tend to have local effects with typically minor consequences for the overall structure of a protein. By contrast, InDels alter the length of the backbone, opening the way to dramatically larger changes in the packing and orientation of domains that may result in more global effects on the protein structure^34–36^. Examples of InDels that cause significant repositioning of the backbone and nearby side chains to accommodate the extra or lost residues are on record^37–39^. If such rearrangements occur near the active site of a protein, the resulting structural changes can change specificity and activity^4,40,41^. In addition, short InDels occurring at oligomerisation interfaces have also been shown to have important effects on the stability and/or specificity of protein complexes^42,43^. A corollary of the comparatively drastic effect of InDels on protein structure is the perception that they are more deleterious. Indeed, this view is now experimentally corroborated by our work on *wt*PTE (Fig. 6) as well as a recent deep mutational scanning study investigating the fitness effects of single amino acid InDels on TEM-1 β-lactamase^44^. However, InDels have also been shown to be contribute to functional divergence in several enzyme families, such as lactate and malate dehydrogenases^45^, tRNA nucleotidyltransferases^46^, nitroreductases^47^, o-succinylbenzoate synthases^43^ and phosphotriesterase-like lactonases^4,48^.

An experimental platform that gives straightforward access to InDel libraries makes it possible to analyse the respective contributions of InDels and point substitutions as sources of functional innovation in experiments against the molecular fossil record. The reliability of gene randomization methods is essential for success in directed evolution experiments. Popular and practically useful methods must meet several key requirements: a high-yielding library generation protocol should create a large number of variants, avoid bias in gene composition or type of variant introduced, and be technically straightforward. When it comes to amino acid substitutions, several approaches (e.g. error-prone PCR, site-saturation mutagenesis starting with synthetic oligonucleotides) have been developed that partially or fully meet these criteria and are widely used. By contrast, the use of InDels in directed evolution experiments has been curtailed by practical limitations in existing methodologies to randomly incorporate insertions and/or deletions (see Supplementary Table S16). Consequently, their application in protein engineering has been sparse, with very few directed evolution campaigns on record that originate from such libraries. For example, the RID protocol^49^, the first attempt towards creating InDel libraries, relies on a complex protocol involving random cleavage of single stranded DNA, so that random substitutions are introduced unintentionally alongside the target mutations. Two other early methods, segmental mutagenesis^11^ and RAISE^9^, do not control for the length of the InDel and consequently produce libraries that primarily contain frameshifted variants. In contrast, a codon-based protocol dubbed COBARDE^50^ gives a pool of multiple codon-based deletions with <5% frameshifts but requires custom reprogramming of an oligonucleotide synthesizer to create mutagenic oligonucleotides. Alternatively, the viability of transposon-based protocols has been established for generating deletions of various sizes, up to gene truncation variants^13–15^. However, the only reported such protocol to create insertions, namely pentapeptide scanning mutagenesis^16,51^, merely gains access to insertions of defined size and sequence.

Improving on existing methodology, the TRIAD protocol meets all major requirements outlined above and gives easy access to large, diverse InDel libraries. The random insertion of a transposon gives excellent sampling of the entire target sequence (Fig. 4; Supplementary Fig. S8). Extensive sequencing shows that the Mu transposon is less biased than previously thought, so that functional effects upon insertion/deletion in any region of the protein can be taken advantage of. Library sizes upwards of 10^5^ variants were accessible by covering most of the theoretical diversity of up to two randomised amino acid insertions (Supplementary Fig. S5). Introduction of randomised larger insertions (+12 bp and beyond) is achievable^52^, but would lead to libraries that are larger than the typical screening capacity. Finally, the procedure is technically straightforward, consisting of transposition and cloning steps, and does not require access to specialized DNA synthesis equipment (as in ref. ^50^). The TRIAD workflow is a versatile process that can be adapted to create libraries focused on a specific region of a protein, applicable in cases where screening throughput is limited. This approach would be analogous to other procedures (although only a few^50,53^ have directly exemplified this case). In the case of TRIAD, this was typically achieved by adding an in-frame seamless cloning step using a type IIS restriction enzyme such as SapI (see Supplementary Note 4; Supplementary Fig. S17; Supplementary Table S15). InDel libraries constructed in this way showed good coverage of the target region, albeit with slightly more pronounced bias than whole-gene TRIAD, presumably due to increased sensitivity to preferential transposon insertions on a short target sequence. Alternatively, TRIAD can be further expanded with a recombination protocol (e.g., DNA shuffling or Staggered Extension Process) to generate variants combining multiple InDels, which can be screened in a high-throughput assay^54^. TransDel and TransIns transposons can be inserted at any point within a gene of interest, so the resulting InDels can affect adjacent codons in two-thirds of all possible transposon insertions and may lead to an adjacent point substitution. To generate InDels systematically located between codons, a possible strategy would be to couple transposon insertion with an intein-based codon-frame selection system^55^. This approach has been used previously to achieve codon substitution^56,57^ or deletion^15^ libraries, albeit at the cost of a high proportion of frameshifts and off-target variants (>60%). The TRIAD insertion libraries introduce (NNN)_n_ triplets at random positions within the target DNA sequence, which results in large theoretical library diversity (>10^8^ DNA variants in +9 bp library generated from the *wt*PTE gene; see SI). The sizes of insertion libraries could thus be reduced by advantageously combining such intein-based codon-frame selection system with the more restricted degenerate triplet insertions (e.g., using NNK or NNS).

The potential of InDel mutagenesis strategies in directed protein evolution is underlined by our comparative analysis of the fitness effect of InDels and point substitutions that showed InDels to be more likely to yield *wt*PTE variants with improved arylesterase activity than substitutions (Fig. 6b). A second point of comparison are the evolutionary trajectories followed starting with InDel vs point substitution libraries. The promiscuous esterase activity of *wt*PTE has previously been used as the starting point of a directed evolution effort that generated an arylesterase which hydrolysed 2-NH with high efficiency^21^. Here the mutation H254R, selected after the first round of mutagenesis, appeared to be a mutation on which the rest of the trajectory was highly contingent. InDel mutagenesis and selection puts us in a position to address the question whether alternative initial mutations would enable access to different evolutionary trajectories leading towards the same functional outcome. Based on the hypothesis that the use of a wider genetic and functional diversification (i.e., by both substitutions and InDels) might lead to a wider diversity of possible evolutionary trajectories, the first objective was to identify new adaptive mutations improving the promiscuous arylesterase activity of *wt*PTE by screening InDel libraries of *wt*PTE generated via TRIAD. This resulted in the identification of multiple beneficial deletions and insertions, confirming that introduction of InDels can give rise to functional and improved catalysts.

The functional potential of mutations can only be realized if the trade-off between mutational damage and functional innovation is not too severe^58,59^. Among the four hits characterized as purified enzymes, two show improved and two diminished thermal stability (<±6 °C in *T*_m_), suggesting that InDels can be beneficial or damaging, but both effects are small. This is no doubt due to the intrinsic robustness of the *wt*PTE structure that had been shown in previous evolution of this enzyme^59^. While *T*_m_, is a marker of thermodynamic stability (and usually a proxy for folding robustness), stability effects on evolution manifest themselves primarily in solubility for *wt*PTE^60^. We observe that a larger proportion of InDel library members fails the solubility criterion compared with substitution variants (Supplementary Fig. S11). However, the InDel variants identified in our work are selected for both stability and activity: a smaller proportion of an InDel library may fulfil these criteria, reflecting a similar dichotomy in evolution for catalysis (see below). Yet the survivors of this selective pressure seem to satisfy both challenges. This means that the outcomes of an InDel library screen are not especially (if at all) disadvantaged with regard to stability, but robust catalysts may emerge. Extrapolating ahead, it should be possible to combine adaptive InDels with additional mutations in future rounds of evolution, based on the robustness of InDel variants that suggests mutational tolerance and evolvability.

We further observed that four of these adaptive InDels increase arylesterase activity 20- to 35-fold (in *k*_cat_*/K*_M_) against 2-NH, which is more than the 2.6-fold difference brought about by the initial H254R mutation from the previous directed evolution^21^. For all four InDel variants, the improvement in 2-NH catalytic efficiency appears to be due to increased *k*_cat_ (from 28- to 120-fold), which outweighs an increased *K*_M_ in all four (from 2- to 6-fold). Similarly, all four variants increased in *K*_M_ for paraoxon (from 2 to 17-fold). On the other hand, the substitution H254R showed a different profile: it decreased *K*_M_ for paraoxon 8-fold, while hardly increasing it for 2-NH (1.4-fold)^21^. Therefore, the top InDel hits in the cell lysate screening are more disruptive for both the binding of paraoxon and arylester (2-NH) substrates than substitutions, as may be expected for mutations that alter the backbone structure, while remaining beneficial overall, by improving turnover (*k*_cat_ being related, at least in first approximation, to the chemical reaction step, given the small difference in expression^61^).

Despite the scarcity of facile random InDel mutagenesis methods until recently, several examples of an adaptive role of InDels in protein directed evolution have been observed. Work on TEM-1 β-lactamase using the original Mu transposon-based triplet deletion libraries identified variants with increased resistance towards the antibiotic ceftazidime, up to 64-fold in minimum inhibitory concentration^62^. A similar campaign that selected for eGFP variants with increased brightness in a colony screen identified the surprising eGFP-ΔGly4 deletion, which has significantly more cellular fluorescence likely due to increased refolding efficiency^63^. Finally, a recent focused library approach in a PTE-like lactonase with insertions into loop 7 (that is shorter in lactonases) led to variants enhanced in phosphotriesterase activity, with increased *k*_cat_ and decreased *K*_M_ for paraoxon (*k*_cat_/*K*_M_ increased up to 600-fold)^4^. Native lactonase activity was strongly affected in those variants with up to 10^4^-fold decreases in catalytic efficiency. These results in an enzyme closely related to PTE are very similar to our observations of the mixed effect of InDels on *wt*PTE, as explored based on the larger diversity of adaptive variants rendered available by TRIAD.

We conclude that evolutionary trajectories become accessible by screening InDel libraries obtained via TRIAD, establishing a paradigm that complements current strategies following the ‘one amino acid at the time’ adage^64^ which are believed to lead to successful outcomes slowly, yet steadily. The effect of InDels is on average more deleterious than substitutions (Fig. 6a), while the fraction of hits is increased in InDel libraries (Fig. 6b), suggesting that InDel library strategies tend to ‘polarize’ properties of library members towards extremes. For thermodynamically more difficult reactions than those studied here, this trend to more extreme outcomes may practically imply low hit rates, in which case high-throughput screening would become crucial. For example, ultrahigh-throughput screening based on droplet microfluidics^65,66^ could be combined with InDel mutagenesis to powerfully explore sequence space for evolutionary trajectories and individual variants that would not arise from epPCR mutagenesis libraries. It remains to be seen whether this way of ‘jumping’ (rather than ‘tiptoeing’) across sequence space yields functionally better catalysts—or just different ones.

## Methods

### Reagents

Paraoxon, 4-nitrophenyl butyrate (4-NPB), 1-naphthyl butyrate (1-NB), 2-naphthyl hexanoate (2-NH) and Fast Red were purchased from Sigma. FastDigest restriction endonucleases, MuA transposase and T4 DNA ligase were purchased from Thermo Fisher Scientific. DNA Polymerase I, Large (Klenow) Fragment, was purchased from New England Biolabs. All DNA modifying enzymes were used according to the manufacturer’s conditions. Oligonucleotides for PCR and adapter cloning experiments (Supplementary Table S17) were from Life Technologies and Sigma-Aldrich. The pGro7 plasmid for GroEL/ES overexpression was obtained from Takara Bio.

### Plasmid and transposon construction

To enable TRIAD, any recognition sequences for MlyI, NotI and AcuI in the target sequence or the plasmid containing the target sequence must be removed. A synthetic gene encoding *wt*PTE as well as dedicated cloning vectors were therefore designed and assembled prior to the construction of libraries. Detailed procedures and sequences can be found in the Supplementary Methods S1 and S2 for the design and construction of transposons (TransDel and TransIns), cloning cassettes (Del2, Del3, Ins1, Ins2 and Ins3) (Supplementary Methods S1 and Supplementary Fig. S2) and dedicated TRIAD vectors (pID-T7 and pID-Tet; Supplementary Methods S2 and Supplementary Fig. S4). The *wt*PTE gene lacking MlyI and AcuI sites (Supplementary Fig. S3) was synthesised by GenScript (NJ, USA). InDel libraries of *wt*PTE prepared in the pID-Tet vector were subcloned with NcoI and HindIII into pET-strep vector^25^ to express the strep-tag–PTE fusion protein for screening experiments and purification for the enzyme kinetics and stability assays.

### Generation of transposon insertion libraries

The generation of transposition insertion libraries with TransDel or TransIns was performed as previously described^67^. The transposons TransDel and TransIns (~1 kbp) were extracted from pUC57 by BglII digestion and recovered by gel electrophoresis and purification. Insertion of TransDel or TransIns in the pID-Tet plasmid (~2.7 kbp) containing *wt*PTE (~1 kbp) was performed using in vitro transposition using 300 ng of plasmid, 50 ng of transposon and 0.22 μg MuA transposase in a 20 μL reaction volume. After incubation for 2 h at 30 °C, the MuA transposase was heat-inactivated for 10 min at 75 °C. DNA products were purified and concentrated in 7 μL deionized water using a DNA clean concentrator kit (Zymo Research). Two microlitres of the purified DNA was used to transform *E. coli* E. cloni® 10G cells (>10^10^ CFU/µg pUC19; Lucigen) by electroporation. The transformants (typically 30,000–50,000 CFU) were selected on LB agar containing ampicillin (amp; 100 μg/mL) and chloramphenicol (CAM; 34 μg/mL). The resulting colonies were pooled, and their plasmid DNA extracted. The fraction of transformants with the transposon inserted into *wt*PTE (~27% of the entire plasmid length) corresponds to >8000 colonies, corresponding to >8-fold coverage of possible insertion sites (~1000) within *wt*PTE. The fragments corresponding to *wt*PTE containing the inserted transposon (~2 kbp) were obtained by double restriction digestion (NcoI/HindIII) followed by gel extraction and ligated in pID-Tet (50–100 ng). The ligation products were then transformed into electrocompetent *E. coli* E. cloni® 10 G cells. Upon selection on LB-agar-amp-cam, transformants (generally 1–2 × 10^6^ CFU) were pooled and their plasmid DNA extracted, yielding transposon (either TransDel or TransIns) insertion libraries. At this stage, transformation of these libraries into *E. coli* typically yielded >10^6^ CFU, maintaining oversampling of transposon insertion sites without skewing the distribution due to sampling.

### Generation of deletion and TriNEx variant libraries

TransDel insertion library plasmids were first digested with MlyI to remove TransDel. The fragments corresponding to linear pID-Tet-*wt*PTE plasmids (with a −3 bp deletion in *wt*PTE) were isolated by gel electrophoresis and purified. Self-circularization was then performed using T4 DNA ligase (Thermo Scientific) and 10–50 ng linearized plasmid (final concentration: ≥1 ng/μL). Upon purification and concentration, the ligation products were transformed into electrocompetent *E. coli* Ecloni® 10 G cells subsequently selected on LB-agar-amp, yielding a library of gene of interest variants with −3 bp random deletions^14^. For the construction of libraries of −6 and −9 bp deletion variants, cassettes Del2 and Del3 were extracted from pUC57 by SmaI digestion and recovered by gel electrophoresis and purification. For the construction of the TriNEx library, cassette SubsNNN was generated by PCR using pUC57-Del2 as template with primer pair Subs-F and Subs-B (Supplementary Table S17) and the resulting product (~1.1 kb) was recovered by gel extraction and electrophoresis. Cassettes Del2, Del3 and SubsNNN were then ligated into the MlyI linearized pID-Tet-*wt*PTE plasmid (50–100 ng) in a 1:3 molar ratio. After purification and concentration, the ligation products were transformed into electrocompetent *E. coli* Ecloni® 10 G. The transformants (generally 1–3 × 10^6^ colony forming units, CFU) were selected on LB agar containing ampicillin (100 µg/L) and kanamycin (Kan; 50 μg/mL). The plasmids (corresponding to Del2, Del3 and SubsNNN insertion libraries) were extracted from the colonies and subsequently digested using MlyI to remove the cassettes. The resulting linear pID-Tet-*wt*PTE products (containing the gene of interest with −6 or −9 bp deletions or 3 bp NNN substitutions) were recovered by gel electrophoresis, purified and subsequently self-circularized. The resulting products were transformed into electrocompetent *E. coli* Ecloni® 10G cells subsequently plated on LB-agar-amp, yielding libraries of *wt*PTE variants with −6 or −9 bp random deletions or triplet nucleotide substitutions^68^. All libraries were purified and stored in the form of plasmid solutions. Note that the TriNEx library of *wt*PTE used herein to compare the functional impact of InDels vs. point substitutions was also described in a previous reference^69^.

### Generation of insertion variant libraries

TransIns insertion library plasmids were digested with NotI and MlyI to remove TransIns. The linearized pID-Tet-*wt*PTE plasmids were recovered by gel electrophoresis and purification. Cassettes Ins1, Ins2 or Ins3 were extracted from pUC57 by NotI/MlyI digestion, recovered by gel electrophoresis and purification and inserted into the linearized pID-Tet-*wt*PTE plasmid (50–100 ng) in a 1:3 molar ratio. After purification and concentration, these ligation products were transformed into electrocompetent *E. coli* Ecloni® 10G and the transformants (generally 1 × 10^6^–3 × 10^6^ CFU) were selected on LB-agar-Amp-Kan. After extraction from the resulting colonies, the plasmids corresponding to Ins1, Ins2 and Ins3 insertion libraries were digested with AcuI. The linearized pID-Tet-*wt*PTE plasmids (with an insertion of 3, 6 and 9 bp in *wt*PTE) were recovered by gel electrophoresis, purified and subsequently treated with the Klenow fragment of DNA Polymerase I to remove 3′ overhangs created by AcuI digestion. After that blunting step, the plasmids were self-circularized. The resulting products were transformed into electrocompetent *E. coli* E. cloni® 10G cells subsequently plated on LB-agar-amp, yielding libraries of *wt*PTE variants with +3, +6 or +9 bp random insertions. All the libraries were purified and stored in the form of plasmid solutions.

### Sequencing and quality analysis

The mutagenesis efficiency of TRIAD was analysed both by Sanger sequencing (Supplementary Tables S1–S3) and deep sequencing. For the sequencing of individual *wt*PTE InDel variants obtained upon the transformation of libraries into *E. coli* (see above), individual colonies (~20 per library; Supplementary Tables S1 and S2) were randomly picked for plasmid extraction and subsequent Sanger sequencing. For deep sequencing, libraries were digested from pID-Tet with FastDigest restriction enzymes Bpu1102I and Van91I to give a pool of 1.3 kb linear fragments, which were processed using Nextera DNA Library Preparation Kit according to manufacturer’s instructions and sequenced on Illumina MiSeq using 2 × 75 bp paired-end sequencing. The reads were de-multiplexed, adaptors trimmed and assembled using PEAR^70^. Assembled and unassembled reads were mapped to the reference using Bowtie2^71^ and re-aligned to reference using the Needleman–Wunsch algorithm with gap open penalty 15 and gap extend penalty 0.5^72^. Placing InDels in particular sequence contexts may be inherently ambiguous because of potential InDel redundancy: when two or more InDels inserted at different positions in the target gene result in identical final sequence, no algorithm will be able to distinguish between them and the resulting InDel is always assigned to a single arbitrarily chosen original insertion or deletion site (see the discussion of examples in the Supplementary Methods S7). No attempt was made to correct for such ambiguity at this point. Resulting alignments were used to count the number of reads in which the mutations occur, their type and position using in-house developed Python scripts (see Supplementary Methods S4 and S5). To analyse the sequence preference for TransDel transposition, the counts were corrected for codon ambiguity by dividing the observed count equally between all positions where the deletion could have originated.

### Screening procedures for *wt*PTE variant libraries

Prior to screening, InDel variant libraries of *wt*PTE were excised by NcoI/HindIII double digestion and subcloned into pET-Strep vector. The resulting DNA libraries were transformed into *E. coli* BL21 (DE3) for experiments related to the analysis of fitness and soluble protein expression effects. For screening experiments to identify variants with improved arylesterase activity, the libraries were transformed in *E. coli* BL21(DE3) containing pGro7 for overexpression of the GroEL/ES chaperone system. Transformed cells (typically 2000–10,000 CFU) were plated on LB containing ampicillin (100 μg/mL) and chloramphenicol (34 μg/mL; if pGro7 was present). For fitness analysis experiments with PTE and AE the resulting transforming colonies were picked for screening in 96-well liquid format. When screening for improved arylesterase activity, the transformants were first subjected to an in situ colony screening for arylesterase activity prior to screening in 96-well liquid format.

For colony screening, the transformants were replicated using a filter paper (BioTrace NT Pure Nitrocellulose Transfer Membrane 0.2 μm, PALL Life Sciences), which was placed onto a second plate containing IPTG (1 mM), ZnCl_2_ (200 μM) and arabinose (0.2% (w/v)) for chaperone overexpression. After overnight expression at room temperature, the filter paper was placed into an empty Petri dish and cells were lysed prior to the activity assay by alternating three times between storage at −20 and 37 °C. Subsequently, top agar (0.5% agar in 100 mM Tris-HCl pH 7.5) containing either 1-NB or 2-NH (200 μM) and FAST Red (200 μM) was layered and a red precipitate (resulting from the complex formation between Fast Red and the naphthol product) developed within ~30 min. Colonies expressing an active PTE variant were then picked for further screening in 96-well liquid format.

For screening in 96-well liquid format, colonies (subjected to pre-screen or not) were transferred in 96-deep well plates containing 200 μL LB per well (with 100 μg/mL ampicillin; for experiments with pGro7: 34 μg/mL chloramphenicol) and re-grown overnight at 30 °C. Subsequently, 25 μL of the resulting cultures were used to inoculate 425 μL LB (containing the appropriate antibiotics) in 96-deep well plates. In the case of cells containing pGro7, the media was supplemented with arabinose or glucose (0.2% (w/v)) for overexpression or repression of GroEL/ES, respectively. After growth for 2–3 h at 30 °C, expression of PTE variants was induced by adding IPTG (1 mM final concentration) and cultures were incubated for an additional 2 h at 30 °C. Cells were then pelleted by centrifugation at 4 °C at maximum speed (3320 × *g*) for 5–10 min and the supernatant removed. Pellets were frozen overnight at −80 °C and, after thawing, lysed in 200 μL 50 mM Tris-HCl pH 7.5 supplemented with 0.1% (w/v) Triton-X100, 200 μM ZnCl_2_, 100 μg/mL lysozyme and 0.8 U/mL benzonase (Novagen). After 30 min of lysis, cell debris were spun down at 4 °C at 3320 × *g* for 20 min. Enzyme assays were performed in 96-well plates containing a volume of 200 μL per well (20 μL lysate, 180 μL of 200 μM substrate in 50 mM Tris-HCl, pH 7.5 supplemented with Triton-X100 (0.02% in the case of paraoxon and 0.1% in the case of 2-NH/FR)). For paraoxonase screening the lysate was pre-diluted 1:1000. The hydrolysis of paraoxon and 4-NPB were monitored by absorbance readings at 405 nm. The complex formation between 2-Naphthol and Fast Red was monitored at 500 nm.

### Purification of Strep-tagged PTE variants

pET-Strep-PTE plasmids were transformed into *E. coli* BL21 (DE3) grown for 8 h at 30 °C in Overnight Express Instant TB medium (Novagen) containing 100 µg/mL ampicillin and 200 μM ZnCl_2_ before lowering the temperature to 16 °C and continuing incubation overnight. Cells were harvested by centrifugation, resuspended and lysed using a 1:1 mixture of B-PER Protein Extraction Reagent (Thermo Scientific): 50 mM Tris-HCl buffer, pH 7.5 containing 200 μM ZnCl_2_, 100 μg/mL lysozyme and ~1 μL of benzonase per 100 mL. Cell debris was removed by centrifugation and the clarified lysate passed through a 45 μm filter before loading onto a Strep-Tactin Superflow High capacity column (1 mL). Strep-PTE variants were eluted with Elution buffer (100 mM Tris-HCl, pH 7.5, 200 μM ZnCl_2_ and 2.5 mM desthiobiotin) according to the manufacturer’s instructions (IBA Lifesciences).

### Kinetic characterization of PTE variants

Initial velocities (*V*_0_) were determined using purified enzyme at a range of substrate concentrations (0–200 μM or 0–2000 µM, depending on substrate and variant; see Supplementary Fig. S15) measured in triplicate in Tris-HCl (100 mM, pH 7.5) and ZnCl_2_ (200 µM). Reaction rates were monitored by following the complex formation between the product and Fast Red at 500 nm for 2-NH hydrolysis (in the presence of 2 mM Fast Red) and product formation at 405 nm for paraoxon hydrolysis. Enzyme concentration was adjusted depending on the assayed substrate and variant (see Supplementary Fig. S15). *K*_M_ and *k*_cat_ were determined by fitting the initial rates at each concentration to the Michaelis–Menten model using KaleidaGraph (Synergy Software).

### Thermal denaturation assay

Heat-induced unfolding of PTE variants was measured in triplicate over a range between 25 and 95 °C in a BioRad CFX Connect, using purified protein (5 and 10 µM final concentration) and SYPRO™ Orange Protein Gel Stain (5X and 10X final concentrations). Protein unfolding was monitored by measuring the change in fluorescence caused by binding of the dye (*λ*_excitation_ = 488 nm; *λ*_emission_, 500–750 nm) and the midpoint of denaturation (*T*_m_) was determined as the maximum of the first derivative for each temperature–fluorescence curve and averaged.

### Protein solubility assay

Protein solubility was analysed by SDS-PAGE. The amount of sample (soluble and/or insoluble fractions of different variants) to be loaded on the gel was determined by normalization to the OD_600_. The soluble fraction was assayed by analysing the clarified lysate by SDS-PAGE. To assay the insoluble fraction, the pellets obtained after lysis were resuspended in lysis buffer and analysed by SDS-PAGE. The intensity of the protein bands was measured using ImageJ.

### Reporting summary

Further information on research design is available in the Nature Research Reporting Summary linked to this article.

## Supplementary information

Reporting Summary

Supplementary Information

## Data Availability

Illumina raw sequencing reads were deposited with European Nucleotide Archive (https://www.ebi.ac.uk/ena) and are publicly available at accession number PRJEB28011. All other relevant data are available from the authors upon reasonable request. Source data are provided with this paper.

## Source data

Source Data

## Code availability

The source code along with instructions for all scripts involved in data processing are freely available at https://github.com/fhlab/TRIAD.

## References

[1] Chothia C, Gough J, Vogel C, Teichmann SA (2003). Evolution of the protein repertoire. Science.

[2] Park HS (2006). Design and evolution of new catalytic activity with an existing protein scaffold. Science.

[3] Herman A, Tawfik DS (2007). Incorporating Synthetic Oligonucleotides via Gene Reassembly (ISOR): a versatile tool for generating targeted libraries. Protein Eng. Des. Sel..

[4] Hoque MA (2017). Stepwise loop insertion strategy for active site remodeling to generate novel enzyme functions. ACS Chem. Biol..

[5] Lamminmaki U (1999). Expanding the conformational diversity by random insertions to CDRH2 results in improved anti-estradiol antibodies. J. Mol. Biol..

[6] Mou Y (2018). Engineering improved antiphosphotyrosine antibodies based on an immunoconvergent binding motif. J. Am. Chem. Soc..

[7] Emond S (2008). A novel random mutagenesis approach using human mutagenic DNA polymerases to generate enzyme variant libraries. Protein Eng. Des. Sel..

[8] Kashiwagi K, Isogai Y, Nishiguchi K, Shiba K (2006). Frame shuffling: a novel method for in vitro protein evolution. Protein Eng. Des. Sel..

[9] Fujii R, Kitaoka M, Hayashi K (2014). Random insertional-deletional strand exchange mutagenesis (RAISE): a simple method for generating random insertion and deletion mutations. Methods Mol. Biol..

[10] Hida K (2010). Sites in the AAV5 capsid tolerant to deletions and tandem duplications. Arch. Biochem Biophys..

[11] Pikkemaat MG, Janssen DB (2002). Generating segmental mutations in haloalkane dehalogenase: a novel part in the directed evolution toolbox. Nucleic Acids Res..

[12] Kipnis Y, Dellus-Gur E, Tawfik DS (2012). TRINS: a method for gene modification by randomized tandem repeat insertions. Protein Eng. Des. Sel..

[13] Morelli A, Cabezas Y, Mills LJ, Seelig B (2017). Extensive libraries of gene truncation variants generated by in vitro transposition. Nucleic Acids Res..

[14] Jones DD (2005). Triplet nucleotide removal at random positions in a target gene: the tolerance of TEM-1 beta-lactamase to an amino acid deletion. Nucleic Acids Res..

[15] Liu SS (2016). A facile and efficient transposon mutagenesis method for generation of multi-codon deletions in protein sequences. J. Biotechnol..

[16] Hallet B, Sherratt DJ, Hayes F (1997). Pentapeptide scanning mutagenesis: random insertion of a variable five amino acid cassette in a target protein. Nucleic Acids Res..

[17] Caldwell SR, Newcomb JR, Schlecht KA, Raushel FM (1991). Limits of diffusion in the hydrolysis of substrates by the phosphotriesterase from *Pseudomonas diminuta*. Biochemistry.

[18] Roodveldt C, Tawfik DS (2005). Shared promiscuous activities and evolutionary features in various members of the amidohydrolase superfamily. Biochemistry.

[19] Haapa S, Taira S, Heikkinen E, Savilahti H (1999). An efficient and accurate integration of mini-Mu transposons in vitro: a general methodology for functional genetic analysis and molecular biology applications. Nucleic Acids Res..

[20] Kaltenbach, M., Jackson, C. J., Campbell, E. C., Hollfelder, F. & Tokuriki, N. Reverse evolution leads to genotypic incompatibility despite functional and active site convergence. *Elife* **4**, e06492 (2015). 10.7554/eLife.06492 PMC4579389 26274563

[21] Tokuriki N (2012). Diminishing returns and tradeoffs constrain the laboratory optimization of an enzyme. Nat. Commun..

[22] Haapa-Paananen S, Rita H, Savilahti H (2002). DNA transposition of bacteriophage Mu. A quantitative analysis of target site selection in vitro. J. Biol. Chem..

[23] Mizuuchi M, Mizuuchi K (1993). Target site selection in transposition of phage Mu. Cold Spring Harb. Symp. Quant. Biol..

[24] Sanchez-Ruiz JM (2010). Protein kinetic stability. Biophys. Chem..

[25] Tokuriki N, Tawfik DS (2009). Chaperonin overexpression promotes genetic variation and enzyme evolution. Nature.

[26] Ng PC (2008). Genetic variation in an individual human exome. PLoS Genet..

[27] Chen JQ (2009). Variation in the ratio of nucleotide substitution and indel rates across genomes in mammals and bacteria. Mol. Biol. Evol..

[28] Ajawatanawong P, Baldauf SL (2013). Evolution of protein indels in plants, animals and fungi. BMC Evol. Biol..

[29] Benner SA, Cohen MA, Gonnet GH (1993). Empirical and structural models for insertions and deletions in the divergent evolution of proteins. J. Mol. Biol..

[30] Lin M (2017). Effects of short indels on protein structure and function in human genomes. Sci. Rep..

[31] Pascarella S, Argos P (1992). Analysis of insertions/deletions in protein structures. J. Mol. Biol..

[32] Taylor MS, Ponting CP, Copley RR (2004). Occurrence and consequences of coding sequence insertions and deletions in Mammalian genomes. Genome Res..

[33] Toth-Petroczy A, Tawfik DS (2013). Protein insertions and deletions enabled by neutral roaming in sequence space. Mol. Biol. Evol..

[34] Grishin NV (2001). Fold change in evolution of protein structures. J. Struct. Biol..

[35] Studer RA, Dessailly BH, Orengo CA (2013). Residue mutations and their impact on protein structure and function: detecting beneficial and pathogenic changes. Biochem J..

[36] Toth-Petroczy A, Tawfik DS (2014). Hopeful (protein InDel) monsters?. Structure.

[37] Heinz DW, Baase WA, Dahlquist FW, Matthews BW (1993). How amino-acid insertions are allowed in an alpha-helix of T4 lysozyme. Nature.

[38] O’Neil KT, Bach AC, DeGrado WF (2000). Structural consequences of an amino acid deletion in the B1 domain of protein G. Proteins.

[39] Stott KM, Yusof AM, Perham RN, Jones DD (2009). A surface loop directs conformational switching of a lipoyl domain between a folded and a novel misfolded structure. Structure.

[40] Shortle D, Sondek J (1995). The emerging role of insertions and deletions in protein engineering. Curr. Opin. Biotechnol..

[41] Tawfik DS (2006). Biochemistry. Loop Grafting Orig. Enzym. Species Sci..

[42] Hashimoto K, Panchenko AR (2010). Mechanisms of protein oligomerization, the critical role of insertions and deletions in maintaining different oligomeric states. Proc. Natl Acad. Sci. USA.

[43] Odokonyero D (2014). Loss of quaternary structure is associated with rapid sequence divergence in the OSBS family. Proc. Natl Acad. Sci. USA.

[44] Gonzalez CE, Roberts P, Ostermeier M (2019). Fitness effects of single amino acid insertions and deletions in TEM-1 beta-lactamase. J. Mol. Biol..

[45] Boucher, J. I., Jacobowitz, J. R., Beckett, B. C., Classen, S. & Theobald, D. L. An atomic-resolution view of neofunctionalization in the evolution of apicomplexan lactate dehydrogenases. *Elife* **3,** e02304 (2014). 10.7554/eLife.02304 PMC4109310 24966208

[46] Neuenfeldt A, Just A, Betat H, Morl M (2008). Evolution of tRNA nucleotidyltransferases: a small deletion generated CC-adding enzymes. Proc. Natl Acad. Sci. USA.

[47] Akiva E, Copp JN, Tokuriki N, Babbitt PC (2017). Evolutionary and molecular foundations of multiple contemporary functions of the nitroreductase superfamily. Proc. Natl Acad. Sci. USA.

[48] Afriat-Jurnou L, Jackson CJ, Tawfik DS (2012). Reconstructing a missing link in the evolution of a recently diverged phosphotriesterase by active-site loop remodeling. Biochemistry.

[49] Murakami H, Hohsaka T, Sisido M (2002). Random insertion and deletion of arbitrary number of bases for codon-based random mutation of DNAs. Nat. Biotechnol..

[50] Osuna J, Yanez J, Soberon X, Gaytan P (2004). Protein evolution by codon-based random deletions. Nucleic Acids Res..

[51] Hayes F, Hallet B (2000). Pentapeptide scanning mutagenesis: encouraging old proteins to execute unusual tricks. Trends Microbiol..

[52] Jones DD, Arpino JA, Baldwin AJ, Edmundson MC (2014). Transposon-based approaches for generating novel molecular diversity during directed evolution. Methods Mol. Biol..

[53] Tizei, P. A. G., Harris, E., Renders, M. & Pinheiro, V. B. InDel assembly: a novel framework for engineering protein loops through length and compositional variation. *bioRxiv*10.1101/127829 (2017). 10.1038/s41598-021-88708-4 PMC8080606 33911147

[54] Skamaki, K. et al. In vitro evolution of antibody affinity via insertional mutagenesis scanning of an entire antibody variable region. *bioRxiv*10.1101/2020.04.26.062786 (2020). 10.1073/pnas.2002954117 PMC7959553 33067389

[55] Gerth ML, Patrick WM, Lutz S (2004). A second-generation system for unbiased reading frame selection. Protein Eng. Des. Sel..

[56] Daggett KA, Layer M, Cropp TA (2009). A general method for scanning unnatural amino acid mutagenesis. ACS Chem. Biol..

[57] Liu J, Cropp TA (2012). A method for multi-codon scanning mutagenesis of proteins based on asymmetric transposons. Protein Eng. Des. Sel..

[58] Bloom JD, Labthavikul ST, Otey CR, Arnold FH (2006). Protein stability promotes evolvability. Proc. Natl Acad. Sci. USA.

[59] Tokuriki N, Tawfik DS (2009). Stability effects of mutations and protein evolvability. Curr. Opin. Struct. Biol..

[60] Wyganowski KT, Kaltenbach M, Tokuriki N (2013). GroEL/ES buffering and compensatory mutations promote protein evolution by stabilizing folding intermediates. J. Mol. Biol..

[61] Raushel FM, Holden HM (2000). Phosphotriesterase: an enzyme in search of its natural substrate. Adv. Enzymol. Relat. Areas Mol. Biol..

[62] Simm AM, Baldwin AJ, Busse K, Jones DD (2007). Investigating protein structural plasticity by surveying the consequence of an amino acid deletion from TEM-1 beta-lactamase. FEBS Lett..

[63] Arpino JA, Reddington SC, Halliwell LM, Rizkallah PJ, Jones DD (2014). Random single amino acid deletion sampling unveils structural tolerance and the benefits of helical registry shift on GFP folding and structure. Structure.

[64] Tracewell CA, Arnold FH (2009). Directed enzyme evolution: climbing fitness peaks one amino acid at a time. Curr. Opin. Chem. Biol..

[65] Colin PY, Zinchenko A, Hollfelder F (2015). Enzyme engineering in biomimetic compartments. Curr. Opin. Struct. Biol..

[66] Mair P, Gielen F, Hollfelder F (2017). Exploring sequence space in search of functional enzymes using microfluidic droplets. Curr. Opin. Chem. Biol..

[67] Baldwin AJ, Arpino JA, Edwards WR, Tippmann EM, Jones DD (2009). Expanded chemical diversity sampling through whole protein evolution. Mol. Biosyst..

[68] Baldwin AJ, Busse K, Simm AM, Jones DD (2008). Expanded molecular diversity generation during directed evolution by trinucleotide exchange (TriNEx). Nucleic Acids Res..

[69] Kaltenbach M, Emond S, Hollfelder F, Tokuriki N (2016). Functional trade-offs in promiscuous enzymes cannot be explained by intrinsic mutational robustness of the native activity. PLoS Genet..

[70] Zhang J, Kobert K, Flouri T, Stamatakis A (2014). PEAR: a fast and accurate Illumina Paired-End reAd mergeR. Bioinformatics.

[71] Langmead B, Salzberg SL (2012). Fast gapped-read alignment with Bowtie 2. Nat. Methods.

[72] Rice P, Longden I, Bleasby A (2000). EMBOSS: the European Molecular Biology Open Software Suite. Trends Genet..

