# Supplementary material for: Accessing unexplored regions of sequence space in directed enzyme evolution via insertion/deletion mutagenesis
Source: Nat Commun. 2020 Jul 10;11:3469. doi: 10.1038/s41467-020-17061-3 (PMC7351745; doi:10.1038/s41467-020-17061-3)
Supplement: Supplementary file 1 — Reporting Summary [file 41467_2020_17061_MOESM1_ESM.pdf]

nature research

Corresponding author(s): F Hollfelder

Last updated by author(s): Apr 4, 2020

Reporting Summary

Nature Research wishes to improve the reproducibility of the work that we publish. This form provides structure for consistency and transparency in reporting. For further information on Nature Research policies, see Authors & Referees and the Editorial Policy Checklist .

Statistics

For all statistical analyses, confirm that the following items are present in the figure legend, table legend, main text, or Methods section.

n/a	Confirmed	
[ ]	[x]	The exact sample size (*n*) for each experimental group/condition, given as a discrete number and unit of measurement
[ ]	[x]	A statement on whether measurements were taken from distinct samples or whether the same sample was measured repeatedly
[x]	[ ]	The statistical test(s) used AND whether they are one- or two-sided<br>*Only common tests should be described solely by name; describe more complex techniques in the Methods section.*
[x]	[ ]	A description of all covariates tested
[x]	[ ]	A description of any assumptions or corrections, such as tests of normality and adjustment for multiple comparisons
[ ]	[x]	A full description of the statistical parameters including central tendency (e.g. means) or other basic estimates (e.g. regression coefficient) AND variation (e.g. standard deviation) or associated estimates of uncertainty (e.g. confidence intervals)
[x]	[ ]	For null hypothesis testing, the test statistic (e.g. *F*, *t*, *r*) with confidence intervals, effect sizes, degrees of freedom and *P* value noted<br>*Give P values as exact values whenever suitable.*
[x]	[ ]	For Bayesian analysis, information on the choice of priors and Markov chain Monte Carlo settings
[x]	[ ]	For hierarchical and complex designs, identification of the appropriate level for tests and full reporting of outcomes
[x]	[ ]	Estimates of effect sizes (e.g. Cohen's *d*, Pearson's *r*), indicating how they were calculated

Our web collection on statistics for biologists contains articles on many of the points above.

Software and code

Policy information about availability of computer code

Data collection	Electrophoresis gel images were analysed with ImageJ version 2 software distributed in the Fiji package. The enzyme kinetics data were fitted to the Michaelis-Menten model with KaleidaGraph (Synergy Software).
Data analysis	The source code along with instructions for all scripts involved in data processing are freely available at https://github.com/fhlab/TRIAD. The scripts use Python version 3.5 or above (tested with 3.5, 3.6 and 3.7), PEAR read assembler v.0.9.20 under an academic licence, Bowtie2 v.2.3.4, EMBOSS v.6.6 and Samtools v.1.9.

For manuscripts utilizing custom algorithms or software that are central to the research but not yet described in published literature, software must be made available to editors/reviewers. We strongly encourage code deposition in a community repository (e.g. GitHub). See the Nature Research guidelines for submitting code & software for further information.

Data

Policy information about availability of data

All manuscripts must include a data availability statement. This statement should provide the following information, where applicable:

- Accession codes, unique identifiers, or web links for publicly available datasets
- A list of figures that have associated raw data
- A description of any restrictions on data availability

Illumina raw sequencing reads were deposited with European Nucleotide Archive (https://www.ebi.ac.uk/ena) and are publicly available at accession number PRJEB28011. Results underlying Figure 5 are listed in Supplementary Table S9 and data underlying Figure 6 are listed in Supplementary Table S13.

Field-specific reporting

Please select the one below that is the best fit for your research. If you are not sure, read the appropriate sections before making your selection.

- [x] Life sciences
- [ ] Behavioural & social sciences
- [ ] Ecological, evolutionary & environmental sciences

For a reference copy of the document with all sections, see nature.com/documents/nr-reporting-summary-flat.pdf

Life sciences study design

All studies must disclose on these points even when the disclosure is negative.

Sample size	A random selection of variants were screened for native phosphotriesterase activity (N=485 combined across all deletion libraries, N=351 across insertion libraries and N=342 in the TriNEx substitution library).
Data exclusions	During screening of random variants in the libraries, frame-shifted variants were excluded form the reported average impact of InDel variants. In the measurement of enzymatic activities, the technical replicates with obvious issues (e.g. scrambled signal) were excluded from the analysis.
Replication	Enzymatic activities were measured in triplicate, starting from the same bacterial culture glycerol stocks, unless stated otherwise in the manuscript. These technical replicates were analyzed separately and the final values averaged.<br>The measurement of thermal denaturation were performed in triplicate at each experimental conditions and all samples that generated a signal with a defined Tm were used (n=6 or more).
Randomization	The study described in this manuscript used assays to find InDel variants of PTE with improved catalytic activity towards alternative substrates, and focuses on characterization of such variants. Since we were looking for improved catalysts rather than testing the behavior of the starting point, randomization is not relevant to study design.
Blinding	The study focuses on exploration of sequence space accessed by InDels and as is common with directed evolution campaigns, blinding is not applicable.

Reporting for specific materials, systems and methods

We require information from authors about some types of materials, experimental systems and methods used in many studies. Here, indicate whether each material, system or method listed is relevant to your study. If you are not sure if a list item applies to your research, read the appropriate section before selecting a response.

Materials & experimental systems

n/a	Involved in the study
x	Antibodies
x	Eukaryotic cell lines
x	Palaeontology
x	Animals and other organisms
x	Human research participants
x	Clinical data

Methods

n/a	Involved in the study
x	ChIP-seq
x	Flow cytometry
x	MRI-based neuroimaging
