# Supplementary material for: Accessing unexplored regions of sequence space in directed enzyme evolution via insertion/deletion mutagenesis
Source: Nat Commun. 2020 Jul 10;11:3469. doi: 10.1038/s41467-020-17061-3 (PMC7351745; doi:10.1038/s41467-020-17061-3)
Supplement: Supplementary file 2 — Supplementary Information [file 41467_2020_17061_MOESM2_ESM.pdf]

Supplementary Information

Accessing unexplored regions of sequence space in directed enzyme evolution *via* insertion/deletion mutagenesis

Stephane Emond[1,*], Maya Petek[1], Emily Kay[1], Brennen Heames[1], Sean Devenish[1], Nobuhiko Tokuriki[2] and Florian Hollfelder[1,*]

[1] Department of Biochemistry, University of Cambridge, Cambridge CB2 1GA, UK

[2] Michael Smith Laboratories, University of British Columbia, Vancouver, BC V6T 1Z4, Canada

* To whom correspondence should be addressed. Tel: +44 (0)1223 766048 Email: fh111@cam.ac.uk. Correspondence may also be addressed to Stephane Emond. Email: emond.stephane@gmail.com

TABLE OF CONTENTS

SUPPLEMENTARY NOTES 5

Supplementary Note 1: Detailed consideration of theoretical diversity and InDel redundancy.. 5

Supplementary Note 2: Library quality assessment by Sanger sequencing......................... 5

Supplementary Note 3: Effects of InDels *vs.* point substitutions on soluble enzyme expression 6

Supplementary Note 4: Focused InDel libraries generated by TRIAD 7

SUPPLEMENTARY METHODS.. 9

Supplementary Method S1. Design, construction and preparation of transposons and cloning cassettes.. 9

Supplementary Method S2. Design and assembly of pID vectors....................................... 9

Supplementary Method S3. wtPTE reference sequence 10

Supplementary Method S4. NGS Step 1: Raw data processing 11

Supplementary Method S5. NGS Step 2: Parsing and statistics generation 12

Supplementary Method S6. Treatment of point mutations.. 13

Supplementary Method S7. Treatment of InDel redundancy... 13

Supplementary Method S8. Comparison of the effects of InDels *vs.* point substitutions on soluble expression and enzyme activity... 14

SUPPLEMENTARY FIGURES.. 15

Supplementary Figure S1. Schematic outline and timeline of the procedure for the generation of random InDel libraries 17

Supplementary Figure S2. Engineered transposons and cloning cassettes used in TRIAD. 20

Supplementary Figure S3: Sequence of the synthetic *wt*PTE gene and its corresponding protein product 21

Supplementary Figure S4: Vectors for the generation of InDel variant libraries................. 23

Supplementary Figure S5: Theoretical diversities of the InDel libraries obtained with TRIAD 25

Supplementary Figure S6: Sequencing coverage in the NGS of the TRIAD libraries of *wt*PTE. 28

Supplementary Figure S7: Distribution of observed number of reads per mutation. 30

Supplementary Figure S8: Number of distinct insertions observed per position in *wt*PTE. 33

Supplementary Figure S9: Distribution of nucleotide bases in the randomized inserts of the +3, +6 and +9 bp libraries 34

Supplementary Figure S10: Relationship between mutational tolerance and solvent-accessible surface area (SASA) in *wt*PTE 35

Supplementary Figure S11: Comparison of the effects on activity and solubility (*i.e.*, kinetic stability) between InDel (from TRIAD libraries -3 bp and +3 bp) and substitution variants (from TriNEx library) of wtPTE. 36

Supplementary Figure S12: Activity trade-offs in *wt*PTE InDel variants improved in arylesterase activity. 37

Supplementary Figure S13: Insertions and deletions improving the arylesterase activity of *wt*PTE by > 2-fold. 38

Supplementary Figure S14: Soluble and insoluble expression of PTE hits in the presence and absence of GroEL/ES chaperones. 39

Supplementary Figure S15: Kinetic characterization of InDel *wt*PTE variants with improved arylesterase activity. 41

Supplementary Figure S16: T_m measurement of *wt*PTE and four selected hits 44

Supplementary Figure S17: Application of TRIAD for the generation of focused InDel libraries. 46

SUPPLEMENTARY TABLES 48

Supplementary Table S1. Mutagenesis efficiency of TRIAD – individual variants 48

Supplementary Table S2. Sequence analysis of naïve InDel libraries of *wt*PTE obtained with TRIAD. 49

Supplementary Table S3. Frequency of in-frame InDels observed among randomly sequenced *wt*PTE variants (as recorded in Table S2). 53

Supplementary Table S4A: Deep sequencing coverage statistics. 56

Supplementary Table S4B: Proportion of frameshifts 57

Supplementary Table S5: TransDel consensus site preference 58

Supplementary Table S6: Statistics on number of reads supporting each variant 59

Supplementary Table S7. Number of reads per distinct deletion observed by deep sequencing in *wt*PTE deletion libraries generated *via* TRIAD. 60

Supplementary Table S8. Fitness effects in TRIAD (insertion and deletion) and trinucleotide substitution libraries of *wt*PTE. 61

Supplementary Table S9. Functional analysis of TRIAD and trinucleotide substitution libraries of *wt*PTE against paraoxon and 4-NPB 63

Supplementary Table S10. Analysis of solvent-accessible surface area of mutated residues in *wt*PTE variants retaining ≥50% of the parental paraoxonase activity. 64

Supplementary Table S10a. List of residues in *wt*PTE InDel variants retaining ≥50% of the parental paraoxonase activity. 64

Supplementary Table S10b. List of residues in wtPTE substitution variants retaining ≥50% of the parental paraoxonase activity. 65

Supplementary Table S11. Activity and solubility of InDel (-3 and +3 bp) and substitutions (TriNucleotide Exchange; TriNEx) variants of *wt*PTE phosphotriesterase. 67

Supplementary Table S12. Effects of InDels (-3 and +3 bp) and substitutions (TriNucleotide Exchange; TriNEx) on activity and soluble expression of *wt*PTE phosphotriesterase. 70

Supplementary Table S13. Cell lysate activity levels of InDel variants of *wt*PTE improved in arylesterase activity. 72

Supplementary Table S13A: Promiscuous activity against 4-NPB. 72

Supplementary Table S13B: Promiscuous activity against 2-NH. 74

Supplementary Table S14. Change in soluble expression of PTE InDel variants improved in arylesterase activity in the presence or absence of chaperone co-expression............... 76

Supplementary Table S15. Sequence analysis of naïve TRIAD libraries focused on Loop 7 of *wt*PTE. 77

Supplementary Table S16. Methods developed for the generation of libraries with random insertions, repeats and/or deletions. 82

Supplementary Table S17. Oligonucleotides used in this study. 85

SUPPLEMENTARY REFERENCES 86

SUPPLEMENTARY NOTES

Supplementary Note 1: Detailed consideration of theoretical diversity and InDel redundancy

The theoretical diversity (*i.e.*, the total number of possible variants) accessible *via* such modifications will depend both on the type of InDel that is introduced and on the target sequence (Supplementary Figure 5). For instance, as deletions can occur once at each position of the target DNA sequence, the maximal possible theoretical diversity of deletion libraries is identical to the number of nucleotides (Supplementary Figure S5A), *e.g.*, ~1,000 possible deletion variants for a ~1 kbp target sequence (*e.g.*, *wt*PTE). By contrast, since TRIAD inserts degenerate nucleotide triplets (*e.g.*, $[NNN]_{1,\ 2\ or\ 3}$ corresponding to 64 (=4^3), 4,096 (=4^6) and 262,144 (=4^9) possible sequences, respectively), the number of possible insertion variants will depend both on the length of the target sequence and the size of the insertions. The maximal possible theoretical diversity for insertion libraries generated from *wt*PTE is 6.4×10^4 (=64×10^3), ~4.1×10^6 (=$64^2\times10^3$), and ~2.6×10^8 (=$64^3\times10^3$), corresponding to +3, +6 and +9 bp insertions, respectively (Supplementary Figure S5A). Because of potential InDel redundancy depending on the target sequence (*i.e.*, two or more neighbouring InDels can result in the same DNA variant; Supplementary Figure S5B), the theoretical diversities accessible from a given DNA sequence are usually lower (see Supplementary Figure S5C in the specific case of *wt*PTE). Theoretical diversities at the protein level (*i.e.*, the number of protein variants that have the intended InDel length) are further reduced due to codon degeneracy and occurrence of stop codons as a result of certain InDels (Supplementary Figure S5C). Practically, the size of our libraries was limited by transformation efficiency, achieving > 10^6 variants upon transformation into *E. coli.* Therefore, deletions as well as +3 bp insertions were oversampled such that the library diversity was maintained between transformations, while the diversity of sampled transposition sites was maintained in larger +6 bp and +9 bp insertion libraries, with only a fraction of theoretical library diversity generated from the outset.

Supplementary Note 2: Library quality assessment by Sanger sequencing

In addition to the deep next-generation sequencing described in the main text, the accuracy of the TRIAD approach (specifically the number of intended in-frame InDels, unwanted frameshifts and incidental mutations) was also assessed using Sanger sequencing to give the reader a picture how such an 'everyday analysis' of a handful of individual *wt*PTE InDel variants would fare. To this end around 20 colonies from each naïve InDel library of individual *wt*PTE variants were randomly picked (after the final transformation step into *E. coli*) and 121 variants in total sequenced (Supplementary Tables S1-3). All the sequenced variants displayed only a single modification resulting from the initial transposon insertion and 90 among them (74%; corresponding to 89 unique InDels) showed anticipated in-frame InDel mutations (86% of the deletion variants; 61% of the insertion variants; Supplementary Tables S1-2). Most in-frame InDels were observed only once and were distributed throughout the

*wt*PTE sequence (Supplementary Table S3). No frameshift was observed among sequenced variants from the -3 bp library, which is generated without shuttle cloning steps in contrast to the other libraries. Frameshifts were more frequent among variants from the +3, +6 and +9 bp insertion libraries (~40% of the sequenced variants). Higher frameshift frequency in insertion libraries may be due to exonuclease over-digestion by the Klenow fragment of DNA polymerase I, which removes 3' overhangs left by AcuI digestion (Figure 2B). Note that no incidental additional base pair point mutations located elsewhere in the variants' sequence (*i.e.*, at positions different to that of the initial transposon insertion sites) and resulting from the TRIAD cloning process were detected. On the protein level, TRIAD may generate a secondary point substitution contiguous to the introduced InDel [1], depending on the point of insertion of TransDel or TransIns in the reading frame of the target sequence. This occurs when the InDel is not inserted at previous codon boundaries (statistically in two of three cases, although not all such events lead to amino acid substitution). As a result, 22% of the InDels observed in individual *wt*PTE variants exhibited such an adjacent substitution.

We conclude that the accuracy of the TRIAD procedure can be assessed based on a small number of sequences (n = 121, giving 89 unique in-frame InDels), to provide a quality control step informing in TRIAD library synthesis that is a representative measure of the distribution of InDels over the target sequence and assess the coverage afforded by the initial transposition step prior to diversification *via* InDel mutagenesis (Table 1), in lieu of a deep next-generation sequencing approach.

Supplementary Note 3: Effects of InDels *vs.* point substitutions on soluble enzyme expression

InDels are more detrimental to the fitness of *wt*PTE by one order of magnitude in comparison to point substitutions (see main text; Figure 5C). Enzyme fitness is reflective of both enzyme activity (i.e., catalytic efficiency) and the concentration of soluble and functional enzyme which itself relate to protein stability [2]. Thermodynamic stability (*i.e.*, the difference in free energy between the native and unfolded state *in vitro*) is often used to describe the relationship between protein stability and soluble and functional expression in the cell (*e.g.*, p53)[3]. However, in the case of *wt*PTE, the level of soluble and functional enzyme has previously been shown to correlate with kinetic stability (related to folding kinetics during expression in the cell) [4]. Therefore, to further investigate the stability effect of InDels *vs.* point substitutions, changes in expression levels of several InDel (TRIAD libraries: -3 bp deletions and +3 bp insertions) and point substitution (TriNEx library) variants were examined and correlated with fitness (native phosphotriesterase activity against paraoxon, measured in cell lysates) (Supplementary Figure S11; Supplementary Tables S11-12; see also supplementary methods). Overall, this analysis confirmed that InDels are more deleterious to soluble expression and protein stability than substitutions. Indeed, 17 out 30 deletions and 11 out of 27 insertions were found to be strongly destabilizing (> 2-fold decrease in soluble expression relative to *wt*PTE) while this was the case for only 6 out of 30 substitution variants (Supplementary Figure S11; Supplementary Tables S12). Likewise, the average impact on

soluble expression (mean solubility change) was up to 1.5-fold lower for InDels in comparison to substitutions (Supplementary Table S12). The stronger decrease in protein solubility observed in the case of InDels was also correlated to their more detrimental effect on fitness (enzyme activity) (Supplementary Figure S11; Supplementary Table S12).

Interestingly, some InDels affecting core residues that are relatively distant from the enzyme's catalytic centre were functionally deleterious (< 10-fold decrease in PTE activity) while retaining similar soluble expression levels (≤1.5-fold change) to the parent wtPTE (Supplementary Table S12). Indeed, while average distances to the catalytic metals for all solubility-neutral mutations were similar (16.4 ± 5.1, 17.3 ± 5 and 16.5 ± 5.6 ångströms for deletions, insertions and substitutions, respectively; Supplementary Table S12), non-destabilizing but functionally deleterious insertions and deletions were on average more distant to the active site than substitutions (13.9 ± 4.8 and 16.8 ± 5 ångströms for deletions and insertions, respectively versus 9.9 ± 3.3 ångströms for substitutions; Supplementary Table S12). This observation illustrates how InDels may trigger active site changes with functional effects at a longer range than substitutions.

Supplementary Note 4: Focused InDel libraries generated by TRIAD

TRIAD was additionally applied to focus the InDel mutagenesis on a specific targeted region within a protein by adding an in-frame seamless cloning step using a type IIS restriction enzyme such as SapI (strategy outlined in Supplementary Figure S17) [5]. This approach requires the target region to be extracted from its original gene and cloned in its own target plasmid with flanking SapI recognition sequences. This allows transposon integration into the target region in isolation from the rest of the gene. In parallel, an adapter plasmid is constructed, comprising the original gene in which the target region is replaced by an adapter sequence. This adapter sequence is designed with flanking SapI recognition sequences in order to allow the subcloning of the target region containing the randomly inserted transposon back inside its original gene. This last step results in the generation of the transposon insertion library focused on the region of interest, upon which the cloning steps of TRIAD leading to the generation of InDel libraries (Figure 1) can be performed.

To demonstrate this targeted approach of TRIAD, two deletion (-3 and -6 bp) and one insertion (+3 bp) libraries were generated in the sequence encoding *wt*PTE's active site loop 7 (L7), which has shown to be crucial for the specificity of *wt*PTE and homologous lactonases in previous rational InDel mutagenesis studies [6, 7]. In vitro transposition reactions were performed on a vector containing the L7 - encoding DNA sequence (from Leu252 to Gln278; 81 bp) flanked by SapI restriction sites (Supplementary Figure S13). After isolation by SapI digestion, the resulting L7 TransDel and TransIns insertion libraries were then subcloned into a plasmid containing a modified wtPTE gene with a SapI-adapter instead of L7. This additional cloning step enabled to recreate a full wtPTE gene with TransDel or TransIns randomly inserted - in theory - at all 81 positions within L7. After application of the further steps of TRIAD, libraries with insertions and deletions limited to L7 only were generated. Intermediate and final library transformation steps yielded diversities of >10^6 variants,

practically oversampling by >10^5-fold the theoretical diversity of the libraries (81 possible transposon insertion sites in L7). Sequence analysis of randomly chosen variants revealed the distribution of codons deleted in L7. Whilst there is good coverage of the target sequence (~70% of residues are deleted at least once), there is a bias of deletions toward certain residues, especially Leu262, Leu272 and the residues neighbouring them (Supplementary Table S15). These biases are likely caused by preferential transposon insertions at specific points along the DNA sequence encoding Loop 7.

SUPPLEMENTARY METHODS

Supplementary Method S1. Design, construction and preparation of transposons and cloning cassettes

DNA sequences corresponding to the TransDel transposon (Supplementary Figure S2A) and the Del2 cassette (previously dubbed Insertion Replacement Cassette in [8]) were synthesized and cloned into pUC57 (Supplementary Figure S2D) at the EcoRV site (GenScript, NJ, USA). Cloning strategies involving double stranded oligonucleotide adapters (Supplementary Table S14) were used to generate pUC57-TransIns (Supplementary Figure S2A) from pUC57-TransDel, and pUC57-Del3 (Supplementary Figure S2B), -Ins1, -Ins2 and -Ins3 (Supplementary Figure S2C) from pUC57-Del2. For all adapter cloning experiments, each pair of custom phosphorylated oligonucleotides (100 µM in 50mM Tris-HCl pH 8.0, 100 mM NaCl, 1mM EDTA) were mixed to a final concentration of 50 µM and annealed in a PCR thermocycler ((1) 2 min at 95°C, (2) 10 min at 52°C and (3) hold at 4°C). The resulting adapters were then ligated to a final concentration of 125 nM into their target plasmid (50-100 ng). The ligation products were then transformed into electrocompetent *E. coli* E. cloni® 10G cells. Plasmid pUC57-TransIns was generated by inserting the TransIns adapter in pUC57-TransDel at EcoRI/SpeI sites. Plasmid pUC57-Del3 was obtained by inserting the Del3 adapter in pUC57-Del2 at EcoRI/SpeI sites. Plasmids pUC57-Ins1, -Ins2 and -Ins3 correspond to libraries of inserts of one, two and three nucleotide triplets, respectively. First, an intermediate plasmid, dubbed pUC57-Ins, was obtained by inserting the Ins adapter in pUC57-Del2 at EcoRI/SpeI sites. Adapters Ins1, Ins2 and Ins3 were then inserted in pUC57-Ins at NcoI/HindIII sites to generate separate plasmid libraries corresponding to pUC57-Ins1, -Ins2 and -Ins3, respectively. In this last step, each DNA library was extracted from around 10^7 *E. coli* Ecloni® 10G transforming colonies.

Supplementary Method S2. Design and assembly of pID vectors

Two expression vectors, dubbed pID-T7 (expression under the control of T7 promoter) and pID-Tet (expression under the control of Tet promoter), were specifically designed for the generation of InDel libraries following the TRIAD approach. These vectors do not contain any MlyI, AcuI and NotI restriction sites in their sequence and were assembled from three different modules (for origin of replication, ampicillin resistance (AmpR) selection and expression/cloning) separated by three restriction sites, AflII, AatII and SpeI (Supplementary Figure S4).

Origin of replication module. Two successive site-directed saturation mutagenesis experiments (using primer pairs Ori-MlyI and Ori-AcuI; Supplementary Table S14) were performed to remove recognition sites for MlyI and AcuI in the origin of replication (*ori*) of pUC19 used as starting template. Successful removal of the recognition sequences was confirmed by the absence of restriction digest product with the corresponding enzyme. The final *ori* variant (*i.e.*, with no MlyI and AcuI) was then amplified with primers Ori-AflII and Ori-

SpeI (Supplementary Table S14), yielding the origin of replication module (framed by AflII and SpeI) for the pID vectors.

Ampicillin resistance selection and T7 expression modules. The sequences corresponding to the T7 expression (Supplementary Figure S4B) and AmpR cassettes (Supplementary Figure S4D) were synthesized by GenScript (NJ, USA). Position T8 of the T7 promoter was mutated to C to remove the MlyI site present in the natural promoter [9]. Silent mutations were introduced in the AmpR sequence to remove recognition sites for AcuI and FokI.

Assembly of pID-T7. The DNA cassettes corresponding to the modified *ori* (AflII /SpeI), AmpR (AflII /AatII) and the T7 expression module (AatII/SpeI) were isolated by double digestion with their corresponding restriction enzymes and agarose gel purification. A ligation reaction with 50 ng of each DNA fragment was then performed using T4 DNA ligase (Fermentas) overnight at 18˚C. After purification, the ligation products were transformed into electrocompetent *E. coli* Ecloni® 10G cells subsequently plated on LB-agar supplemented with 100 µg/mL ampicillin. The pID-T7 constructs extracted from the resulting transforming colonies were confirmed by restriction digestion profile and sequencing.

Generation and assembly of pID-Tet. TetR (encoding the Tet repressor) was amplified from pASK-IBA5plus (IBA Lifesciences) with primers TetR-F and TetR-B (Supplementary Table S14). AmpR was amplified from pID-T7 with primers mTEM1-F and mTEM1-B. The SpeI/AatII module for pID-Tet containing the AmpR-TetR operon was obtained by overlap PCR of these two products with primers mTEM1-F and TetR-B and subsequently inserted into pID-T7 at SpeI/AatII sites (replacing the AmpR cassette) to yield pID-T7-TetR. The Tet promoter sequence was amplified from pASK-IBA5plus with primers TetProm-F and TetProm-B and inserted into pID-T7-TetR at the AflII/NdeI sites (replacing the T7 promoter), yielding plasmid pID-Tet.

Supplementary Method S3. wtPTE reference sequence

>*wt*PTE
ATGGCCAGATGATTAATTCCTAATTTTTGTTGACACTCTATCATTGATAGAGTTATTTTACC
ACTCCCTATCAGTGATAGAGAAAAGTGAAATGAATAGTTCGACAAAAATCTAGAAATAATT
TTGTTTAACTTTAAGAAGGAGATATACATATGGCTAGCTGGAGCCACCCGCAGTTCGAAA
AAGGCGCCGGATCCTCC*ATGGGCGATCGGATCAATACCGTGCGCGGTCCTATCACAAT*
CTCCGAGGCGGGTTTCACACTAACCCACGAGCACATCTGCGGCAGCTCGGCAGGATTC
TTGCGTGCTTGGCCGGAGTTCTTCGGTAGCCGCAAAGCTCTAGCGGAAAAGGCTGTGA
GAGGATTGCGCCGCGCCAGAGCGGCTGGCGTGCGAACGATTGTCGATGTGTCGACTTT
CGATCTCGGTCGCGACGTTAGTTTATTGGCCGAGGTTTCGCGGGCTGCCGACGTTCATA
TCGTGGCGGCGACCGGCTTGTGGCTCGACCCGCCACTTTCGATGCGATTGAGGAGTGT
AGAGGAACTCACACAGTTCTTCCTGCGTGAGATTCAATATGGCATCGAAGACACCGGAA
TTAGGGCGGGCATTATCAAGGTCGCGACCACAGGCAAGGTGACCCCCTTTCAGGAGTTA
GTGTTAAGGGCAGCTGCCCGGGCCAGCTTGGCCACCGGTGTTCCGGTAACCACTCACA
CGGCAGCAAGTCAGCGCGGTGGTGAGCAACAAGCCGCCATTTTTGAATCCGAGGGCTT
GAGCCCCTCACGGGTTTGTATTGGCCACAGCGATGATACTGACGATTTGAGCTATCTCA
CCGCCCTCGCTGCGCGCGGATACCTCATCGGTCTAGACCATATTCCGCACAGTGCGATT
GGTCTAGAAGATAATGCGAGTGCATCAGCCCTCCTGGGTATTCGTTCGTGGCAAACACG
GGCTCTCTTGATCAAGGCGCTCATCGACCAAGGCTACATGAAACAAATCCTCGTTTCGAA

TGACTGGCTGTTCGGGTTTTCGAGCTATGTCACCAACATCATGGACGTGATGGATAGCG
TGAACCCCGACGGAATGGCCTTCATTCCACTGAGAGTGATCCCATTCCTACGAGAGAAG
GGTATTCCACAGGAAACGCTGGCAGGCATCACTGTGACTAACCCGGCGCGGTTCTTGTC
*ACCGACCTTGCGGGCGTCATGA*AGCTTGCTGCGGCACTCGAGCACCACCACCACCACC
ACTGAGATCCGGCTGCTAACAAAGCCCGAAAGGAAGCTGAGTTGGCTGCTGCCACCGC
TGA

The reference sequence contains the *wt*PTE gene (in italics) flanked by plasmid sequence (underlined). This longer sequence was used to obtain sufficient coverage at the ends of the gene.

Supplementary Method S4. NGS Step 1: Raw data processing

The processing of Illumina sequencing data shown here was performed using computational resources provided by University of Cambridge High Performance Computing (CSD3), but it can also be done on a personal computer. All scripts are available at https://github.com/fhlab/TRIAD.

The first part of analysis is done by the script *count.sh*. Briefly, the process consists of:

1. Assembly of paired-end reads into a single, longer read where possible, using PEAR v. 0.9.10 [10]. Through inspection of sequencing quality in FASTQ files and monitoring of assembly statistics, the options chosen were:

   --keep-original --min-overlap 5 --min-assembly-length 0 --quality-threshold 15 --max-uncalled-base 0.01

2. Create an index for the reference with Bowtie2 v.2.3.4 [11] and map both assembled and unassembled FASTQ reads, then sort resulting SAM files with samtools v.1.9 [12] to obtain the sequencing depth.

At this point, 95% of the reads aligned to reference sequence.

3. Based on tags in the SAM file, extract well-mapped reads and of those only keep the reads that contain mutations. Since this step detects any difference from reference, it will contain all reads with InDels as well as reads containing single point substitutions from sequencing errors. Hence, the number of substitutions in the final statistics is over-represented.
4. Since accurate identification of InDel position is essential for analysing transposon sequence preference, we use the deterministic Needleman-Wunsch algorithm to obtain the most accurate possible global alignment of the read to reference. Although using the alignment in the SAM file directly is faster, accepting a longer processing time at this stage is an acceptable trade-off to obtain accurate statistics of the library composition. The alignment was done with the Emboss 6.6.0 [13] implementation *needleall*, which compares many sequences to one.

The standard options for alignment were modified to gap open penalty 15 and gap extend penalty 0.5, in order to accurately identify long (9 bp or more) InDels. The default gap open

penalty (10) tends to split long InDels into several short InDels separated by one or two nucleotides.

Alternatively, the data can also be processed on a personal computer with the following modification: once reads are extracted from the SAM file, they should be filtered first for those that contain mutations. This reduces the size of resulting fasta and alignment files, which can otherwise exceed >10 GB. Development and testing of the scripts were done using this method on Linux Mint 18 in a virtual machine with two processor cores and 3 GB RAM. The corresponding code is available in *count_PC.sh.*

Supplementary Method S5. NGS Step 2: Parsing and statistics generation

The following analysis is done by script *PTE_composition.py* implemented in Python 3 with the following options:

--reference full_fragment.fa (sequence given in above in Supplementary Methods 2.3, plus flanking sequence from the plasmid)
--start_offset 200 (to ignore the preceding plasmid sequence)
--end_tail 97

1. Read in all FASTA multisequence alignment generated by previous step.
2. Create a dictionary containing all associated information: reference name, library name (intended as functional activity fraction or in this case, a multiplexed library), sequencing depth, change in DNA/protein terms, relevant counts. The information is nested with DNA variants nested under relevant protein variants, since multiple DNA variants can result in the same protein mutation. The variant information is stored both in internal format with a functional description (substitution/insertion/deletion/frameshift, used to generate statistics) and according to Human Genome Variation Standard.
3. Scan each pair of sequences (reference + aligned read) from the alignment, detect the mutation, translate to protein and add to dictionary.

Once the count dictionary is complete, it can be used to infer the following:

- Number of mutations per position (DNA or protein)
- Transposon consensus sequence for preferred insertion site
- Composition of insertions
- How many expected deletions/insertions (depending on the library) per DNA position are present

The data analysis, code to infer statistics and resulting figures are available in *TRIAD_composition_figures.ipynb*.

Supplementary Method S6. Treatment of point mutations

For reasons of computational efficiency, this pipeline focuses on reads that deviate from the reference. This difference may be a genuine mutation, a PCR error or a sequencing error. The resulting counts are therefore artificially enriched for many variants with a single nucleotide substitution, which each appears only once or perhaps twice. In Sanger sequencing (raw data not shown) we do not observe this kind of 'background noise', suggesting it should be disregarded in the NGS dataset. To corroborate this conclusion, we estimate the true number of point substitutions in the library by calculating the background substitution frequency from reads that align outside *wt*PTE, in the plasmid backbone, where no mutations were deliberately introduced during library construction. Such sequencing artefacts (with a single base pair substitution) occur in 3-4% of all reads, which corresponds to the error rate in the Illumina MiSeq NGS technology. An exact estimate is difficult due to a relatively low number of reads that align outside the fragment, as well as sequence dependence of polymerase errors – such that the error rate may be different in and out of the gene. Therefore, in the calculation of the proportion of frameshifts in the library, point mutations were simply counted as wild type (thus removing this 'noise').

Supplementary Method S7. Treatment of InDel redundancy

While pure substitution mutations can be placed very accurately, correct placement of InDels can be inherently ambiguous depending on the sequence context (as discussed in 1.2). InDels show some inherent redundancy, where distinct transposition and insertion / deletion events result in identical final sequence (Supplementary Figure S5B). For example, in the original sequence …nnGCTACTnn…, -3 bp deletions starting at position 2 (G---CT) and at position 3 (GC---T) result in the same final sequence: nnGCTnn (the remaining sequence context is abbreviated with *n*). The Needleman-Wunsch algorithm consistently (though arbitrarily) assigns this sequence to a deletion at position 2, such that any deletions originating from position 3 *cannot* be directly observed.

Implications:

- The raw counts that describe how many times a mutation was observed at which position, must be adjusted for this ambiguity, if we wish to infer the transposition sequence preference of the Mu transposon. The ambiguity can be partially corrected for deletions by generating a set of baseline reads that contain a -3 bp deletion at every *wt*PTE position and processing them in the same way as sequencing reads. Knowledge of these baseline counts allows us to split the observed counts in the -3 bp library across all originating positions. Data processed in this way was the basis of the frequency plot in Figure 3A.
- The diversity of mutations that can be *observed* is reduced compared to the maximal theoretical library diversity (Supplementary Figure S5). For example, in the -3 bp deletion library the theoretical diversity is one deletion per bp of gene length or 1000 variants for *wt*PTE, but the observable diversity due to ambiguity is 748 variants

(based on the particular sequence of the *wt*PTE gene). Hence, the -3 bp deletions actually observed by deep sequencing at 639 positions reflect 85% (=639/748) coverage of all possible variants, not 64% (=639/1000). Similarly, the maximum diversity of insertion libraries is less than maximal theoretical diversity at DNA level is 64 variants / triplet inserted / bp gene length. For all deletion and the +3 bp libraries, we calculated the theoretical diversity by computationally generating a perfect library (with script *baseline.py*), which contains every variant once (ie. 1 deletion of each length at each position, 1 insertion of each of the 64 codons at each positions), then processed this library in the same way as the NGS dataset. This shows the theoretical diversity in deletion libraries is ~0.75 deletion / bp gene length in *wt*PTE, while in the +3 bp library it is on average 46.22 variants / bp gene length.

In this manuscript, we focus on discussing the *observed* variants, rather than the number of variants inferred, so the InDel redundancy is generally not corrected. The exception is the discussion of Mu transposon sequence preference (Figure 3A).

Supplementary Method S8. Comparison of the effects of InDels *vs.* point substitutions on soluble expression and enzyme activity

Two TRIAD libraries (-3 bp and +3 bp) and the TriNEx library were transformed into *E. coli* BL21(DE3) (not containing pGro7). Overall, 192 transformants (corresponding to 40 deletion variants, 76 insertions variants and 76 substitution variants) were randomly picked and their plasmids were subjected to Sanger sequencing to discard frameshifted variants. As a result, 30 deletion, 27 insertion and 30 substitution variants were selected for further analysis of soluble expression and enzyme activity. The corresponding cells were transferred into 96-deep well plates for growth and expression as described in the Methods section of the main text. Paraoxonase activity was measured in diluted (1:1000 and 1:100) cell lysates (soluble fraction) as described there. Changes in soluble expression levels relative to parent *wt*PTE were determined by SDS-PAGE analysis of the soluble fractions. The OD_{600} was used to normalize the amount to analyse by SDS-PAGE for each sample. To determine the soluble expression change relative to the parent for each variant, the intensity of the protein band of interest was measured using ImageJ and standardized against that of *wt*PTE.

SUPPLEMENTARY FIGURES

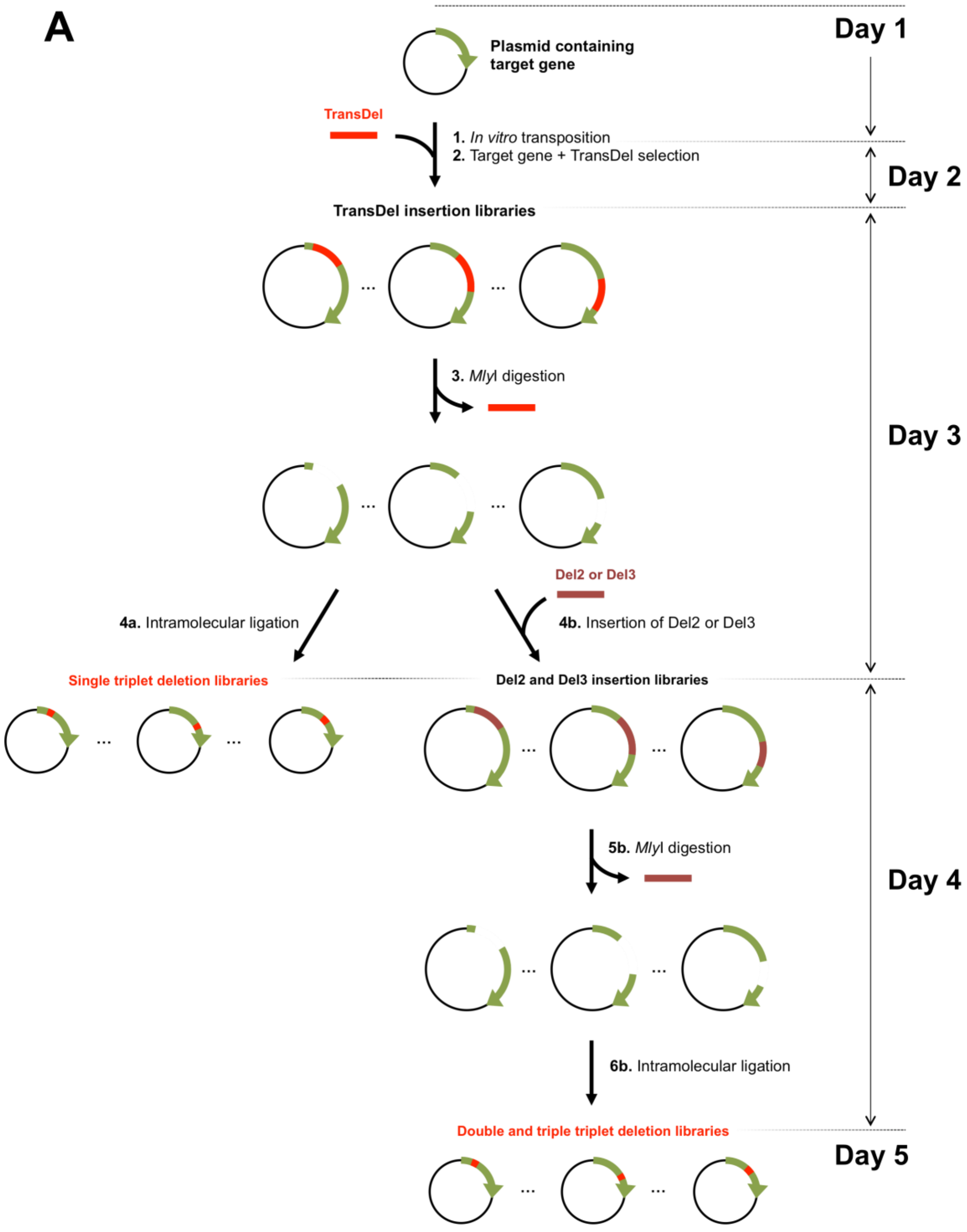

Supplementary Figure S1 (Continued on next page, legend follows).

(Figure S1 continued)

B

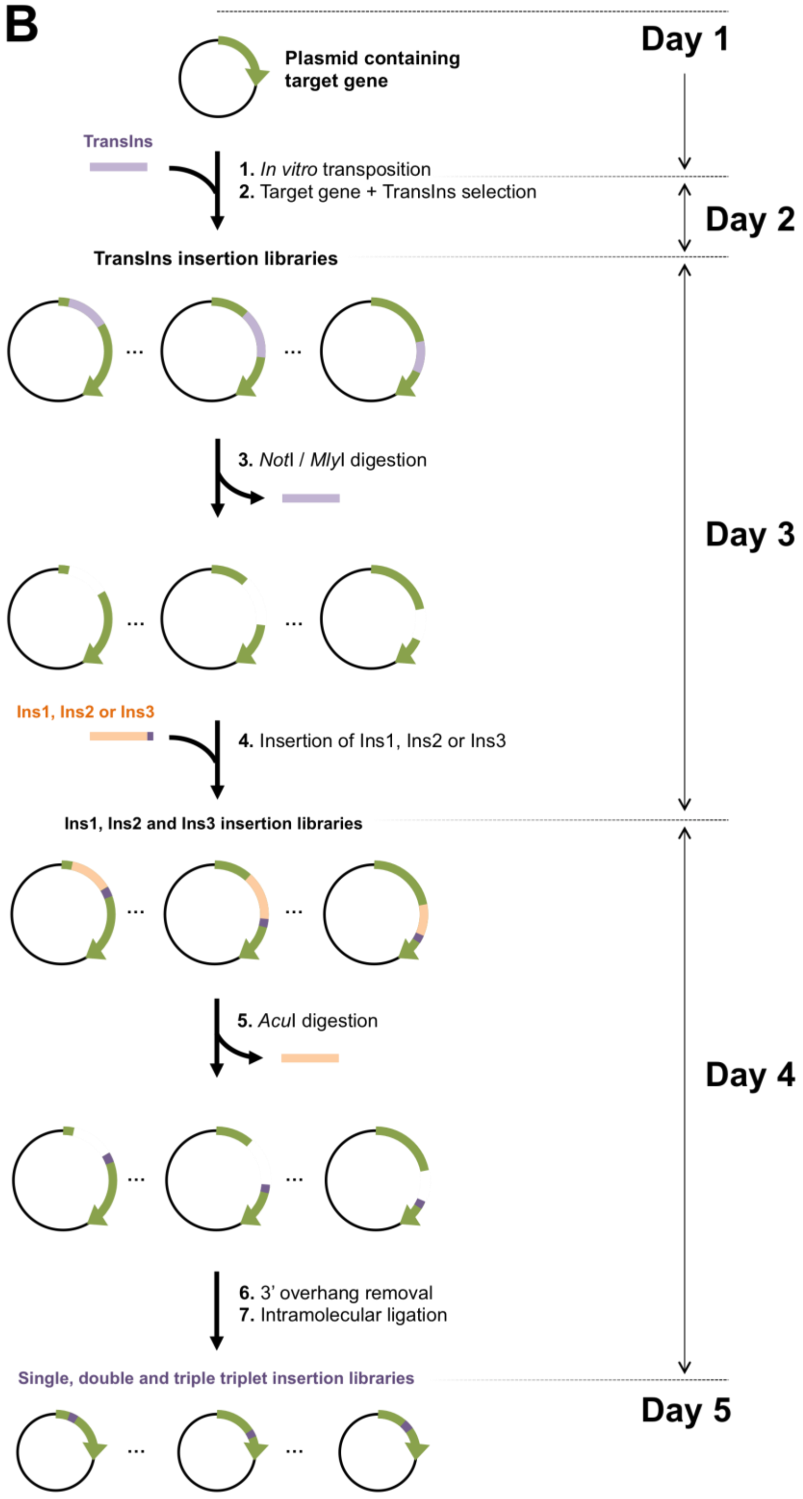

Supplementary Figure S1 (Continued on next page, legend follows).

(Figure S1 continued)

Supplementary Figure S1. Schematic outline and timeline of the procedure for the generation of random InDel libraries

A. Generation of deletion libraries.

Step 1: The TransDel insertion library is generated by *in vitro* transposition of the engineered transposon TransDel into the plasmid containing the target gene followed by the subcloning of the fragment comprising the target gene and the transposon into a fresh plasmid.

Step 2: MlyI digestion removes TransDel together with 3 bp of the original target gene and generates a single break per target gene variant.

Step 3a: Intramolecular ligation results in the reformation of the target gene minus 3 bp, yielding a library of single variants with a deletion of 1 triplet [1].

Step 3b: DNA cassettes dubbed Del2 and Del3 are then inserted between the break in the target gene to generate Del2 and Del3 insertion libraries.

Step 4b: MlyI digestion removes Del2 and Del3 together with 3 and 6 additional bp of the original GOI, respectively.

Step 5b: Intramolecular ligation results in the reformation of the target gene minus 6 and 9 bp, yielding libraries of single variants with a deletion of 2 and 3 triplets, respectively. Red vertical lines indicate deletions.

B. Generation of insertion libraries.

Step 1: The TransIns insertion library is generated by *in vitro* transposition of the engineered transposon into the target gene.

Step 2: digestion by NotI and MlyI removes TransIns.

Step 3: DNA cassettes dubbed Ins1, Ins 3 and Ins3 (with respectively 1, 2 and 3 randomized bp triplets at one of their extremities; indicated in blue) are then inserted between the break in the target gene to generate the corresponding Ins1, Ins2 and Ins3 insertion libraries.

Step 4: AcuI digestion and 5'end digestion by the Klenow fragment remove the cassettes, leaving the randomized triplet(s) in the original target gene.

Step 5: Intramolecular ligation results in the reformation of the target gene plus 3, 6 and 9 random bp, yielding libraries of single variants with an insertion of 1, 2 and 3 triplets, respectively. Purple vertical lines indicate insertions.

(A) Mu transposons TransDel and TransIns

BglII MlyI MuA binding site (R1/R2)

AGATCTGACTCGGCGCACGAAAAACGCGAAAGCGTTTCACGATAAATGCGAAAACTTTTCCCATGCATGGGAATAAATA

CCTGTGACGGAAGATCACTTCGCAGAATAAATAAATCCTGGTGTCCCTGTTGATACCGGGAAGCCCTGGGCCAACTTTT

Cat promoter

GGCGAAAATGAGACGTTGATCGGCACGTAAGAGGTTCCAACTTTCACCATAATGAAATAAGATCACTACCGGGCGTATT

Chloramphenicol acetyltransferase

TTTTGAGTTGTCGAGATTTTCAGGAGCTAAGGAAGCTAAAATGGAGAAAAAAATCACTGGATATACCACCGTTGATATA

(CamR)

TCCCAATGGCATCGTAAAGAACATTTTGAGGCATTTCAGTCAGTTGCTCAATGTACCTATAACCAGACCGTTCAGCTGG

ATATTACGGCCTTTTTAAAGACCGTAAAGAAAAATAAGCACAAGTTTTATCCGGCCTTTATTCACATTCTTGCCCGCCT

GATGAATGCTCATCCGGAATTACGTATGGCAATGAAAGACGGTGAGCTGGTGATATGGGATAGTGTTCACCCTTGTTAC

ACCGTTTTCCATGAGCAAACTGAAACGTTTTCATCGCTCTGGAGTGAATACCACGACGATTTCCGGCAGTTTCTACACA

TATATTCGCAAGATGTGGCGTGTTACGGTGAAAACCTGGCCTATTTCCCTAAAGGGTTTATTGAGAATATGTTTTTCGT

CTCAGCCAATCCCTGGGTGAGTTTCACCAGTTTTGATTTAAACGTGGCCAATATGGACAACTTCTTCGCCCCCGTTTTC

ACTATGGGCAAATATTATACGCAAGGCGACAAGGTGCTGATGCCGCTGGCGATTCAGGTTCATCATGCCGTTTGTGATG

GCTTCCATGTCGGCAGAATGCTTAATGAATTACAACAGTACTGCGATGAGTGGCAGGGCGGGGCGTAATGATATCGAGC

λ t_0 transcription terminator

TCGCTTTCTGTTGATAGATCCAGTAATGACCTCAGAACTCCATCTGGATTTGTTCAGAACGCTCGGTTGCCGCCGGGCG

SpeI MuA binding site (R1/R2)

TTTTTTATTGGTGAGAATCCAAGCACTAGTCGAGATCCGTTTTCGCATTTATCGTGAAACGCTTTCGCGTTTTTCGTGC

MlyI BglII

(TransDel) GCCGAGTCAGATCT

NotI BglII

(TransIns) GCGGCCGCAGATCT

(B) Deletion cassettes Del2 and Del3

SmaI MlyI EcoRI

(Del2) CCCGGGATGACTCCATGG

SmaI MlyI EcoRI

(Del3) CCCGGGACTCCATGG

ACTTCGCAGAATAAATAAATCCTGGTGTCCCTGTTGATACCGGGAAGCCCTGGGCCAACTTTTGGCGAAAATGAGACGT

Cat promoter

TGATCGGCACGTAAGAGGTTCCAACTTTCACCATAATGAAATAAGATCACTACCGGGCGTATTTTTTGAGTTGTCGAGA

Kanamycin nucleotidyltransferase (KanR)

TTTTCAGGAGCTAAGGAAGCTAAAATGATTGAACAAGATGGATTGCACGCAGGTTCTCCGGCAGCTTGGGTGGAGAGGC

TATTCGGCTATGACTGGGCACAACAGACAATCGGCTGCTCTGATGCCGCCGTGTTCCGGCTGTCAGCGCAGGGGCGCCC

GGTTCTTTTTGTCAAGACCGACCTGTCCGGTGCCCTGAATGAACTGCAAGACGAGGCAGCGCGGCTATCGTGGCTGGCC

ACGACGGGCGTTCCTTGCGCAGCTGTGCTCGACGTTGTCACTGAAGCGGGAAGGGACTGGCTGCTATTGGGCGAAGTGC

CGGGGCAGGATCTCCTGTCATCTCACCTTGCTCCTGCCGAGAAAGTATCCATCATGGCTGATGCAATGCGGCGGCTGCA

TACGCTTGATCCGGCTACCTGCCCATTCGACCACCAAGCGAAACATCGCATCGAGCGAGCACGTACTCGGATGGAAGCC

GGTCTTGTCGATCAGGATGATCTGGACGAAGAGCATCAGGGGCTCGCGCCAGCCGAACTGTTCGCCAGGCTCAAGGCGA

GCATGCCCGACGGCGAGGATCTCGTCGTGACCCACGGCGATGCCTGCTTGCCGAATATCATGGTGGAAAATGGCCGCTT

TTCTGGATTCATCGACTGTGGCCGGCTGGGTGTGGCGGACCGCTATCAGGACATAGCGTTGGCTACCCGTGATATTGCT

GAAGAGCTTGGCGGCGAATGGGCTGACCGCTTCCTCGTGCTTTACGGTATCGCCGCTCCCGATTCGCAGCGCATCGCCT

TCTATCGCCTTCTTGACGAGTTCTTCTGATATCGAGCTCGCTTTCTGTTGATAGATCCAGTAATGACCTCAGAACTCCA

λ t_0 transcription terminator SpeI MlyI SmaI

TCTGGATTTGTTCAGAACGCTCGGTTGCCGCCGGGCGTTTTTTATTGGTGAGAATCCAAGCACTAGTCGAGTCCCGGG

Supplementary Figure S2 (Continued on next page, legend follows).

(Figure S2 continued)

(C) Insertion cassettes Ins1, Ins2 and Ins3

MlyI Insert AcuI EcoRI

GAGTCAGCGC **(NNN)**$_n$ ATCCATCTCGAGTGGC**CTTCAG**CCATGGACTTCGCAGAATAAATAAATCCTGGTGTCCCTG

Cat promoter

TTGATACCGGGAAGCCCTGGGCCAACTTTTGGCGAAAATGAGACGTTGATCGGCACGTAAGAGGTTCCAACTTTCACCA

Kanamycin

TAATGAAATAAGATCACTACCGGGCGTATTTTTTGAGTTGTCGAGATTTTCAGGAGCTAAGGAAGCTAAAATGATTGAA

nucleotidyltransferase (KanR)

CAAGATGGATTGCACGCAGGTTCTCCGGCAGCTTGGGTGGAGAGGCTATTCGGCTATGACTGGGCACAACAGACAATCG

GCTGCTCTGATGCCGCCGTGTTCCGGCTGTCAGCGCAGGGGCGCCCGGTTCTTTTTGTCAAGACCGACCTGTCCGGTGC

CCTGAATGAACTGCAAGACGAGGCAGCGCGGCTATCGTGGCTGGCCACGACGGGCGTTCCTTGCGCAGCTGTGCTCGAC

GTTGTCACTGAAGCGGGAAGGGACTGGCTGCTATTGGGCGAAGTGCCGGGGCAGGATCTCCTGTCATCTCACCTTGCTC

CTGCCGAGAAAGTATCCATCATGGCTGATGCAATGCGGCGGCTGCATACGCTTGATCCGGCTACCTGCCCATTCGACCA

CCAAGCGAAACATCGCATCGAGCGAGCACGTACTCGGATGGAAGCCGGTCTTGTCGATCAGGATGATCTGGACGAAGAG

CATCAGGGGCTCGCGCCAGCCGAACTGTTCGCCAGGCTCAAGGCGAGCATGCCCGACGGCGAGGATCTCGTCGTGACCC

ACGGCGATGCCTGCTTGCCGAATATCATGGTGGAAAATGGCCGCTTTTCTGGATTCATCGACTGTGGCCGGCTGGGTGT

GGCGGACCGCTATCAGGACATAGCGTTGGCTACCCGTGATATTGCTGAAGAGCTTGGCGGCGAATGGGCTGACCGCTTC

CTCGTGCTTTACGGTATCGCCGCTCCCGATTCGCAGCGCATCGCCTTCTATCGCCTTCTTGACGAGTTCTTCTGATATC

λ t_0 transcription

GAGCTCGCTTTCTGTTGATAGATCCAGTAATGACCTCAGAACTCCATCTGGATTTGTTCAGAACGCTCGGTTGCCGCCG

terminator SpeI **AcuI** NotI

GGCGTTTTTTATTGGTGAGAATCCAAGCACTAGTCAGCAGCGA**CTGAAG**ACGGATGCGGCCGC

Supplementary Figure S2 (Continued on next page, legend follows).

(Figure S2 continued)

(D)

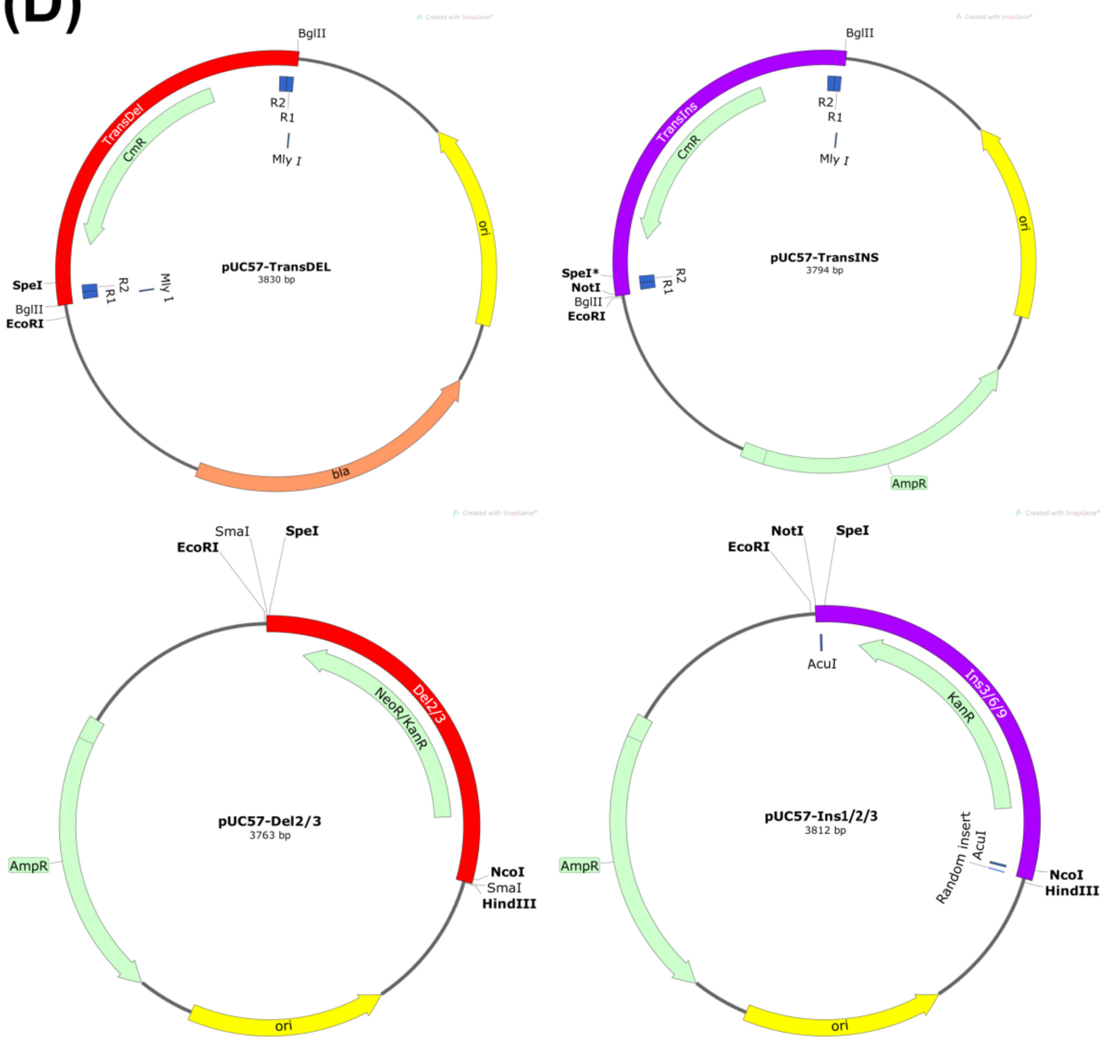

Supplementary Figure S2. Engineered transposons and cloning cassettes used in TRIAD.

Sequences of **(A)** Mu transposons TransDel and TransIns, **(B)** deletion cassettes Del2 and Del3, **(C)** insertion cassettes Ins1, Ins2 and Ins3 (n = 1, 2 or 3 nucleotide triplets), **(D)** Maps of vectors pUC-57-TransDel, -TransIns, -Del2/3, -Ins1/2/3. Note that in (C) the location of the KanR gene vs the NNN codons is identical to the depiction in Fig. 2B (where the restriction sites appear flipped around), but in both cases NNN codons appear next to the MlyI restriction site, which is *upstream* of the KanR expression cassette.

DNA sequence

NcoI

CC**ATG**GGCGATCGGATCAATACCGTGCGCGGTCCTATCACAATCTCCGAGGCGGGTTTCACACTAACCCACGAGCACATCTGCGGCA
GCTCGGCAGGATTCTTGCGTGCTTGGCCGGAGTTCTTCGGTAGCCGCAAAGCTCTAGCGGAAAAGGCTGTGAGAGGATTGCGCCGCG
CCAGAGCGGCTGGCGTGCGAACGATTGTCGATGTGTCGACTTTCGATCTCGGTCGCGACGTTAGTTTATTGGCCGAGGTTTCGCGGG
CTGCCGACGTTCATATCGTGGCGGCGACCGGCTTGTGGCTCGACCCGCCACTTTCGATGCGATTGAGGAGTGTAGAGGAACTCACAC
AGTTCTTCCTGCGTGAGATTCAATATGGCATCGAAGACACCGGAATTAGGGCGGGCATTATCAAGGTCGCGACCACAGGCAAGGTGA
CCCCCTTTCAGGAGTTAGTGTTAAGGGCAGCTGCCCGGGCCAGCTTGGCCACCGGTGTTCCGGTAACCACTCACACGGCAGCAAGTC
AGCGCGGTGGTGAGCAACAAGCCGCCATTTTTGAATCCGAGGGCTTGAGCCCCTCACGGGTTTGTATTGGCCACAGCGATGATACTG
ACGATTTGAGCTATCTCACCGCCCTCGCTGCGCGCGGATACCTCATCGGTCTAGACCATATTCCGCACAGTGCGATTGGTCTAGAAG
ATAATGCGAGTGCATCAGCCCTCCTGGGTATTCGTTCGTGGCAAACACGGGCTCTCTTGATCAAGGCGCTCATCGACCAAGGCTACA
TGAAACAAATCCTCGTTTCGAATGACTGGCTGTTCGGGTTTTCGAGCTATGTCACCAACATCATGGACGTGATGGATAGCGTGAACC
CCGACGGAATGGCCTTCATTCCACTGAGAGTGATCCCATTCCTACGAGAGAAGGGTATTCCACAGGAAACGCTGGCAGGCATCACTG
TGACTAACCCGGCGCGGTTCTTGTCACCGACCTTGCGGGCGTCA**TGA**AGCTT

HindIII

Protein sequence

MASWSHPQFEKGAGSSMG[34]DRINTVRGPITISEAGFTLTHEHICGSSAGFLRAWPEFFGSRKALAEKAVRGLRRARAAGVRTIVDVS
TFDLGRDVSLLAEVSRAADVHIVAATGLWLDPPLSMRLRSVEELTQFFLREIQYGIEDTGIRAGIIKVATTGKVTPFQELVLRAAAR
ASLATGVPVTTHTAASQRGGEQQAAIFESEGLSPSRVCIGHSDDTDDLSYLTALAARGYLIGLDHIPHSAIGLEDNASASALLGIRS
WQTRALLIKALIDQGYMKQILVSNDWLFGFSSYVTNIMDVMDSVNPDGMAFIPLRVIPFLREKGIPQETLAGITVTNPARFLSPTLR
AS[365]

Supplementary Figure S3: Sequence of the synthetic *wt*PTE gene and its corresponding protein product

This gene was designed without MlyI, AcuI and NotI restriction sites and cloned into pID-Tet or pET-strep vectors using NcoI and HindIII (underlined). Start and stop codons are shown in bold. The resulting protein (in red) was expressed in fusion with a Strep-tag II peptide (shown in green) at its N-terminus and its sequence corresponds to the one referred to as PTE-R0 in [14, 15] and as *wt*PTE in [8, 16]. Residues were numbered according to PDB 4PCP.

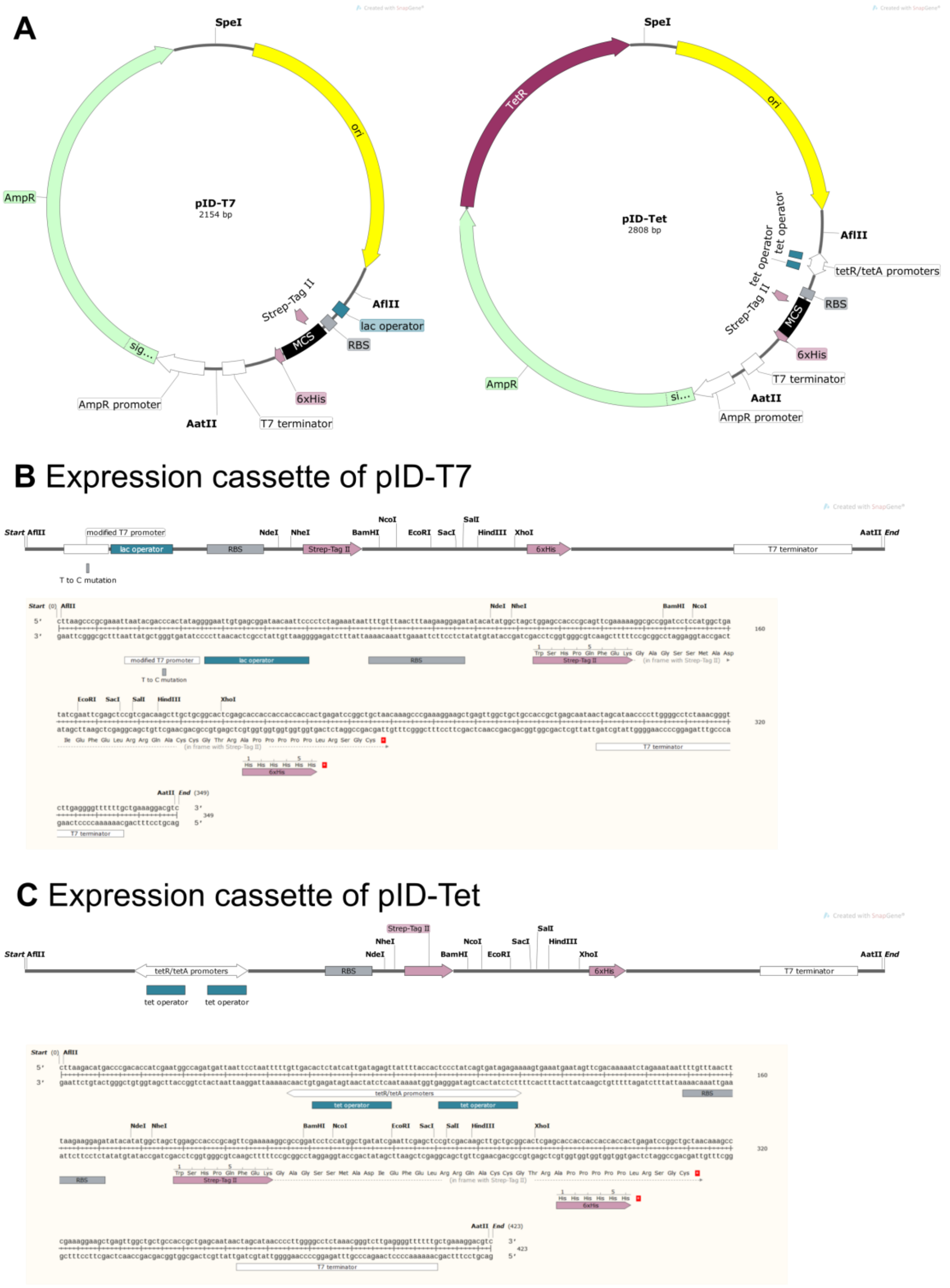

Supplementary Figure S4 (Continued on next page, legend follows).

(Figure S4 continued)

D AmpR cassette for pID vectors

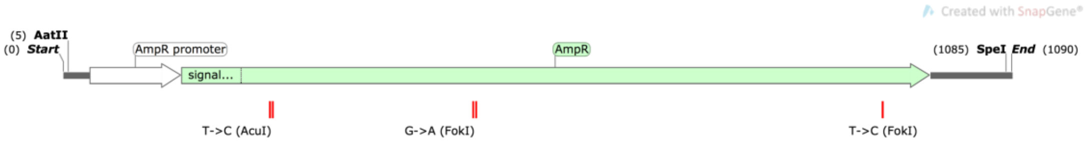


```
AatII                                AmpR promoter
GACGTCAGGTGGCACTTTTCGGGGAAATGTGCGCGGAACCCCTATTTGTTTATTTTTCTAAATAC

ATTCAAATATGTATCCGCTCATGAGACAATAACCCTGATAAATGCTTCAATAATATTGAAAAAGG
     AmpR
AAGAGTATGAGTATTCAACATTTCCGTGTCGCCCTTATTCCCTTTTTTGCGGCATTTTGCCTTCC
                                   (AcuI) CTGAAG
TGTTTTTGCTCACCCAGAAACGCTGGTGAAAGTAAAAGATGCCGAGGATCAGTTGGGTGCACGAG

TGGGTTACATCGAACTGGATCTCAACAGCGGTAAGATCCTTGAGAGTTTTCGCCCCGAAGAACGT

TTTCCAATGATGAGCACTTTTAAAGTTCTGCTATGTGGCGCGGTATTATCCCGTATTGACGCCGG

GCAAGAGCAACTCGGTCGCCGCATACACTATTCTCAGAATGACTTGGTTGAGTACTCACCAGTCA
         (FokI) GGATG
CAGAAAAGCATCTTACAGACGGCATGACAGTAAGAGAATTATGCAGTGCTGCCATAACCATGAGT

GATAACACTGCGGCCAACTTACTTCTGACAACGATCGGAGGACCGAAGGAGCTAACCGCTTTTTT

GCACAACATGGGGGATCATGTAACTCGCCTTGATCGTTGGGAACCGGAGCTGAATGAAGCCATAC

CAAACGACGAGCGTGACACCACGATGCCTGTAGCAATGGCAACAACGTTGCGCAAACTATTAACT

GGCGAACTACTTACTCTAGCTTCCCGGCAACAATTAATAGACTGGATGGAGGCGGATAAAGTTGC

AGGACCACTTCTGCGCTCGGCCCTTCCGGCTGGCTGGTTTATTGCTGATAAATCTGGAGCCGGTG

AGCGTGGGTCTCGCGGTATCATTGCAGCACTGGGGCCAGATGGTAAGCCCTCCCGTATCGTAGTT
                      (FokI) GGATG
ATCTACACGACGGGCAGTCAGGCAACTATGGACGAACGAAATAGACAGATCGCTGAGATAGGTGC

CTCACTGATTAAGCATTGGTAACTGTCAGACCAAGTTTACTCATATATACTTTAGATTGATTTAA
                                             SpeI
AACTTCATTTTTAATTTAAAAGGATCTAGGTGAAGATCCTTTTTACTAGT
```

Supplementary Figure S4: Vectors for the generation of InDel variant libraries

These vectors are designed for the construction of TRIAD libraries and expression of the generated variants (feature not used in the present study). They were designed and assembled (see supplementary methods) from different components separated by three restriction sites: (*i*) ampicillin resistance gene (AmpR; AatII/SpeI) forming an operon with TetR (encoding the tetracycline repressor) in the case of pID-Tet, (*ii*) origin of replication (ori; SpeI/AflII), and (*iii*) expression cassette (AflII/AatII) consisting of a promoter (T7 and Tet for pID-T7 and pID-Tet, respectively), a multiple cloning site (MCS), sequences encoding affinity tags (*i.e.* Strep-tag II and 6xHis-tag) and the T7 terminator sequence. These vectors do not contain any MlyI, AcuI and NotI restriction sites in their sequence. **(A)** Maps of vector pID-T7

and pID-Tet. **(B)** Map and sequence of expression cassette for pID-T7, where position T8 of the T7 promoter was mutated to C to remove MlyI site as in [9]. **(C)** Map and sequence of expression cassette for pID-Tet. **(D)** Map and sequence of AmpR cassette (silent mutations to remove AcuI and FokI sites are indicated).

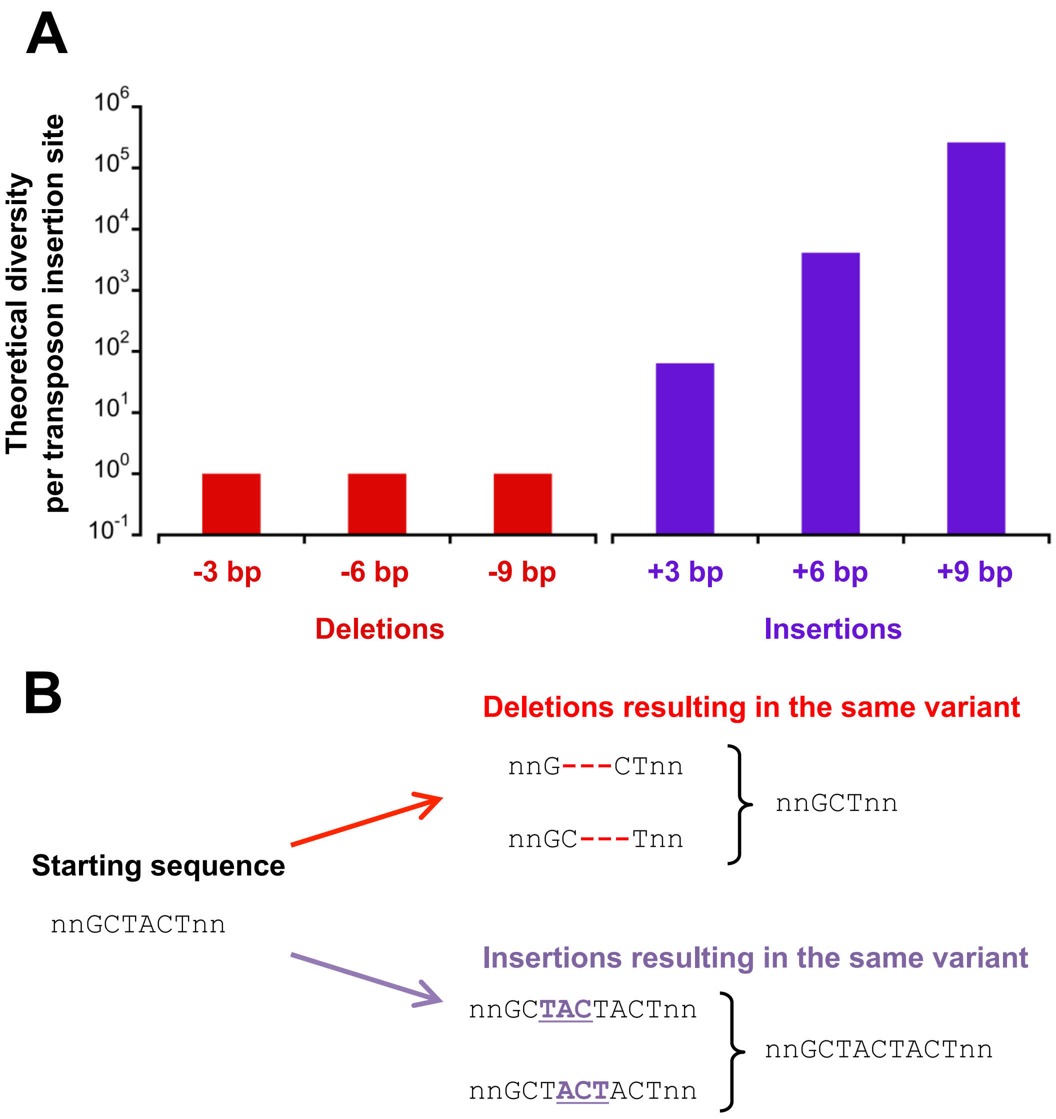

C

	Deletions			Insertions	
	**-3 bp**	**-6 bp**	**-9 bp**	**+3 bp**	**+6 bp**
**Theoretical diversity**					
at the DNA level	748	747	730	46170	2844330
at the protein level	590	589	551	13004	268159

Supplementary Figure S5: Theoretical diversities of the InDel libraries obtained with TRIAD

(A) This plot shows the theoretical diversity per transposon insertion site within the target gene for each type of InDel generated using TRIAD. Only one deletion, regardless of its length, can occur at a given transposition insertion site. Conversely, insertion diversity is related to the length of the randomized inserted triplet nucleotides (4^3, 4^6 and 4^9 for one, two and three triplet insertions, respectively). Transposon insertion can occur at each position of the target DNA, providing it does not affect the restriction sites that are necessary in the

various subcloning steps of the TRIAD procedure. Therefore, the theoretical diversity for each TRIAD library is obtained by multiplying the values plotted in this figure with the length of the target DNA. In the case of a target gene with the length of *wt*PTE (~1,000 bp), the theoretical diversity for deletion libraries is ~10^3 while it is 6.4×10^4, ~4.1×10^6 and ~2.6×10^8 for one, two and three triplet insertions, respectively. **(B)** Examples of InDel redundancy in the case of deletions (-3 bp) or insertions (+3 bp) of a triplet resulting in the same DNA variants. **(C)** Theoretical diversities accessible by applying TRIAD to the *wt*PTE gene sequence in TRIAD libraries -3, -6, -9, +3 and +6 bp. The diversities were determined computationally by using the python script *baseline.py* to generate fasta reads with all possible mutations, which were then aligned and counted in the exact same way as the physical libraries.

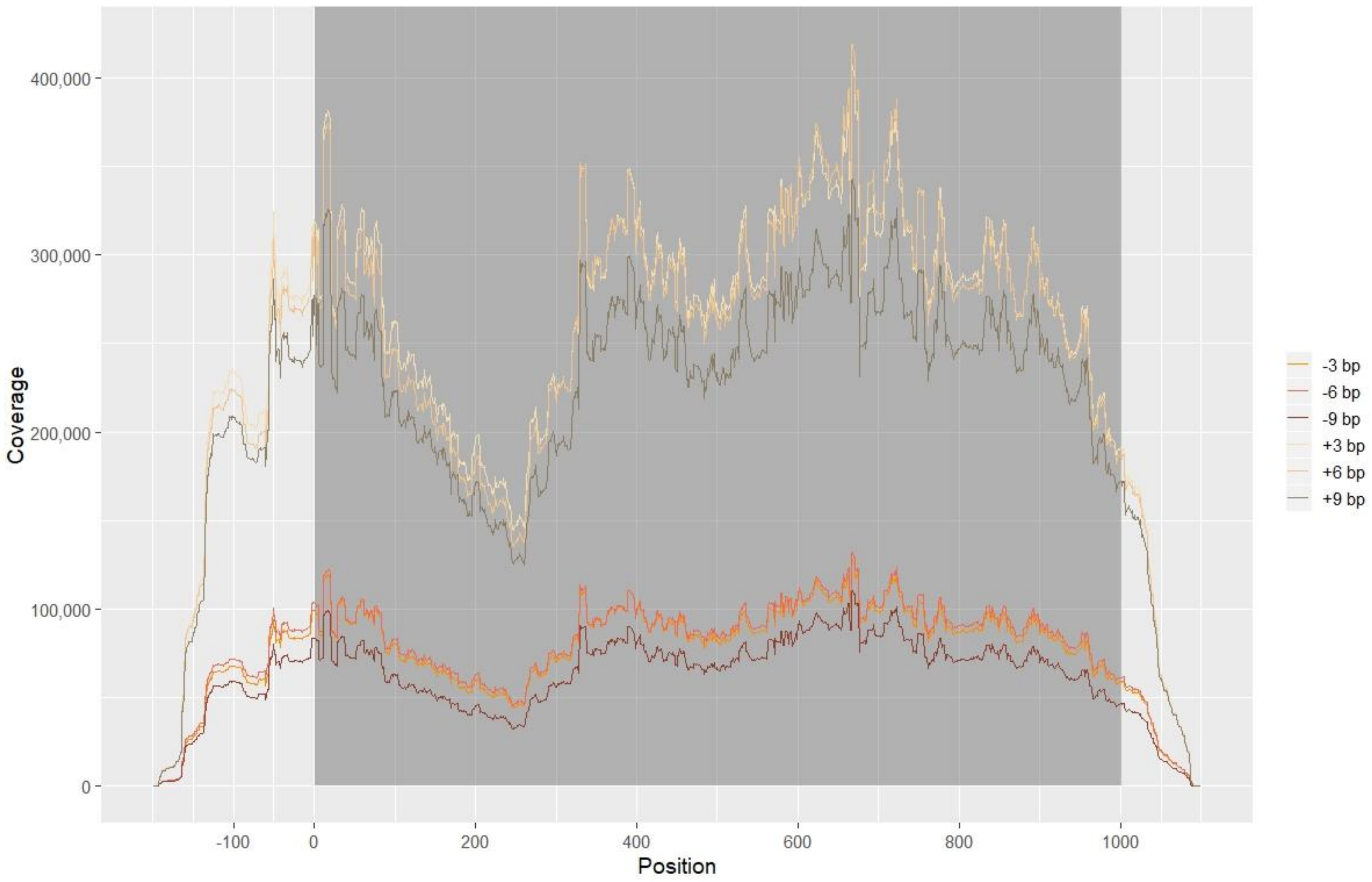

Supplementary Figure S6 (legend next page)

Supplementary Figure S6: Sequencing coverage in the NGS of the TRIAD libraries of *wt*PTE.

The final total depth from all assembled and unassembled reads that map to the reference is shown along the entire sequencing fragment. The proportion of reference DNA corresponding to *wt*PTE gene is shown on shaded background. Coverage for insertion libraries is approximately 3× that of deletion libraries, due to higher loading onto the MiSeq flow-cell in order to capture the higher diversity of insertion libraries better. Despite a decrease in coverage around position 200, good coverage is maintained across the entire *wt*PTE gene.

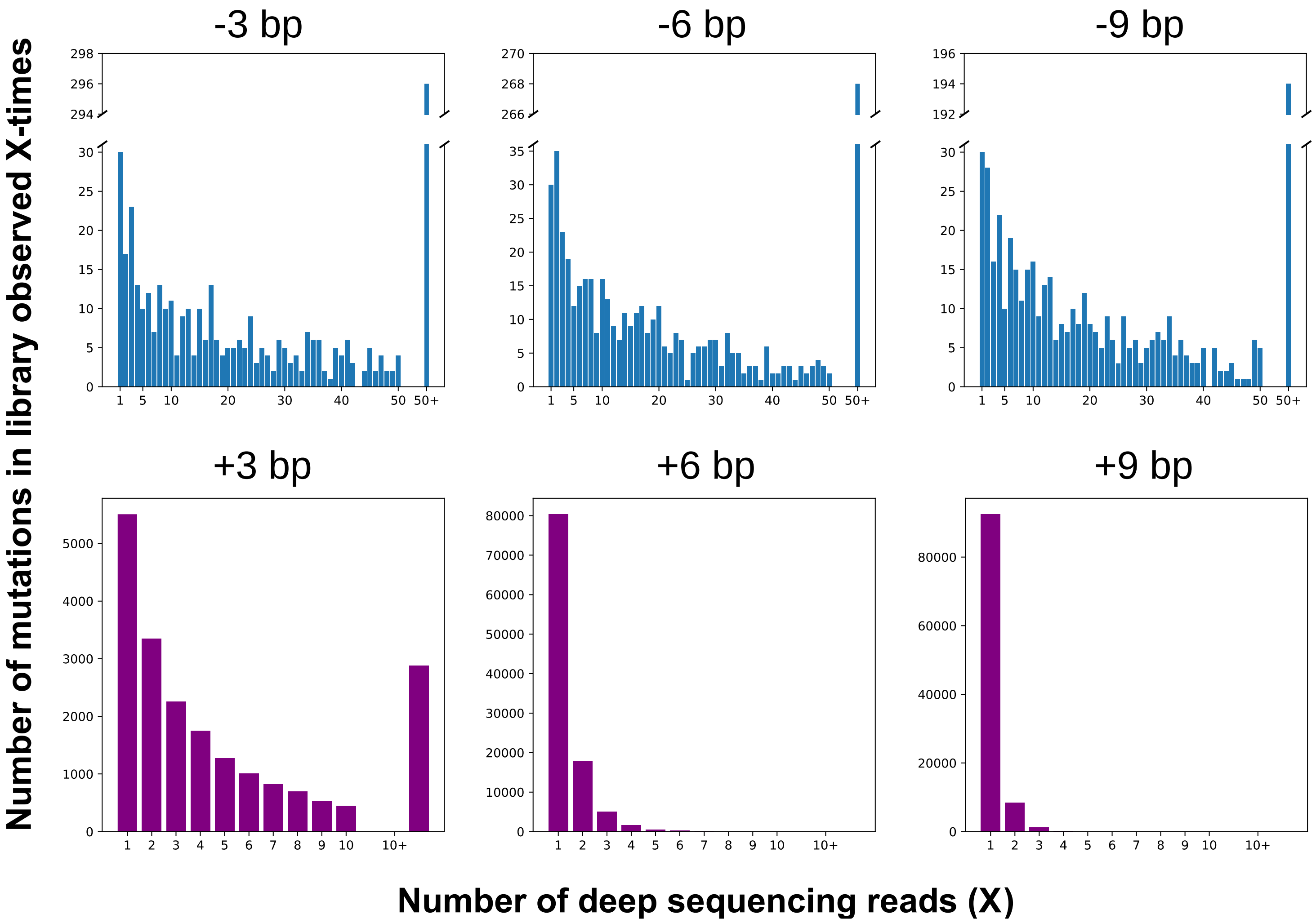

Supplementary Figure S7 (legend next page)

Supplementary Figure S7: Distribution of observed number of reads per mutation.

The histograms show how many mutations are observed once, twice, thrice, ten times or more. In deletion libraries we find that most mutations detected are supported by 10-40 observations (each observation is a single read in raw sequencing data).

The bias of transposon site preference results in InDels being observed more often at some positions than other. In the deletion libraries, most mutations are supported by < 50 reads (see Table S6), but there is a long tail generated by positions that are close to the Mu transposon consensus – this is aggregated into one bin in the histograms in this Figure for clarity. Because of the large diversity of insertion libraries, variants are generally observed with lower frequencies (x-axis) compared to the deletion libraries.

A

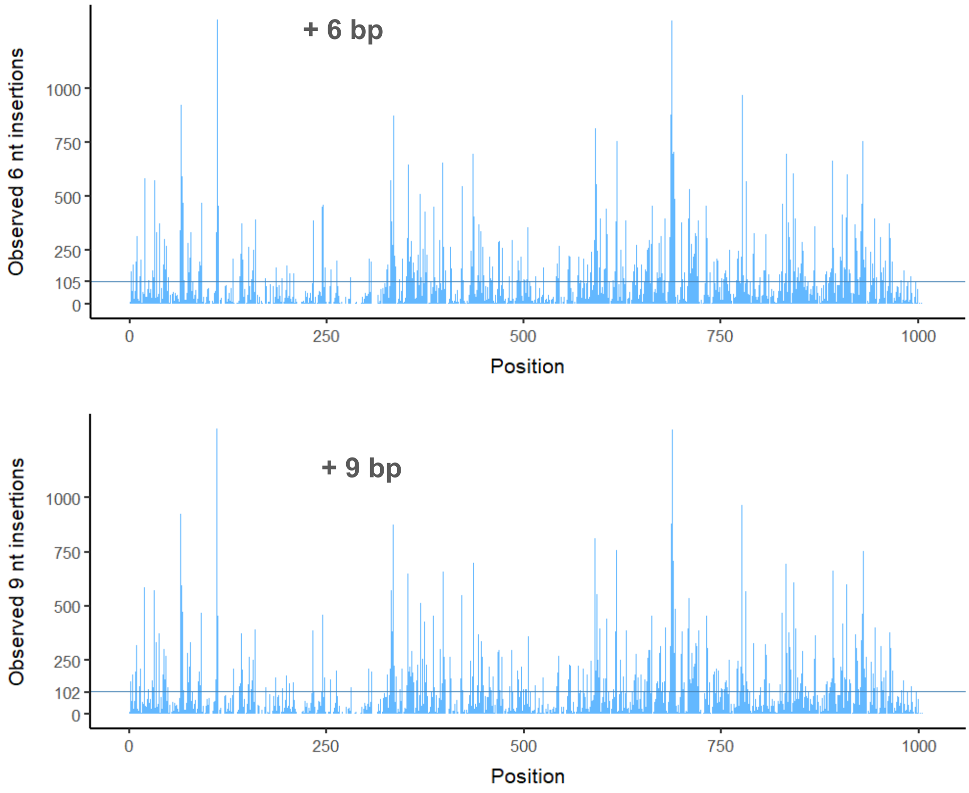

Supplementary Figure S8 (Continued on next page, legend follows).

(Figure S8 continued)

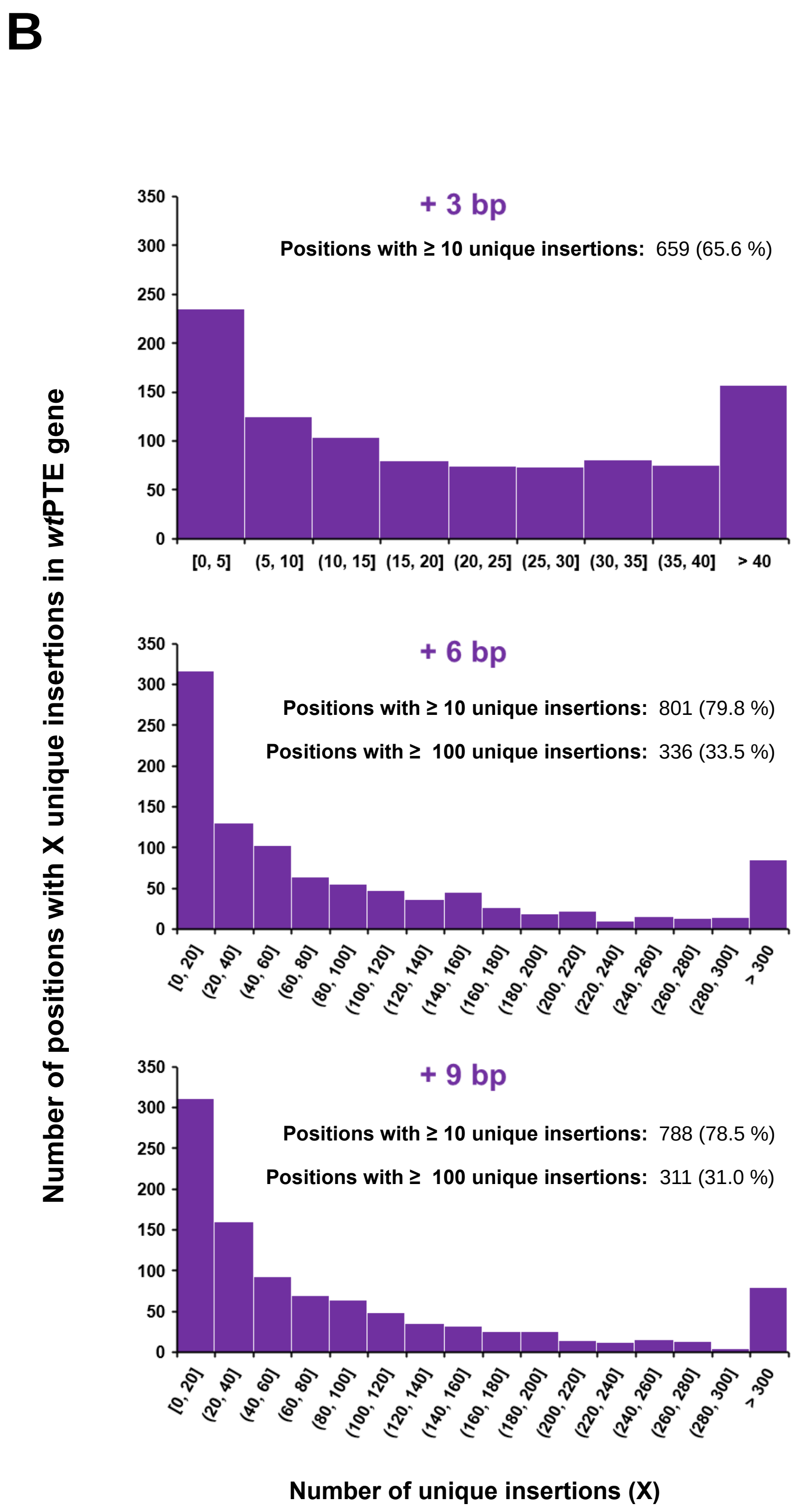

Supplementary Figure S8 (legend next page)

Supplementary Figure S8: Number of distinct insertions observed per position in *wt*PTE.

(A) Distribution and number of distinct (or unique) insertions per DNA position determined by deep sequencing in +6 bp and + 9 bp bp libraries., compared to the mean per position.In analogy to Figure 3C in the main text, this figure shows the number of observed insertions at each position where mutations are observed. The possible diversity of +6 bp and +9 bp insertions is much higher than for +3 bp library, which results in more pronounced high diversity “spiked” at positions where transposon insertion is favoured. The horizontal line shows the mean number of observed insertions per position (105 and 102 for +6 bp and +9 bp, respectively). These results are not corrected for codon ambiguity, which increases the unevenness of the distribution.

(B) Distribution of the number of positions in the gene encoding *wt*PTE *versus* the number of distinct insertions observed by deep sequencing.

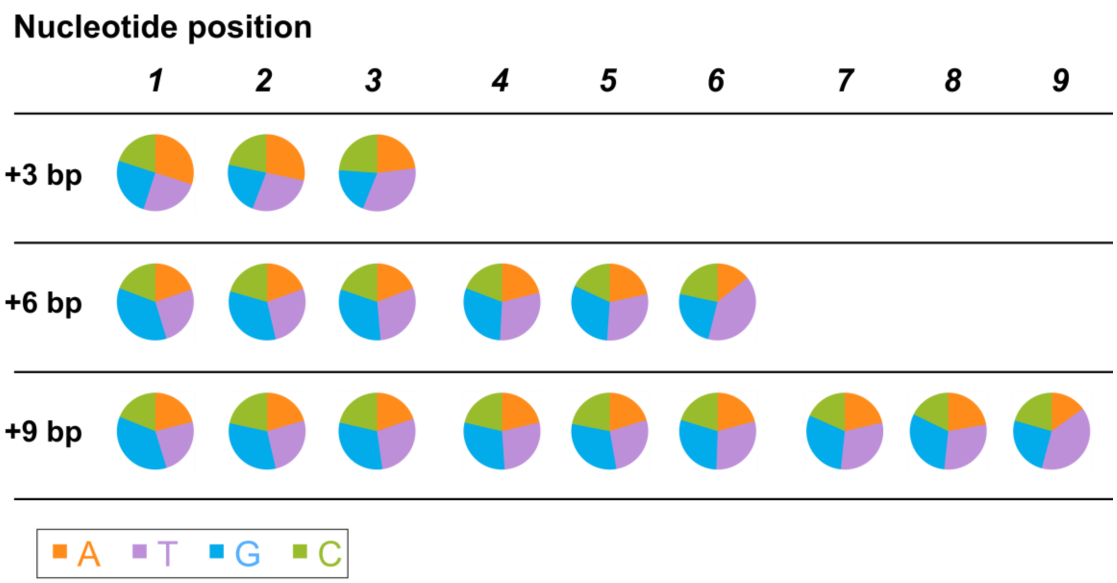

Supplementary Figure S9: Distribution of nucleotide bases in the randomized inserts of the +3, +6 and +9 bp libraries

The nucleotide percentage distributions of the in-frame insertions observed among all *wt*PTE variants observed during deep sequencing. Every detected insertion contributes equally to this distribution, regardless of frequency in the library.

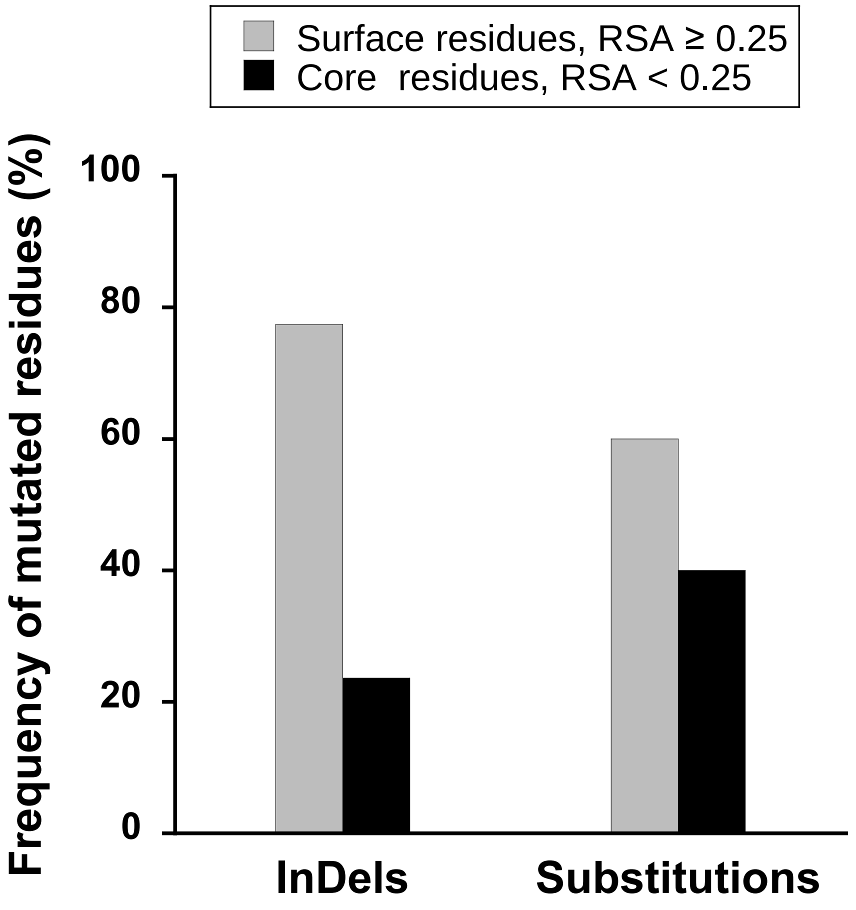

Supplementary Figure S10: Relationship between mutational tolerance and solvent-accessible surface area (SASA) in *wt*PTE

The solvent accessible surface area (SASA) of residues mutated (either InDel or substitution) in *wt*PTE variants retaining ≥50% of the parental paraoxonase activity was calculated from the structure of *wt*PTE (PDB code: 4PCP) using the PISA web server at the European Bioinformatics Institute (http://www.ebi.ac.uk/pdbe/prot_int/pistart.html) [17]. Relative accessible surface area (RSA) was defined as the ratio of the SASA for a given residue within the structured protein *vs*. in the free residue [18]. Residues were classified as core for RSA < 0.25, and surface for RSA ≥ 0.25 [19]. Mutated residues are listed in Supplementary Table S10.

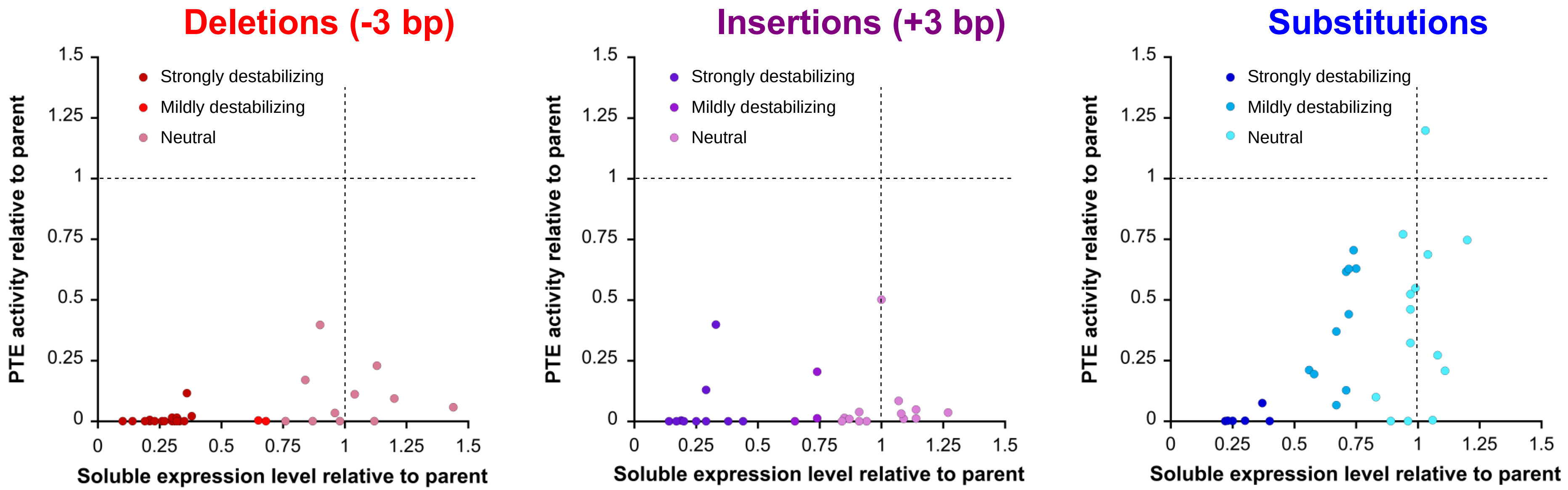

Supplementary Figure S11: Comparison of the effects on activity and solubility (*i.e.*, kinetic stability) between InDel (from TRIAD libraries -3 bp and +3 bp) and substitution variants (from TriNEx library) of wtPTE.

Upon transformation of the DNA libraries into *E. coli* BL21(DE3), a total of 192 colonies (64 per library) were selected randomly, and their corresponding PTE variants sequenced to discard the ones with frameshifting mutations. The appropriate number of variants with non-frameshifting mutations were then screened for phosphotriesterase activity and soluble expression in the absence of GroEL/ES.Changes in paraoxonase (PTE) activity are determined relative to those of *wt*PTE by comparing the initial rates in cell lysates measured under identical conditions with 200 µM of the native paraoxon substrate (see Methods section and Tables S11-12). Relative soluble expression levels are determined as the ratio in protein expression levels in the clear lysate (supernatant) between variants and the parent enzyme *wt*PTE measured by SDS-PAGE (See Methods and Tables S11-12).

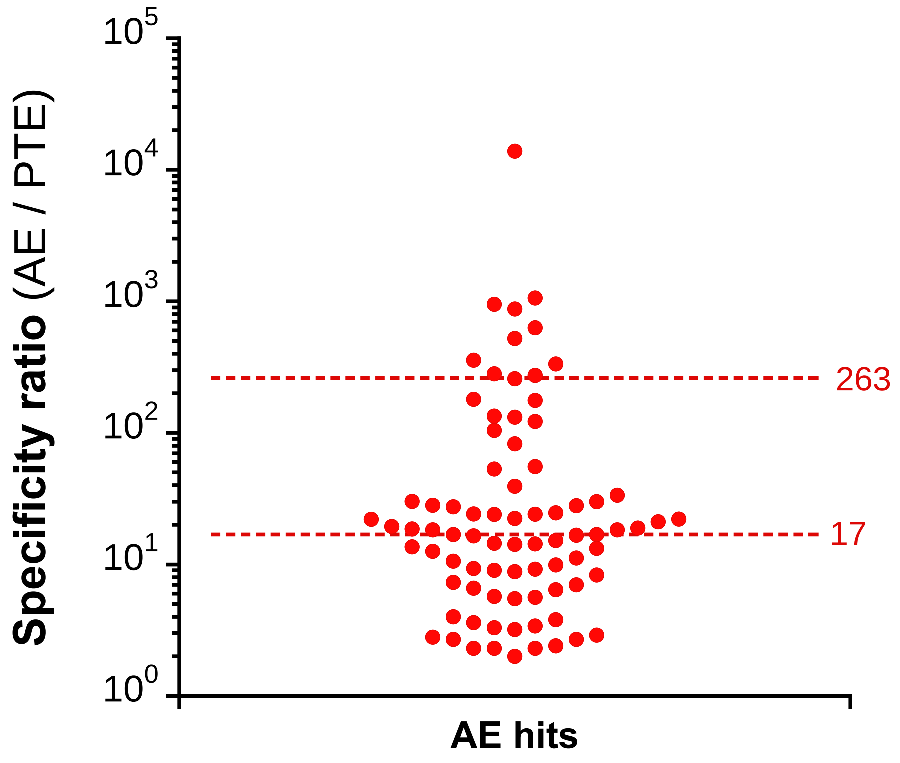

Supplementary Figure S12: Activity trade-offs in *wt*PTE InDel variants improved in arylesterase activity.

Activity trade-offs among InDel *wt*PTE variants improved in arylesterase activity (AE) hits (Supplementary Table S13) were evaluated by calculating the specificity ratio, *i.e.* the ratio between the level of AE activity in cell lysate and that of phosphotriesterase activity (PTE). This plot shows the specificity ratio for each InDel variants listed in Supplementary Table S13. The average (~260) and median (~17) specificity ratios are also indicated.

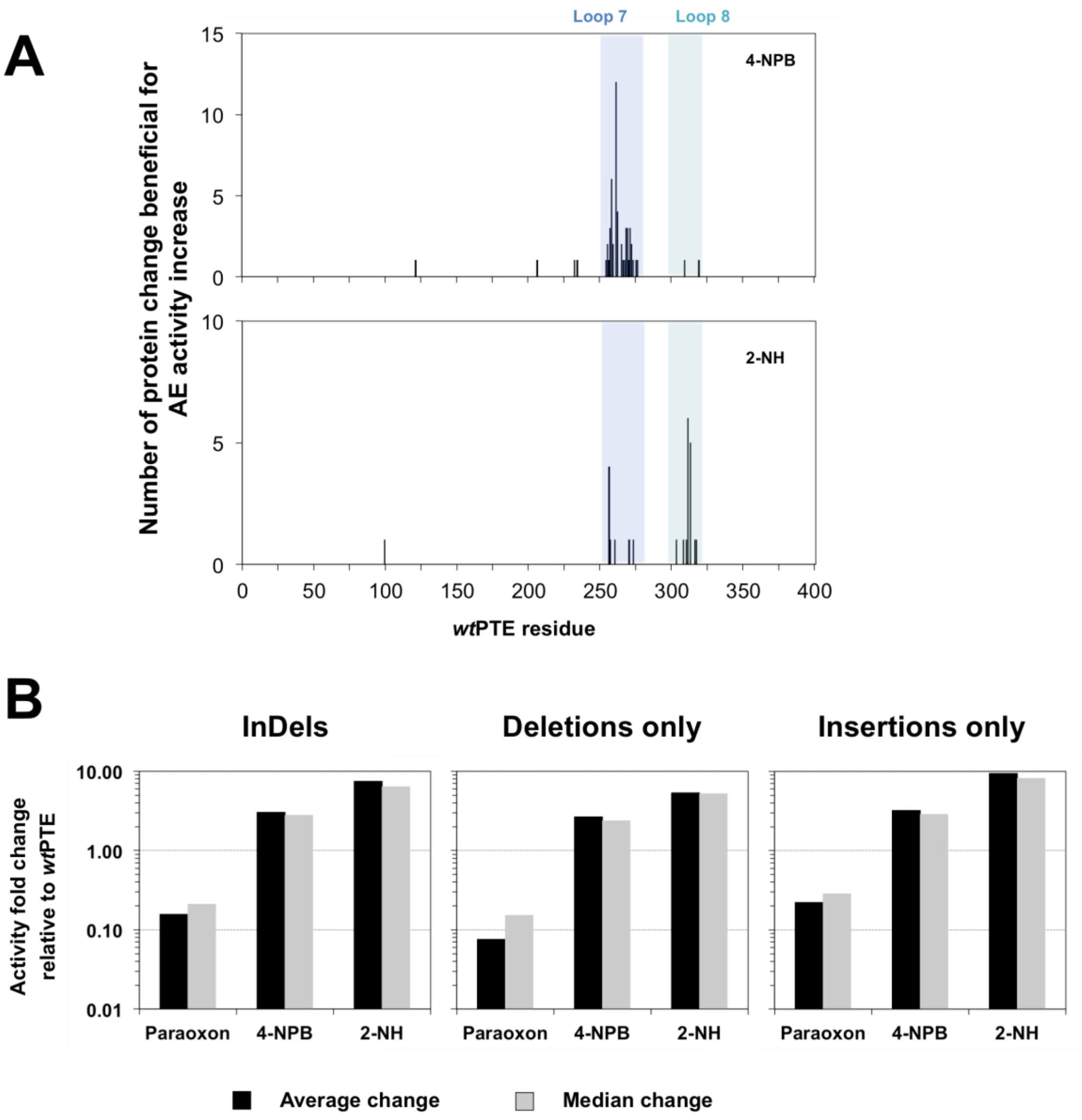

Supplementary Figure S13: Insertions and deletions improving the arylesterase activity of *wt*PTE by > 2-fold.

(A) Location and occurrence of adaptive insertions and deletions in *wt*PTE. InDels improving arylesterase activity (AE) towards 4-nitrophenyl butyrate (4-NPB; top plot) and 2-naphthyl hexanoate (2-NH; bottom plot) are shown according to their location in the *wt*PTE sequence and the number of their occurrences.

(B) Average and median activity change in AE-improved *wt*PTE variants. Values refer to the activity change of all AE-improved variants relative to wtPTE obtained by comparing the initial rates v_0 for the hydrolysis of paraoxon (PTE), 4-NPB or 2-NH to that of wtPTE at 200 μM substrate concentration, resulting in a dimensionless ratio. The average change value was determined as the geometric mean of the relative activities of the variants listed in Supplementary Table S13 and the median change corresponds to the relative activity lying at the midpoint of the recorded relative activities.

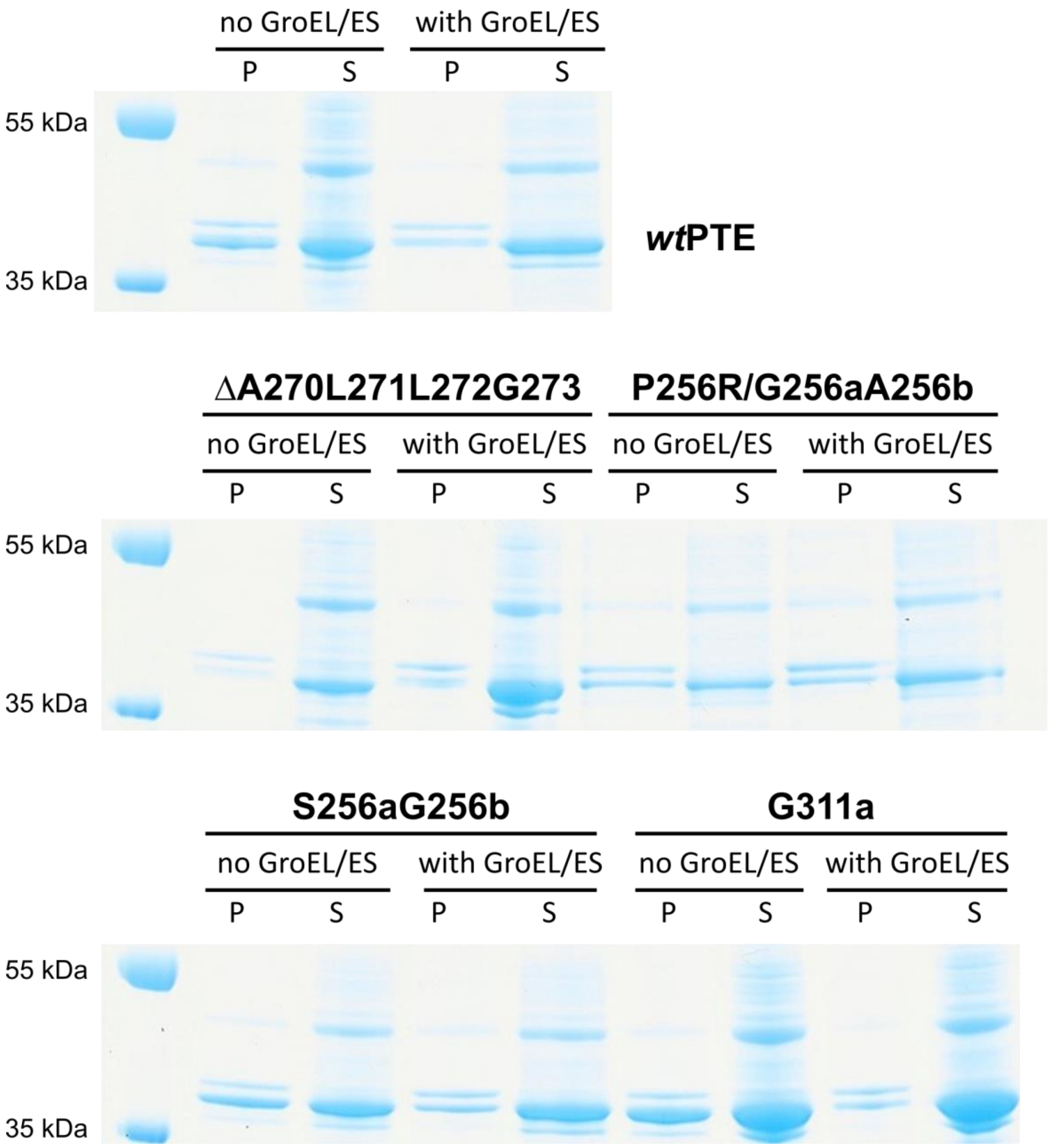

Supplementary Figure S14: Soluble and insoluble expression of PTE hits in the presence and absence of GroEL/ES chaperones.

Screening of InDel (TRIAD) libraries of *wt*PTE variants for increased arylesterase activity was performed in the presence of co-expressed GroEL/ES chaperones to buffer destabilizing and adaptive mutations. A comparison of PTE expression in E. coli lysates (P: pellet; S; soluble fraction) of the parent *wt*PTE and four characterized hits (see Table 3) shows slight increases of PTE expression in the presence of chaperone (see Table S14 for a quantitation of the band intensity). These suggest that the inclusion of GroEL/ES chaperones was helpful, but not crucial for the success of the experiment. The gels images are a representative representation of an experiment conducted in triplicate.

(A) **PTE** **AE**

∆A270L271L272G273

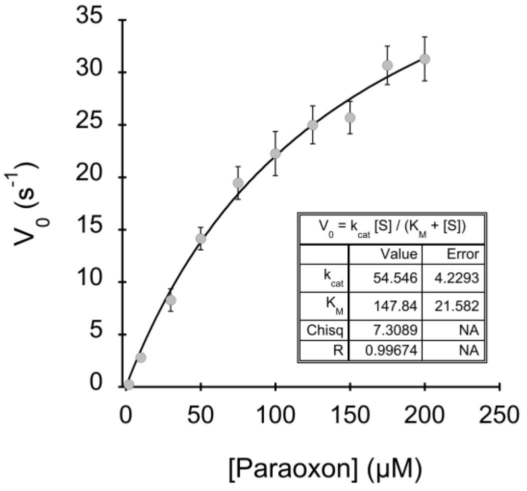

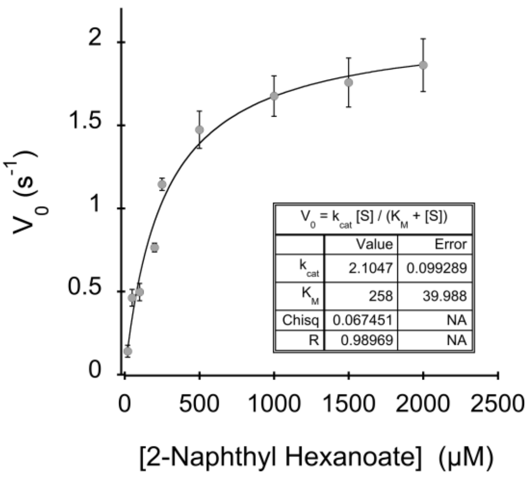

P256R/G256aA256b

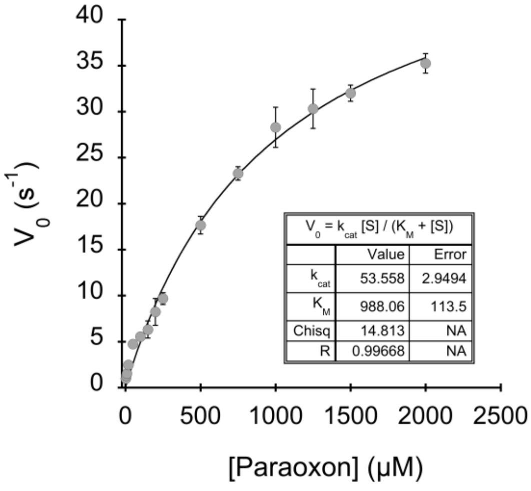

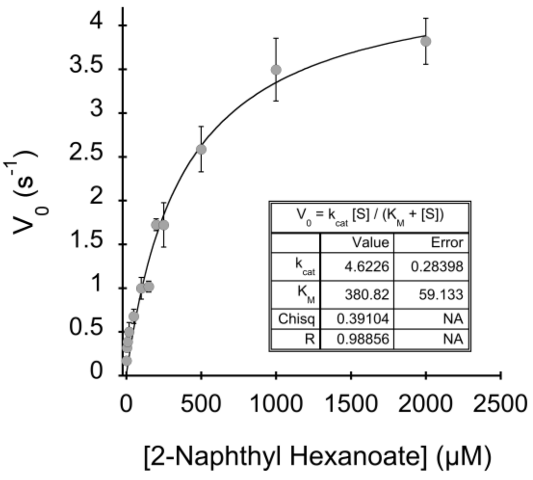

S256aG256b

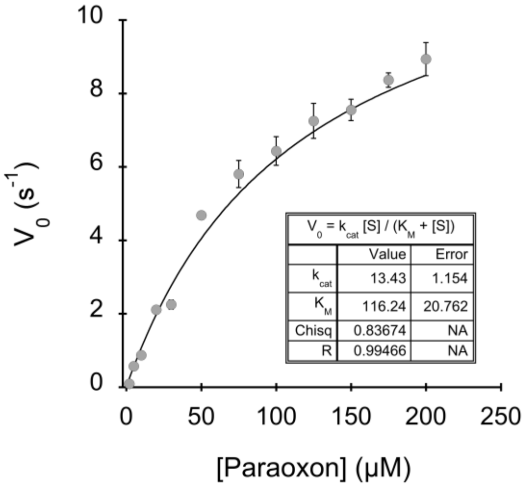

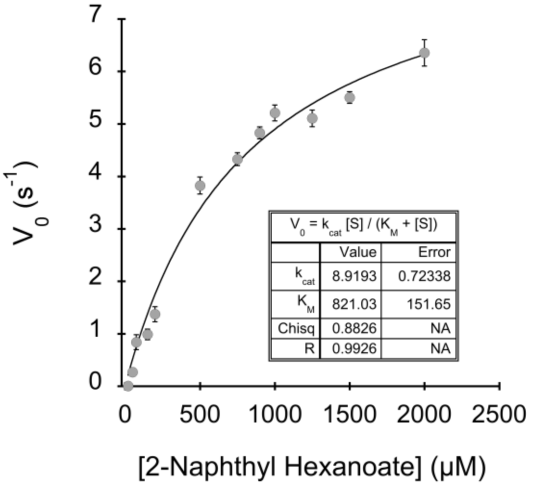

Supplementary Figure S15 (Continued on next page, legend follows)

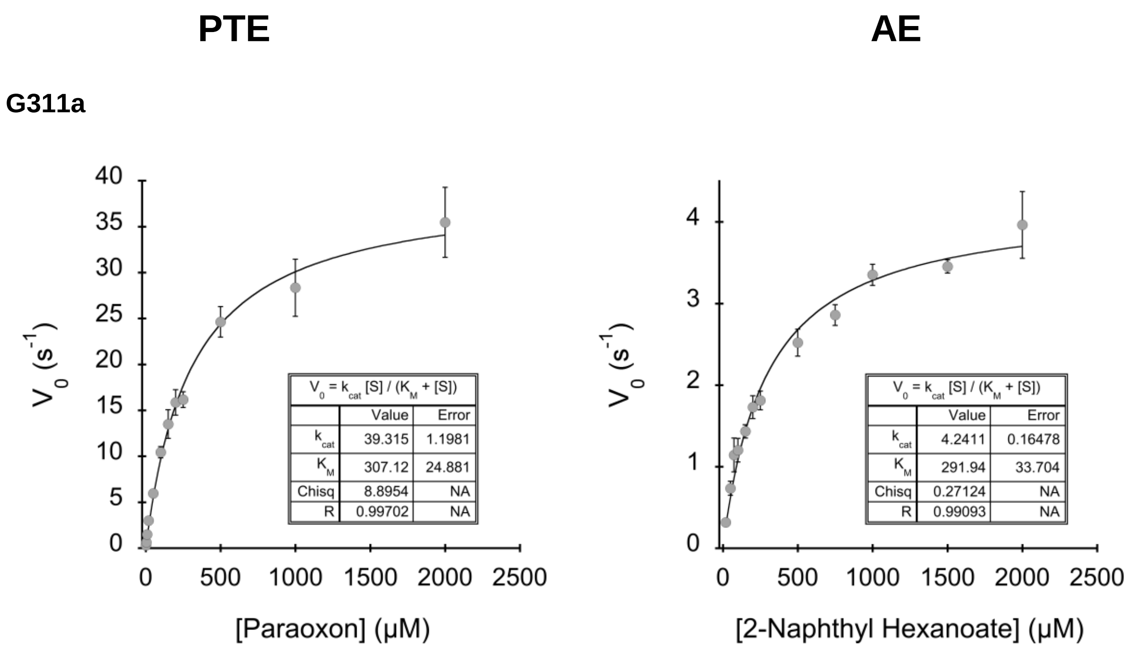

(B)

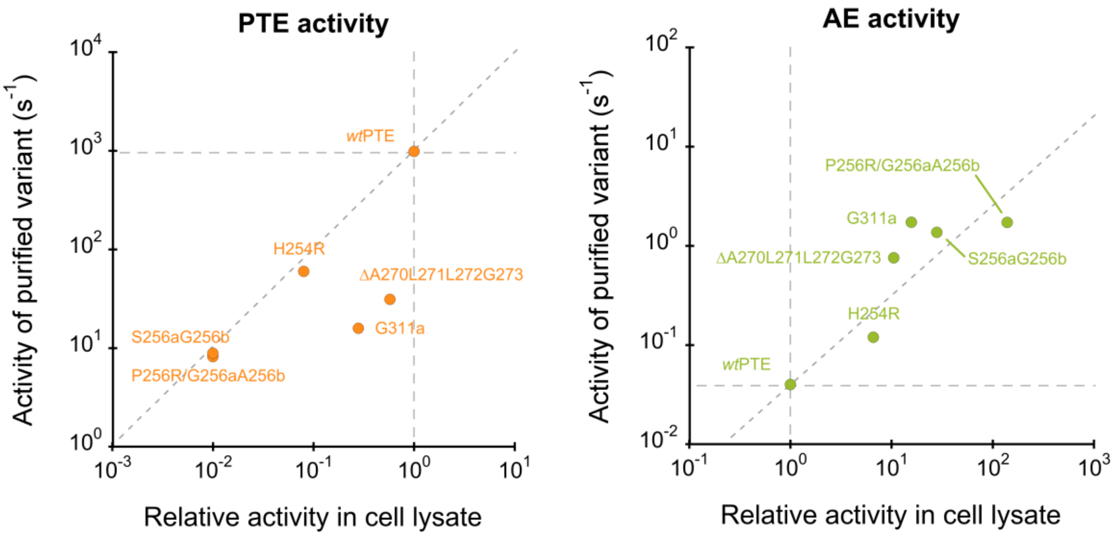

Supplementary Figure S15: Kinetic characterization of InDel *wt*PTE variants with improved arylesterase activity.

(A) Michaelis-Menten plots for purified AE InDel hits. Phosphotriesterase (PTE; substrate: paraoxon) and arylesterase (AE; substrate: 2-Naphthyl Hexanoate) activities were measured in triplicates and error bars show standard deviation. Conditions: [S]=0–2 mM; [Tris-HCl] = 100 mM (pH 7.5); [$ZnCl_2$] = 200 μM; Enzyme concentrations: for PTE activity, [Enzyme] = 5 (for P256R/G256aA256b and G311a) or 10 nM (for ΔA270L271L272G273 and S256aG256b); for AE activity, [Enzyme] = 200 nM (for ΔA270L271L272G273, P256R/G256aA256b and S256aG256b)

or 20 nM (for G311a); T = 25 °C. K_M and k_{cat} were determined by fitting the initial rates (V_0) at each concentration to the Michaelis-Menten model using KaleidaGraph (Synergy Software).

(B) Correlation between activities measured in cell lysate and using purified enzyme for variants selected for improved arylesterase activity. All measurements were performed at 200 μM substrate. Activities in cell lysate (enzyme co-expressed with GroEL/ES) are given relative to *wt*PTE (see SupplementaryTable S13). Kinetic data for *wt*PTE and variant H254R had been previously reported [8, 14].

*wt*PTE: T_m 78.1 ± 0.2°C

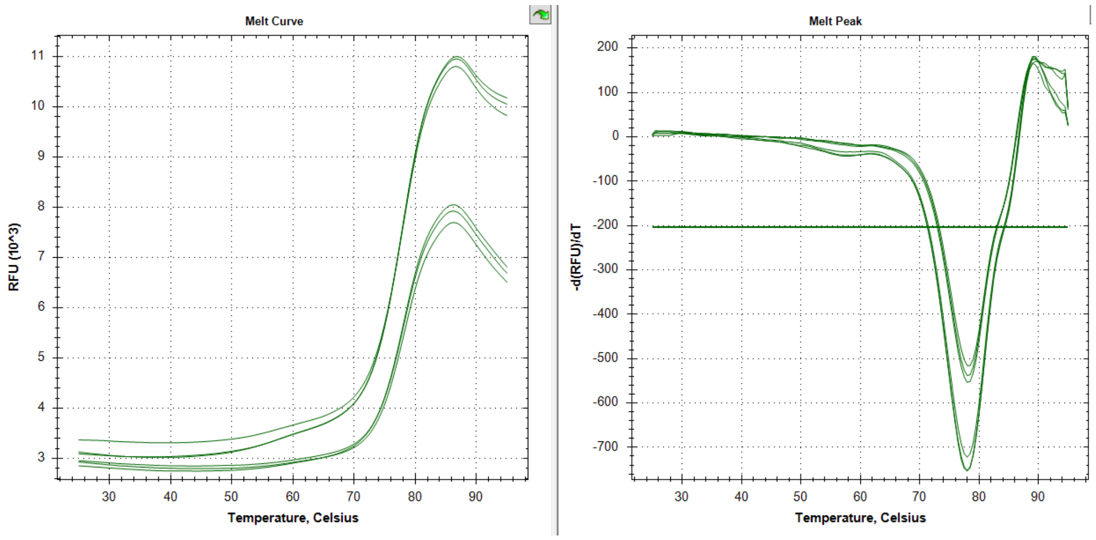

ΔA270L271L272G273: T_m 82 ± 1°C

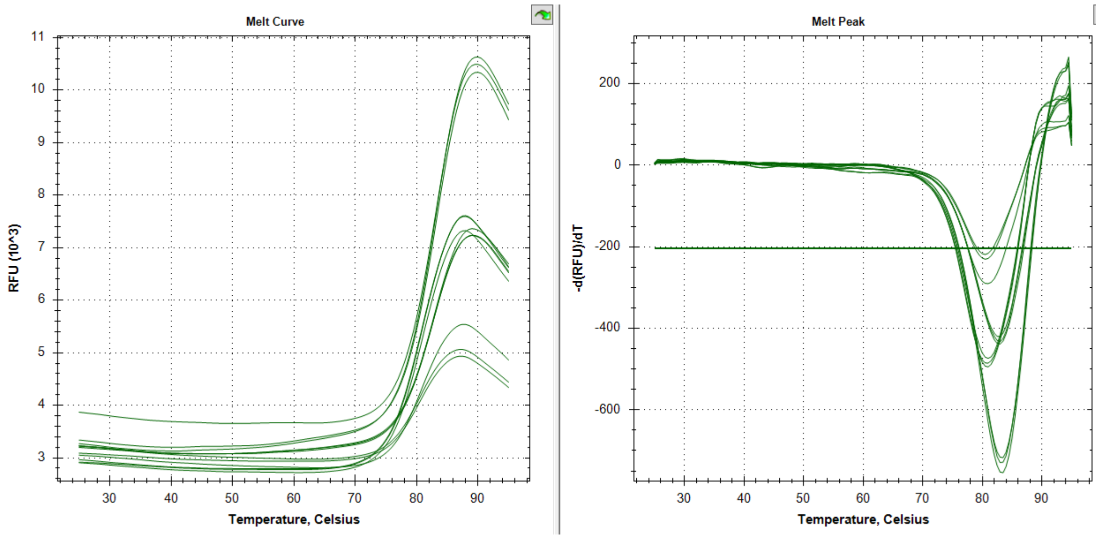

P256R/G256aA256b: T_m 84.3 ± 0.3°C

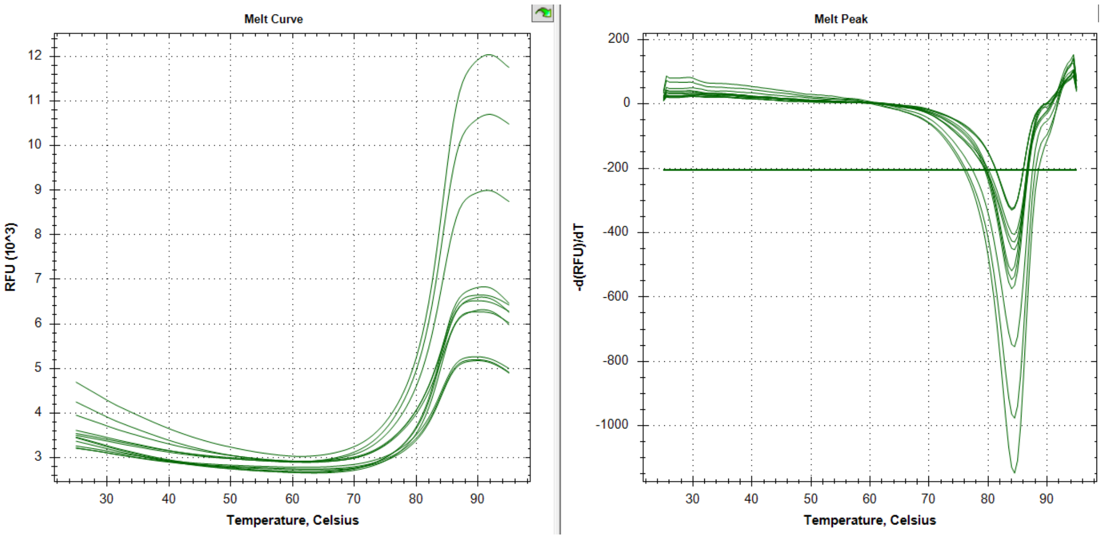

Supplementary Figure S16 (Continued on next page, legend follows).

(Figure S16 continued)

S256aG256b: T_m 77.5 ± 0.4°C & 85.6 ± 0.2°C

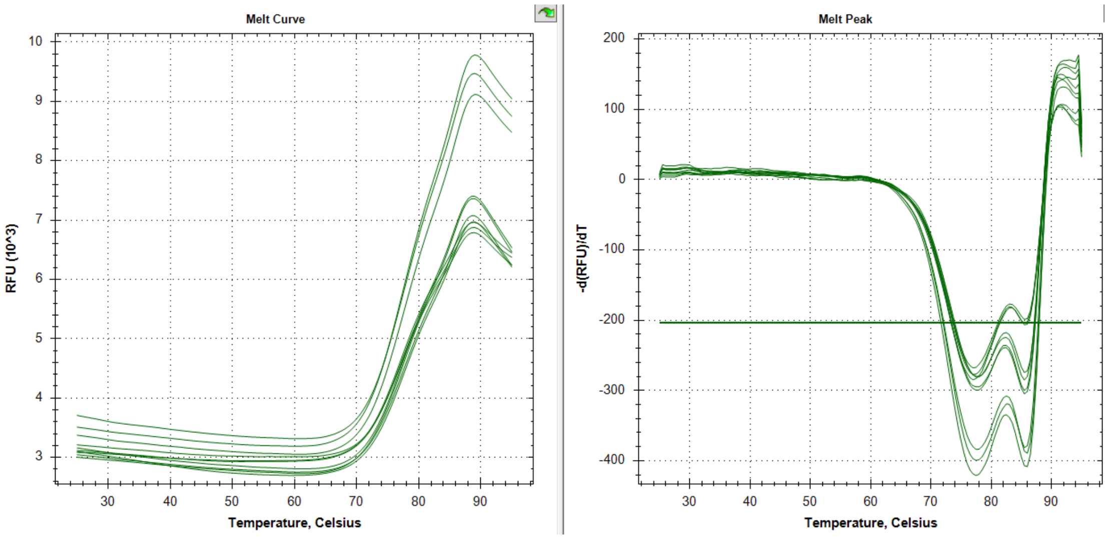

G311a: T_m 75.2 ± 0.3°C

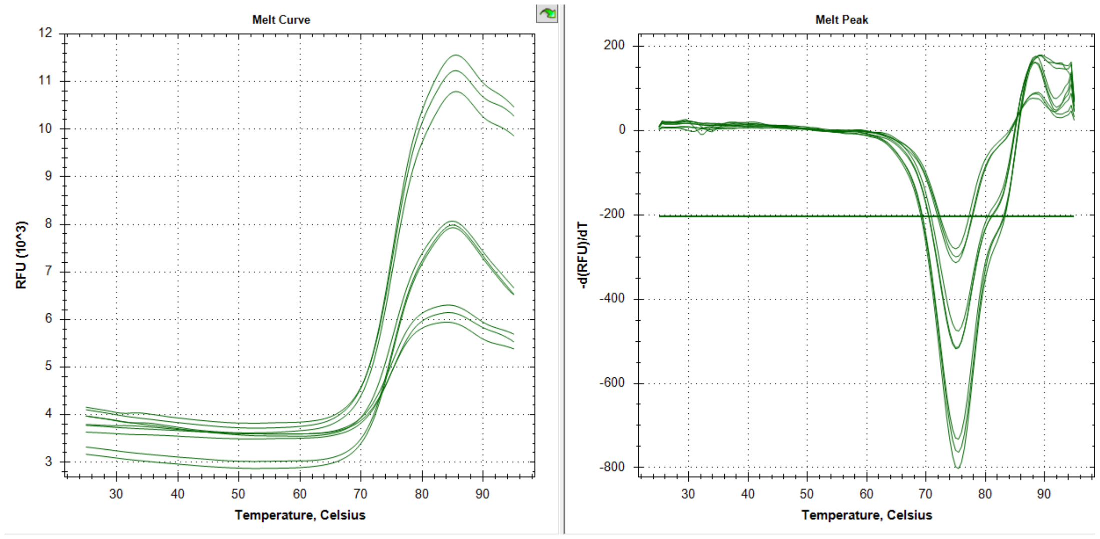

Supplementary Figure S16: T_m measurement of *wt*PTE and four selected hits

Thermostability of five purified proteins was measured using BioRad CFX Connect real-time PCR instrument by monitoring the binding of SYPRO Orange dye to unfolded protein. The sample solutions were equilibrated at 25°C for 15 minutes, then the temperature was raised to 95 °C in 0.5 °C increments with 30 s equilibration per increment. Plots on the left hand side show the fluorescence trace for each variant and right plots the first derivative with respect to time. The charts include data from all wells with a defined melting curve, which were obtained while screening multiple protein and SYPRO dye concentrations, resulting in different signal intensity between conditions. The results are given as mean ± standard deviation.

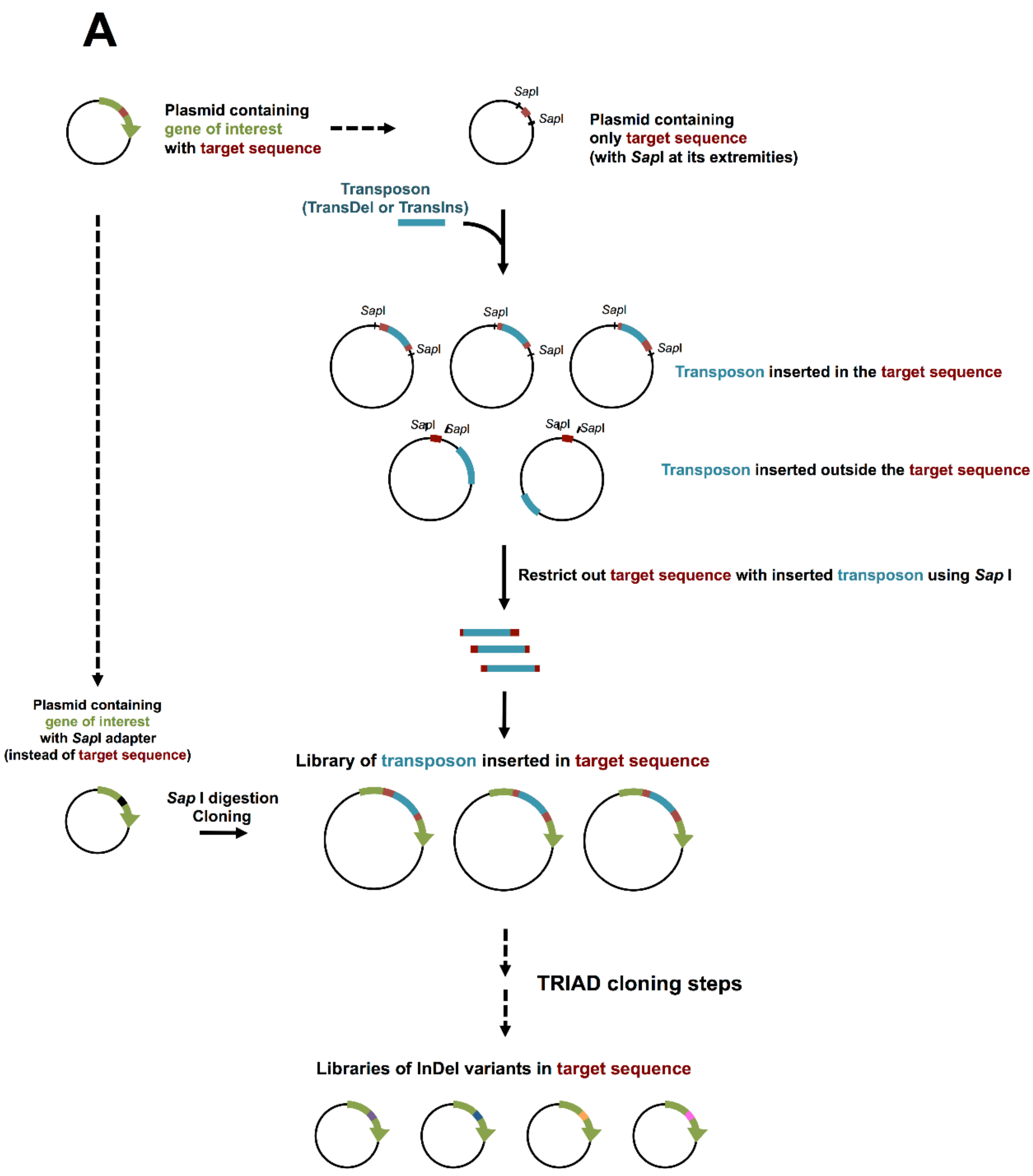

Supplementary Figure S17 (Continued on next page, legend follows).

(Figure S17 continued)

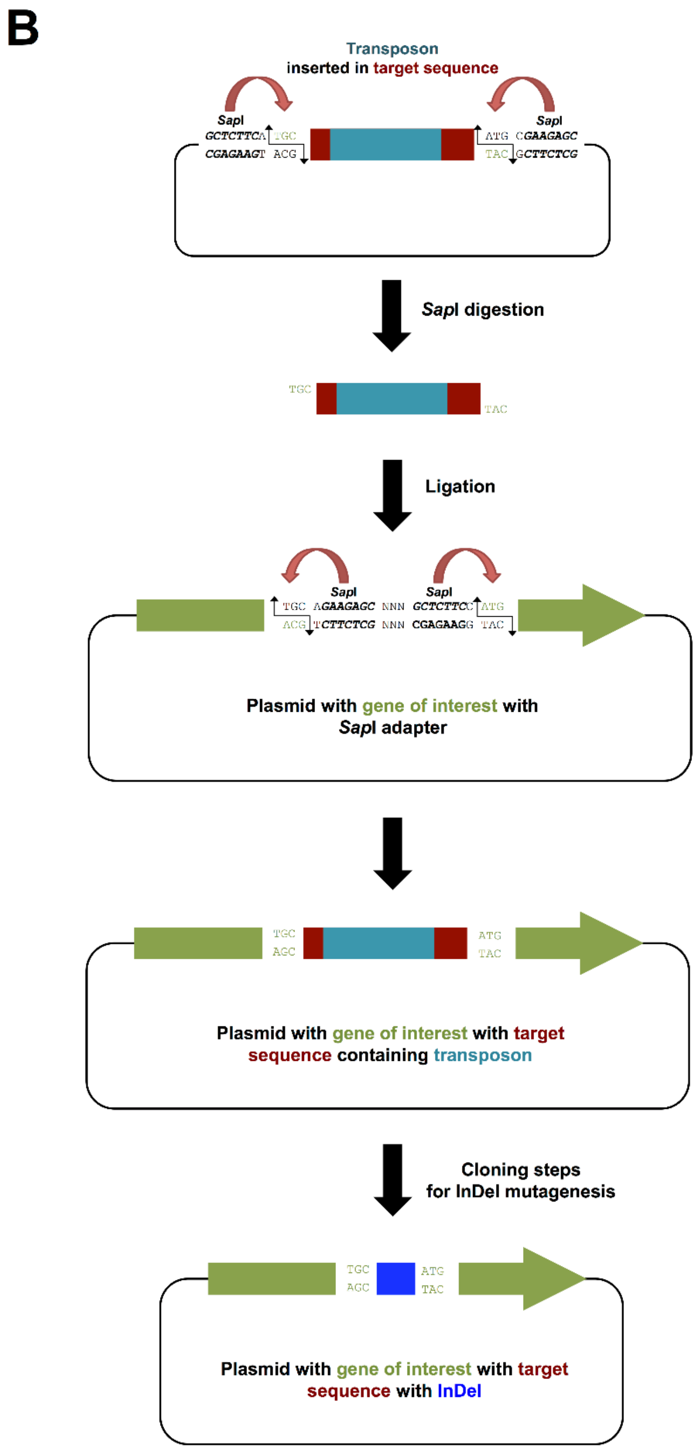

Supplementary Figure S17: Application of TRIAD for the generation of focused InDel libraries.

(A) Schematic outline of the procedure for the generation of focused InDel libraries. Transposon (*i.e.*, TransDel or TransIns) insertion is carried out on a plasmid containing only the target sequence, 'shielding' the rest of the gene from transposon integration. Target sequences are excised by SapI digestion, and those containing the inserted transposon

purified by virtue of their larger size. These fragments can subsequently be cloned into a 'SapI-adapter' plasmid, re-forming the whole gene; now containing transposon only in the target region. Finally, InDel mutagenesis is achieved following the cloning steps illustrated in Figure 1 and Figure S1.

(B) Mechanism for the cloning of focused transposon insertion libraries by seamless cloning using the type IIS restriction enzyme SapI. Seamless cloning with SapI allows cloning the target sequence without altering the original DNA sequence of the gene of interest. In the illustrated example, sequences flanking the target region (TGC and ATG) belong to the gene of interest. Upon digestion with SapI cohesive ends are generated to enable the seamless fusion of the target sequence (containing the inserted transposon) within its original gene sequence. Note that SapI is used here as an example and that any other analogous type IIS restriction enzyme (*e.g.*, BsaI or FokI) could be used to achieve this strategy.

SUPPLEMENTARY TABLES

Supplementary Table S1. Mutagenesis efficiency of TRIAD – individual variants

Libraries were generated from *wt*PTE. Upon the final transformation step, randomly chosen variants were sequenced by the Sanger method (see also Supplementary Tables S2-3). **In-frame InDels** (*i.e.*, InDels of multiple of three nucleotides) can result in adjacent substitutions (enumerated in the "*InDels with adjacent substitution*" sub-category) depending on the insertion point of the transposon. The number of *unique sequences* was recorded among the observed correct sequences. The *Unique sequences* sub-category refers to in-frame InDels that are observed at least once (see also Supplementary Table S3).

	**Deletions**				**Insertions**				
**Library**	**-3 bp**	**-6 bp**	**-9 bp**	**All deletions**	**+3 bp**	**+6 bp**	**+9 bp**	**All insertions**	**All Indels**
Total number of sequenced variants	21	21	22	64 (100%)	23	16	18	57 (100%)	121 (100%)
In-frame InDels	21	17	17	55 (86%)	11	12	12	35 (61%)	90 (74%)
InDels with no adjacent substitution	17	12	9	38 (59%)	7	10	8	25 (44%)	63 (52%)
InDels with adjacent substitution	4	5	8	17 (27%)	4	2	4	10 (18%)	27 (22%)
Unique sequences	20	17	17	54 (84%)	11	12	12	35 (61%)	89 (74%)
Frameshifting InDels	0	4	5	9 (14%)	12	4	6	22 (39%)	31 (26%)

Supplementary Table S2. Sequence analysis of naïve InDel libraries of *wt*PTE obtained with TRIAD.

Sequences were determined from randomly chosen variants upon generation of the libraries. Residues are numbered according to the crystal structure of *wt*PTE (PDB: 4PCP). The symbol Δ before a residue (or a group of residues) signifies that this (or these) residue(s) have been deleted. Inserted residues are labelled using the number of the position after which they are inserted and alphabetical order (e.g., glutamine and tyrosine residues inserted in this order after the residues at position 230 would be labelled Q230aY230b).

Library	Variant number	DNA change	Length change (bp)	Protein mutation
-3 bp	1	A(TGG)C	-3	ΔG157
	2	A(GGA)A	-3	ΔE144
	3	T(TGC)G	-3	L66C/ΔR67
	4	C(CAC)A	-3	ΔH230
	5	G(GCG)A	-3	ΔA126
	6	C(CGG)G	-3	ΔR189
	7	T(TCG)A	-3	F104Y/ΔD105
	8	G(CTA)T	-3	ΔY309
	9	A(TGA)A	-3	ΔM293
	10	G(TCT)A	-3	ΔL262
	11	A(AGA)T	-3	ΔE263
	12	C(TGG)G	-3	L272R/ΔG273
	13	G(CCG)A	-3	ΔA114
	14	C(TGA)G	-3	ΔL330
	15	C(GGG)C	-3	R280P/ΔA281
	16	C(TAC)G	-3	ΔL336
	17	T(CTA)G	-3	ΔL252
	18	G(GAG)T	-3	ΔE181
	19	C(TAC)A	-3	ΔY292
	20	C(TAC)A	-3	ΔY292
	21	A(TGG)G	-3	ΔM33
-6 bp	1	AAT(GACTGGC)TGT	-7	frameshift
	2	TCC(GAGGGCT)TGAG	-7	frameshift
	3	ATC(ATGGAC)GTG	-6	ΔM314D315
	4	CAA(TACCGT)GCG	-6	N38K/ΔT39K40
	5	TAA(CCACTC)ACA	-6	T199N/ΔT200H201
	6	AGG(CTACAT)GAA	-6	ΔY292M293
	7	CCA(CTGAGA)GTG	-6	ΔL330R331
	8	TCG(TGGCAA)ACA	-6	ΔW277Q278
	9	TCG(CGGGCT)GCC	-6	ΔR118A119
	10	CGG(TAACCACTCAC)ACG	-11	frameshift
	11	GAT(AATGCG)AGT	-6	ΔN265A266
	12	ATT(CCTACG)AGA	-6	F335L/ΔL336R337
	13	GAC(TAACCC)GGC	-6	ΔN353P354

Library	Variant number	DNA change	Length change (bp)	Protein mutation
	14	AAT(CCGAGG)GCT	-6	S218C/ΔE219G220
	15	GAG(GAGTGT)AGA	-6	ΔS142V143
	16	GTG(CGCGGT)CCT	-6	ΔR41G42
	17	TTG(GCCGGA)GTT	-6	ΔP70E71
	18	AAC(CCCGAC)GGA	-6	ΔP322D323
	19	TCC(GAGGGC)TTG	-6	ΔE219G220
	20	GGT(GTTCCGG)TAA	-7	frameshift
	21	ACC(CGGCGC)GGT	-6	P354R/ΔA355R356
-9 bp	1	CCC(TCACGGGTTT)GTA	-10	frameshift
	2	CGT(TCGTGGCAA)ACA	-9	ΔS276W277Q278
	3	CTT(CCTGCGTGA)GAT	-9	F150L-ΔL151R152E153
	4	AAA(AGGCTGTGA)GAG	-9	ΔK82A83V84
	5	AAC(ATCATGGAC)GTG	-9	ΔI313M314D315
	6	ATT(CCACTGAGA)GTG	-9	ΔP329L330R331
	7	CAG(CTCGGCAGG)ATT	-9	S61R/ΔS62A63G64
	8	GCA(AGTCAGCGC)GGT	-9	ΔS205Q206R207
	9	CGA(CCACAGGCA)AGG	-9	ΔT172T173G174
	10	ACC(GGCTTGTGG)CTC	-9	ΔG129L130W131
	11	AAG(GGCGGCCGC)CCG	-9	R185S/ΔA186A187A188
	12	G**CCC**C -> G**AATT**C	+1	frameshift
	13	TCA(TCGACCAAG)GCT	-9	I288S/ΔD289Q290G291
	14	CCT(CCATGGGCG)ATC	-9	S32Y/ΔM33G34D35
	15	CTC(TAGCGGAAA)AGG	-9	L79Q/ΔA80E81K82
	16	GTT(TCGCGGGCTG)CCG	-10	frameshift
	17	CGA(TTGGTCTAG)AAG	-9	I260K/ΔG261L262E263
	18	TCA(AGGCGCTCA)TCG	-9	K285M/ΔA286L287I288
	19	GAT(CTCGGTCGC)GAC	-9	ΔL106G107R108
	20	C(TCG)C	-3	L243P/ΔA244
	21	GCT(GCGCGCGGAT)ACC	-10	frameshift
	22	GAT(ACTGACGAT)TTG	-9	ΔT234D235D236
+3 bp	1	TT**+C GG+**G CGC	+3	L87F/G87a
	2	TT**+G TT+**A GTG	+3	L182a
	3	TCCTAT**+TT+**CACAAT	+2	frameshift
	4	CTA +CGA+ GAA	+3	R262a
	5	T**GGC**A -> T**TT**A	-1	frameshift
	6	GA**+C AT+**G GAA	+3	E144D/M144a
	7	A**C**G -> A**GGT**G	+2	frameshift
	8	TCG **+ACT+** TGG	+3	T276a
	9	ACC**+AT+**CGG	+2	frameshift
	10	TT**+A CC+**C CTG	+3	F150L/P150a
	11	C**CG**A -> C**ACGG**A	+2	frameshift
	12	AGA**+CT+**GGA	+2	frameshift
	13	TTC**+T+**CGG	+1	frameshift
	14	AA**+A TA+**T ACC	+3	N38K/Y38a

(Table S2 continued)

Library	Variant number	DNA change	Length change (bp)	Protein mutation
	15	GGT**+AA+**CTA	+2	frameshift
	16	A**TT**G -> A**ATAT**G	+2	frameshift
	17	GGT +GTT+ CTA	+3	V261a
	18	ACC **+TTC+** CAC	+3	F54a
	19	GG**+A TT+**C ATC	+3	F157a
	20	TAG**+GA+**AAG	+2	frameshift
	21	GAT **+AAT+** GAT	+3	N232a
	22	TCT**+AC+**CCG	+2	frameshift
	23	T**G**T -> T**TT**T	+1	frameshift
+6 bp	1	GG+G GGG AT+C	+6	G229aI229b
	2	TGG +ATG TTT+ CCG	+6	M69aF69b
	3	CAG +AAT CTG+ GAA	+6	N343aL343b
	4	TCC +CAG AGT+ GAG	+6	Q47aS47b
	5	A+AT GAA C+TC	+6	I44N/E44aL44b
	6	C+GG ATC C+CC	+6	R177aI177b
	7	ACC+TCGTC+GGT	+5	frameshift
	8	CG+G AAA TA+C	+6	K76aY76b
	9	CC+G ATG CC+C	+6	M178aP178b
	10	GGC +GTT AGC+ TAC	+6	V291aS291b
	11	GG+G TTA AC+C	+6	L157aT157b
	12	TGG +GGT GTA+ CCG	+6	G69aV69b
	13	see note [1]	-61	frameshift
	14	T+GG TTC G+TG	+6	L66W/F66aV66b
	15	CCA+GGTAT+CAG	+5	frameshift
	16	TTA+GTGGC+TCA	+5	frameshift
+9 bp	1	GCC+GTAAGGTT+CAG	+8	frameshift
	2	ACC +GAT TAA TGC+ CAC	+9	D54a - Stop
	3	GCT +TGT TGT CCT+ CTC	+9	C281aC281bP281c
	4	GA+A AGC TAT GA+G GAA	+9	S144aY144bE144c
	5	GTC+CCCACGTCT+CGA	+8	frameshift
	6	CTG+GGGTATGG+CGT	+8	frameshift
	7	TC+A TAT GGA AT+C	+9	Y218aG218bI218c
	8	GGT+CCTCTGGG+TTG	+8	frameshift
	9	CG+G GTG TGT CG+CA	+9	V76aC76bR76c
	10	GA+A ACT GCA AA+C	+9	D235E/T235aA235bN235c
	11	G+GA TCG TGG T+CC	+9	A90G/S90aW90bS90c
	12	CC+A GAG ACC GT+G	+9	E256aT256bV256c
	13	C+CG AGT TGA T+TG	+9	L330P/S330a - Stop
	14	CA+G GGT GGT AT+C	+9	H57Q/G57aG57bI57c
	15	TAC +ATC GTT TCG+ ATG	+9	I292aV292bS292c
	16	TCA+CCCGTCT+CGG	+7	frameshift
	17	GG+A ACA GTG CG+T	+9	T273aV273bR273c
	18	ATG+CTTTGGGG+GCA	+8	frameshift

(Table S2 continued)

[1] This variant showed a large sequence substitution where C**CATGGGCGAT-CGGATCAATACCGTGCGCGGTCCTATCACAATCTCCGAGGCGGGTTTCACACTAACC**C (modified sequence in bold) was exchanged for C**TCCAGG**C (in bold), resulting in a 61 bp deletion.

Supplementary Table S3. Frequency of in-frame InDels observed among randomly sequenced *wt*PTE variants (as recorded in Table S2).

Residues are numbered according to the crystal structure of *wt*PTE (PDB: 4PCP).

Protein position	Observed InDel	Library	Frequency	Variant name
32	S32Y/ΔM33G34D35	-9 bp	1	15
33	ΔM33	-3 bp	1	21
38	N38K/ΔT39K40	-6 bp	1	4
38	N38K/Y38a	+3 bp	1	14
41	ΔR41G42	-6 bp	1	18
44	I44N/E44aL44b	+6 bp	1	5
47	Q47aS47b	+6 bp	1	4
54	F54a	+3 bp	1	19
54	D54a - Stop	+9 bp	1	2
57	H57Q/G57aG57bI57c	+9 bp	1	16
61	S61R/ΔS62A63G64	-9 bp	1	8
66	L66C/ΔR67	-3 bp	1	3
66	L66W/F66aV66b	+6 bp	1	17
69	M69aF69b	+6 bp	1	2
69	G69aV69b	+6 bp	1	14
70	ΔP70E71	-6 bp	1	19
76	K76aY76b	+6 bp	1	9
76	V76aC76bR76c	+9 bp	1	9
79	L79Q/ΔA80E81K82	-9 bp	1	16
82	ΔK82A83V84	-9 bp	1	4
87	L87F/G87a	+3 bp	1	1
90	A90G/S90aW90bS90c	+9 bp	1	11
104	F104Y/ΔD105	-3 bp	1	7
106	ΔL106G107R108	-9 bp	1	20
114	ΔA114	-3 bp	1	13
118	ΔR118A119	-6 bp	1	9
126	ΔA126	-3 bp	1	5
129	ΔG129L130W131	-9 bp	1	11
142	ΔS142V143	-6 bp	1	17
144	ΔE144	-3 bp	1	2
144	E144D/M144a	+3 bp	1	6
144	S144aY144bE144c	+9 bp	1	4
150	F150L/ΔL151R152E153	-9 bp	1	3
150	F150L/P150a	+3 bp	1	10
157	ΔG157	-3 bp	1	1
157	F157a	+3 bp	1	20
157	L157aT157b	+6 bp	1	12
172	ΔT172T173G174	-9 bp	1	10
177	R177aI177b	+6 bp	1	7
178	M178aP178b	+6 bp	1	10

(Table S3 continued)

Protein position	Observed InDel	Library	Frequency	Variant name
181	ΔE181	-3 bp	1	18
182	L182a	+3 bp	1	2
185	R185S/ΔA186A187A188	-9 bp	1	12
189	ΔR189	-3 bp	1	6
199	T199N/ΔT200H201	-6 bp	1	5
205	ΔS205Q206R207	-9 bp	1	9
218	S218C/ΔE219G220	-6 bp	1	16
218	Y218aG218bI218c	+9 bp	1	7
219	ΔE219G220	-6 bp	1	22
229	G229aI229b	+6 bp	1	1
230	ΔH230	-3 bp	1	4
232	N232a	+3 bp	1	22
234	ΔT234D235D236	-9 bp	1	23
235	D235E/T235aA235bN235c	+9 bp	1	10
252	ΔL252	-3 bp	1	17
256	E256aT256bV256c	+9 bp	1	12
260	I260K/ΔG261L262E263	-9 bp	1	18
261	V261a	+3 bp	1	17
262	ΔL262	-3 bp	1	10
262	R262a	+3 bp	1	4
263	ΔE263	-3 bp	1	11
265	ΔN265A266	-6 bp	1	13
272	L272R/ΔG273	-3 bp	1	12
273	T273aV273bR273c	+9 bp	1	19
276	ΔS276W277Q278	-9 bp	1	2
276	T276a	+3 bp	1	8
277	ΔW277Q278	-6 bp	1	8
280	R280P/ΔA281	-3 bp	1	15
281	C281aC281bP281c	+9 bp	1	3
285	K285M/ΔA286L287I288	-9 bp	1	19
288	I288S/ΔD289Q290G291	-9 bp	1	14
291	V291aS291b	+6 bp	1	11
292	ΔY292	-3 bp	2	19, 20
292	ΔY292M293	-6 bp	1	6
292	I292aV292bS292c	+9 bp	1	17
293	ΔM293	-3 bp	1	9
309	ΔY309	-3 bp	1	8
313	ΔI313M314D315	-9 bp	1	5
314	ΔM314D315	-6 bp	1	3
322	ΔP322D323	-6 bp	1	21
329	ΔP329L330R331	-9 bp	1	6
330	ΔL330	-3 bp	1	14
330	ΔL330R331	-6 bp	1	7
330	L330P/S330a - Stop	+9 bp	1	14

(Table S3 continued)

**Protein position**	**Observed InDel**	**Library**	**Frequency**	**Variant name**
335	F335L/ΔL336R337	-6 bp	1	14
336	ΔL336	-3 bp	1	16
343	N343aL343b	+6 bp	1	3
353	ΔN353P354	-6 bp	1	15
354	P354R/ΔA355R356	-6 bp	1	24

Supplementary Table S4A: Deep sequencing coverage statistics.

All six libraries were sequenced as part of one MiSeq 2×75 bp run. Since insertion libraries have a greater theoretical diversity, they were loaded onto the flow cell at 3× the amount of deletion libraries.

**Library**	**-3 bp**	**-6 bp**	**-9 bp**
**Total reads**	1.04×10^{6}	1.09×10^{6}	8.99×10^{5}
**Assembled reads**	7.50×10^{5}	8.22×10^{5}	6.48×10^{5}
**Alignment rate**	96.4%	95.1%	90.3%
**Unassembled reads**	2.86×10^{5}	2.67×10^{5}	2.51×10^{5}
**Alignment rate**	93.5%	95.9%	88.6%
**Total aligned reads**	9.90×10^{5}	1.04×10^{6}	8.07×10^{5}
**Mean ± SD coverage per base**	$(8.59\pm1.7)\times10^{4}$	$(8.84\pm1.7)\times10^{4}$	$(6.99\pm1.6)\times10^{4}$

**Library**	**+3 bp**	**+6 bp**	**+9 bp**
**Total reads**	3.36×10^{6}	3.38×10^{6}	3.09×10^{6}
**Assembled reads**	2.56×10^{6}	2.51×10^{6}	2.47×10^{6}
**Alignment rate**	97.1%	95.0%	95.8%
**Unassembled reads**	8.08×10^{5}	8.67×10^{5}	6.28×10^{5}
**Alignment rate**	96.6%	94.0%	95.6%
**Total aligned reads**	3.27×10^{6}	3.20×10^{6}	2.968×10^{6}
**Mean ± SD coverage per base**	$(2.76\pm0.54)\times10^{5}$	$(2.73\pm0.58)\times10^{5}$	$(2.38\pm0.45)\times10^{5}$

Supplementary Table S4B: Proportion of frameshifts

The proportion of variants containing frameshifts was estimated as follows:

$$Est.\%\ frameshifted\ variants = \frac{frameshift\ reads/all\ reads}{read\ length/length\ of\ gene}$$

Where read length is equal to 2×75=150 bp and the gene length is 999 bp.

	Deletions		
Sequencing reads	-3 bp	-6 bp	-9 bp
All reads	9.90×10^5	1.04×10^6	8.07×10^5
Reads with target mutations	74923	48911	42358
% with target mutations	7.6%	4.7%	5.3%
Reads with frameshifts	6082	30813	17163
% of reads with frameshifts	0.6%	3.0%	2.1%
Est. % frameshifted variants	4.1%	19.8%	14.2%

	Insertions		
Sequencing reads	+3 bp	+6 bp	+9 bp
All reads	3.27×10^6	3.20×10^6	2.968×10^6
Reads with target mutations	121089	145374	115899
% with target mutations	3.7%	4.5%	3.9%
Reads with frameshifts	179412	139738	117505
% of reads with frameshifts	5.5%	4.4%	4.0%
Est. % frameshifted variants	36.6%	29.1%	26.4%

Supplementary Table S5: TransDel consensus site preference

(see WebLogo in Figure 3A)

Mu transposons insert within a five-nucleotide sequence, which is duplicated during the insertion. The insertion preference of TransDel in *wt*PTE gene can be calculated with precision from the location of -3 bp deletions, because these deletions are symmetrical and centred within the insertion site. The consensus site preference is built from detected -3 bp mutations, weighed according to frequency of occurrence and adjusted for orientation of transposon and GC composition.

**Position**	**A**	**C**	**G**	**T**
**1**	14.4%	34.4%	25.7%	25.5%
**2**	12.4%	38.3%	17.7%	31.7%
**3**	16.5%	33.5%	33.5%	16.5%
**4**	31.7%	17.7%	38.3%	12.4%
**5**	25.5%	25.7%	34.4%	14.4%

Supplementary Table S6: Statistics on number of reads supporting each variant

Each observed variant is associated with a count: this gives the number of reads (either an assembled paired-end 2×75 bp or a single end read from an unassembled pair) that support the detection of that variant. Some variants are observed more frequently than others. This frequency reflects the inherent bias of Mu transposon insertion, amplification bias during transformations (where one variant may randomly grow to greater abundance than another), and stochastic fluctuations resulting from sequencing. This table summarizes the *median* count in the -3 bp and +3 bp libraries, as well as maximum and interquartile range. The distribution is shown in Supplementary Figure S7 as a histogram.

**Count**	***wt*PTE -3 bp**	***wt*PTE + 3bp**
**Minimum observed**	0	0
**Q1**	4.9	11.0
**Median**	22.0	47.2
**Q3**	75.5	133.3
**Maximum**	1420	3336
**Q3-Q1**	70.6	122.3

Supplementary Table S7. Number of reads per distinct deletion observed by deep sequencing in *wt*PTE deletion libraries generated *via* TRIAD.

The histograms relative to these distributions are plotted in Supplementary Figure S7.

	Number of distinct deletions							
	**- 3 bp**		**-6 bp**		**-9 bp**		**All deletions**	
**Total number of deletions**	633		682		608		1923	
**Number of reads per deletion**								
1-4 reads	83		107		96		286	
5-9 reads	52		67		70		189	
10-39 reads	168	310 (49%)	212	369 (54.1%)	217	324 (53.3%)	597	1003 (52,2%)
40-99 reads	142		157		107		406	
100-199 reads	86		82		64		232	
200-999 reads	93	102 (16.1%)	57	57 (8.4%)	53	64 (8.9%)	203	213 (11.1%)
≥1000 reads	9		0		1		10	

Supplementary Table S8. Fitness effects in TRIAD (insertion and deletion) and trinucleotide substitution libraries of *wt*PTE.

		Deletions				Insertions				Substitutions
		**-3 bp**	**-6 bp**	**-9 bp**	**All deletions**	**+3 bp**	**+6 bp**	**+9 bp**	**All insertions**	**TriNEx**
	Number of variants[a]	175	154	156	485	92	134	125	351	342
**PARAOXONASE**	*Fitness effect*[b]*:*									
	Strongly deleterious	117 (66.9%)	143 (92.8%)	143 (91.7%)	403 (83.1%)	58 (63.2%)	106 (79.1%)	105 (84.0%)	269 (76.7%)	81 (23.8%)
	Mildly deleterious	41 (23.4%)	9 (5.9%)	11 (7.1%)	61 (12.6%)	14 (15.2%)	20 (14.9%)	13 (10.4%)	47 (13.4%)	100 (29.2%)
	Neutral	14 (8.0%)	2 (1.3%)	2 (1.3%)	18 (3.7%)	18 (19.5%)	7 (5.2%)	1 (0.8%)	26 (7.4%)	161 (47.0%)
	Beneficial	3 (1.7%)	0 (0.0%)	0 (0.0%)	3 (0.6%)	2 (2.2%)	1 (0.7%)	6 (4.8%)	9 (2.6%)	0 (0.0%)
	Average fitness change][d]	0.048	0.014	0.015	0.022	0.054	0.024	0.019	0.027	0.28
		[0.038 ; 0.062]	[0.012 ; 0.016]	[0.013 ; 0.017]	[0.02 ; 0.025]	[0.036 ; 0.082]	[0.018 ; 0.031]	[0.015 ; 0.025]	[0.023 ; 0.033]	[0.24 ; 0.33]
	Median fitness change[c]	0.03	<0.01	<0.01	<0.01	0.01	<0.01	<0.01	<0.01	0.63
	Minimum fitness change[c]	<0.01	<0.01	<0.01	<0.01	<0.01	<0.01	<0.01	<0.01	<0.01
	Maximum fitness change[c]	1.59	0.94	1.31	1.59	1.79	1.60	2.78	2.78	1.50
**ARIYLESTERASE (4-nitrophenyl butyrate)**	*Fitness effect*[b]*:*									
	Strongly deleterious	100 (57.2%)	137 (88.9%)	133 (85.2%)	370 (76.3%)	53 (57.7%)	99 (73.9%)	68 (54.3%)	220 (62.7%)	65 (19.1%)
	Mildly deleterious	40 (22.8%)	10 (6.5%)	9 (5.8%)	59 (12.2%)	16 (17.3%)	18 (13.4%)	42 (33.6%)	76 (21.6%)	96 (28.0%)
	Neutral	14 (8.0%)	6 (3.9%)	7 (4.5%)	27 (5.6%)	15 (16.3%)	8 (6.0%)	5 (4.0%)	28 (8.0%)	175 (51.1%)
	Beneficial	21 (12.0%)	1 (0.7%)	7 (4.5%)	29 (6.0%)	8 (8.7%)	9 (6.7%)	10 (8.0%)	27 (7.7%)	6 (1.8%)
	Average fitness change[c][d]	0.07	0.017	0.02	0.03	0.085	0.031	0.098	0.061	0.34
		[0.051 ; 0.095]	[0.014 ; 0.02]	[0.015 ; 0.026]	[0.025 ; 0.035]	[0.056 ; 0.13]	[0.023 ; 0.043]	[0.075 ; 0.13]	[0.05; 0.074]	[0.3 ; 0.4]
	Median fitness change[c]	0.03	<0.01	<0.01	<0.01	0.05	<0.01	0.09	0.04	0.70
	Minimum fitness change[c]	<0.01	<0.01	<0.01	<0.01	<0.01	<0.01	<0.01	<0.01	<0.01
	Maximum fitness change[c]	2.46	1.52	5.02	5.02	4.12	3.13	5.36	5.36	2.98

[a] The number of variants sampled in each library was corrected to only take in-frame mutations into account. Overall, 178 variants from each TRIAD library (6 × 178 in total) and 435 trinucleotide substitution variants were randomly picked, expressed in *E. coli* and screened for hydrolysis of paraoxon and 4-nitrophenyl butyrate (see **Table S9**). The *estimated* number of frame-shifted variants (based on the frequencies in **Table S1**) was then subtracted from the numbers of highly deleterious variants (<0.01 in both activities).

(Table S8 continued)

[b] Mutations are classified as strongly deleterious (>10-fold activity decrease relative to wtPTE), mildly deleterious (10-fold—1.5-fold decrease), neutral (<1.5-fold change), and beneficial (>1.5-fold increase).
[c] Changes in phosphotriesterase (native substrate: paraoxon) and esterase (promiscuous substrate: pNPB) activities are determined relative to those of *wt*PTE by comparing the initial rates in cell lysates measured under identical conditions with 200 µM of the respective substrates, resulting in a dimensionless ratio (see Methods).
[d] The average fitness change refers to the change in initial rates as a consequence of mutation and is calculated as the geometric mean of the relative activities of the variants for each class of mutations (see Supplementary Table S9). The corresponding confidence intervals (5% risk of error) are indicated between brackets.

Supplementary Table S9. Functional analysis of TRIAD and trinucleotide substitution libraries of *wt*PTE against paraoxon and 4-NPB

Changes in paraoxonase (native substrate: paraoxon) and arylesterase (promiscuous substrate: 4-nitrophenylbutyrate, 4-NPB) activities are determined relative to those of *wt*PTE by comparing the initial rates in cell lysates measured under identical conditions with 200 µM of the respective substrates (see Methods). Data are averages of triplicate values from three independent experiments and error values represent +/- 1 SEM.

Due to its size, the table is located in the Source Data file.

Supplementary Table S10. Analysis of solvent-accessible surface area of mutated residues in *wt*PTE variants retaining ≥50% of the parental paraoxonase activity.

The solvent accessible surface area (SASA) of residues mutated (either InDel or substitution) in *wt*PTE variants retaining ≥50% of the parental paraoxonase activity was calculated from the structure of *wt*PTE (PDB code: 4PCP) using the PISA web server at the European Bioinformatics Institute (http://www.ebi.ac.uk/pdbe/prot_int/pistart.html) [17]. Relative accessible surface area (RSA) was defined as the ratio of the SASA for a given residue within the structured protein *vs*. in the free residue [18]. Residues were classified as core for RSA < 0.25, and surface for RSA ≥ 0.25 [19].

Supplementary Table S10a. List of residues in *wt*PTE InDel variants retaining ≥50% of the parental paraoxonase activity.

Residue no.	SASA (Å^2)	RSA	Location	Corresponding variants
34	79.4	0.93	surface	F34aN34b
35	88.8	0.59	surface	∆D35R36, P35a, F35a, K35aC35b, F35aH35b, S35aC35bP35c
36	124.2	0.52	surface	∆R36, E36aY36bK36c
37	4.5	0.02	core	T37aI37b
42	24.2	0.28	surface	H42a
43	77.6	0.54	surface	P43H/A43a
45	48.6	0.33	surface	C45aP45b
49	1.6	0.01	core	∆A49
75	42.7	0.35	surface	∆S75R76K77
155	96.2	0.51	surface	∆Q155
161	48.7	0.33	surface	T161K/P161a
173	66.1	0.45	surface	∆T173
174	8.0	0.09	core	∆G174
203	34.7	0.31	surface	∆A203
205	85.0	0.70	surface	L205aG205b
206	102.0	0.54	surface	Q206H/∆R207G208
259	10.4	0.09	core	P259a
261	56.0	0.66	surface	S261a, P261a, G261aR261b, D261aT261bS261c
262	41.6	0.23	core	∆L262, I262a, P262aT262bT262c
263	126.4	0.69	surface	∆E263, E263V/Q263a
266	97.8	0.87	surface	∆A266, G266aR266b
267	31.8	0.26	surface	A266G/∆S267
269	50.3	0.41	surface	∆S269
292	56.5	0.25	core	∆Y292
293	45.9	0.22	core	∆M293, M293I/∆K294

(Table S10 continued)

Residue no.	SASA (Å^2)	RSA	Location	Corresponding variants
319	84.7	0.69	surface	S319a
337	109.0	0.45	surface	∆R337
338	126.9	0.69	surface	∆E338
339	91.4	0.43	surface	Q339a, K339M/Q339a, V339a
362	122.6	0.68	surface	∆L362, K362a
363	178.5	0.74	surface	K363aR363bR363c

Supplementary Table S10b. List of residues in wtPTE substitution variants retaining ≥50% of the parental paraoxonase activity.

Residue no.	SASA (Å^2)	RSA	Location	Corresponding variants
36	124.2	0.52	surface	R36N
44	18.0	0.10	core	I44K/T45A, I44T/T45A
45	48.6	0.33	surface	I44K/T45A, I44T/T45A
47	98.0	0.80	surface	S47Y, S47C/E48Q
48	96.6	0.53	surface	S47C/E48Q
49	1.6	0.01	core	A49M
54	9.0	0.06	core	T54S
63	41.2	0.36	surface	A63T, A63S
67	183.9	0.76	surface	R67G, R67T, R67H
73	29.3	0.13	core	F73C
75	42.7	0.35	surface	S75G
80	8.1	0.07	core	A80G, A80G/E81K
81	75.9	0.41	surface	A80G/E81K
89	124.0	0.51	surface	R89F
92	94.9	0.84	surface	A92G/A93T
93	27.5	0.24	core	A93V
96	85.8	0.36	surface	R96T
100	0.9	0.01	core	D100A
102	1.3	0.01	core	S102G, S102T
109	20.8	0.14	core	D109C, D109S
117	0.1	0.00	core	T117N/P118A
118	148.9	0.62	surface	R118K, R118G, R118Q, T117N/P118A
143	19.7	0.12	core	V143A, V143A/E144K
144	109.2	0.60	surface	E144Q, E144I
147	13.6	0.09	core	T147S
151	22.0	0.12	core	L151R
155	96.2	0.51	surface	Q155L

(Table S10 continued)

Residue no.	SASA (Å^2)	RSA	Location	Corresponding variants
162	53.2	0.63	surface	G162R, G162V
198	0.0	0.00	core	V198L
206	102.0	0.54	surface	Q206L, Q206H
208	1.1	0.01	core	G208A
223	27.3	0.19	core	P223A
235	85.3	0.57	surface	D235T
238	84.0	0.69	surface	S238G
241	25.4	0.17	core	T241I
242	53.0	0.47	surface	A242P
262	41.6	0.23	core	L262H, L262M, L262I
263	126.4	0.69	surface	E263Q
266	97.8	0.87	surface	A266D, A266G
269	50.3	0.41	surface	S269H, S269A
282	77.9	0.43	surface	L282S
292	56.5	0.25	core	Y292G, Y292R, Y292F, Y292L
293	45.9	0.22	core	M293T
302	3.1	0.01	core	W302F
308	38.6	0.32	surface	S308C
311	94.8	0.65	surface	T311A
312	97.9	0.62	surface	N312D
323	26.4	0.18	core	D323E/G324R
324	10.1	0.12	core	D323E/G324R
330	75.1	0.42	surface	L330S, L330I, L330A
331	116.1	0.48	surface	R331G
334	56.3	0.39	surface	P334N, P334T, P334A
337	109.0	0.45	surface	R337Y
339	91.4	0.43	surface	K339T, K339R
342	75.3	0.53	surface	P342L/Q343E, P342L, P342W, P342R
343	98.0	0.52	surface	Q343L, Q343G, P342L/Q343E, Q343A, Q343I, Q343W, Q343P, Q343E
348	7.9	0.09	core	G348A
351	57.4	0.36	surface	V351D
356	102.8	0.43	surface	R356E
363	178.5	0.74	surface	R363G

Supplementary Table S11. Activity and solubility of InDel (-3 and +3 bp) and substitutions (TriNucleotide Exchange; TriNEx) variants of *wt*PTE phosphotriesterase.

Upon transformation of the DNA libraries (-3 and +3 bp TRIAD libraries and TriNEx library) into *E. coli* BL21(DE3), a total of 192 colonies (64 for each library) were selected randomly, and their corresponding PTE variants sequenced to discard the ones with frameshifting mutations. Variants with non-frameshifting mutations were then screened for phosphotriesterase (PTE) activity and soluble expression (in the absence of GroEL/ES). Changes in PTE activity are determined relative to those of *wt*PTE by comparing the initial rates in cell lysates measured under identical conditions with 200 µM of the native paraoxon substrate (see Methods). Data are averages of triplicate values from three independent experiments and error values represent +/- 1 SEM. Relative soluble expression levels are determined as the ratio in protein expression levels in the clear lysate (supernatant) between variants and the parent enzyme *wt*PTE measured by SDS-PAGE (See Methods). The data are also plotted in Supplementary Figure S11.

Variant	Relative PTE activity	Relative soluble expression level	Distance to active site metal (Å)[a]	RSA[b]	Location[b] (surface/core)
**-3 bp (TRIAD)**					
ΔG261	0.11 ± 0.05	1.04	22.4	0.58	surface
ΔS62	<0.001	1.12	13.2	0.23	core
ΔL271	0.058 ± 0.006	1.44	11.5	0.28	surface
ΔA114	<0.001	0.30	18.8	0.11	core
ΔE144	<0.001	0.26	25.7	0.51	surface
ΔP334	<0.001	0.31	23.6	0.37	surface
ΔQ343	0.015 ± 0.006	0.30	27.2	0.46	surface
ΔA286	0.021 ± 0.01	0.38	23.2	0.22	core
ΔQ343	0.015 ± 0.011	0.32	27.2	0.46	surface
ΔR225	0.0041 ± 0.0004	0.65	18.3	0.05	core
ΔE181	<0.001	0.21	19.4	0.27	surface
ΔV143	0.0061 ± 0.0015	0.21	23.4	0.12	core
S258R/ΔA259	0.094 ± 0.059	1.20	14.8	0.06	core
H257R/ΔS258	<0.001	0.98	11.1	0.04	core
ΔA114	<0.001	0.10	18.8	0.11	core
ΔD235	0.17 ± 0.07	0.84	19.7	0.46	surface
ΔL346	0.0013 ± 0.0009	0.19	22.4	0.03	core
ΔL330	0.4 ± 0.17	0.90	20.0	0.39	surface
L136P/ΔS137	<0.001	0.76	22.0	0.56	surface
ΔT52	<0.001	0.33	15.1	0.01	core
ΔQ211	0.12 ± 0.04	0.36	19.6	0.27	surface
ΔL262	0.23 ± 0.08	1.13	20.9	0.22	core
A90V/ΔR91	<0.001	0.35	17.2	0.00	core
ΔH55	<0.001	0.87	6.1	0.03	core
ΔE115	<0.001	0.14	20.5	0.43	surface
F73C/ΔG74	0.034 ± 0.014	0.96	18.6	0.13	core
ΔV40	<0.001	0.23	18.2	0.02	core
ΔA355	0.0013 ± 0.001	0.32	19.5	0.15	core
ΔS102	<0.001	0.27	7.0	0.01	core

(Table S11 continued)

Variant	Relative PTE activity	Relative soluble expression level	Distance to active site metal (Å)[a]	RSA[b]	Location[b] (surface/core)
D133E/ΔP134	<0.001	0.68	13.0	0.41	surface
**+3 bp (TRIAD)**					
L251a	<0.001	0.94	10.9	0.03	core
I69a	<0.001	0.44	21.7	0.51	surface
P114a	<0.001	0.25	18.8	0.11	core
E144V/K144a	<0.001	0.29	25.7	0.51	surface
M69a	<0.001	0.38	21.7	0.51	surface
W276a	0.011 ± 0.005	1.09	19.2	0.20	core
F291a	0.049 ± 0.021	1.14	26.9	0.64	surface
L252a	0.0035 ± 0.0028	0.19	11.5	0.00	core
F322a	0.015 ± 0.005	0.85	17.8	0.67	surface
T279a	0.0097 ± 0.0051	0.87	20.0	0.30	surface
T350R/A350a	<0.001	0.14	17.8	0.08	core
V255a	0.012 ± 0.004	1.14	12.1	0.00	core
S240a	0.039 ± 0.014	0.91	19.1	0.00	core
S291a	0.21 ± 0.05	0.74	26.9	0.64	surface
P256L/T256a	0.037 ± 0.015	1.27	12.4	0.03	core
G69a	0.032 ± 0.014	1.08	21.7	0.51	surface
V114a	<0.001	0.20	18.8	0.11	core
I284N/F284a	0.013 ± 0.003	0.74	18.7	0.00	core
S260-Stop	<0.001	0.17	19.9	0.51	surface
R261a	0.4 ± 0.17	0.33	22.4	0.58	surface
F188a	<0.001	0.91	19.6	0.00	core
L261a	0.5 ± 0.15	1.00	22.4	0.58	surface
L251a	<0.001	0.84	10.9	0.03	core
V353a	<0.001	0.20	19.0	0.00	core
D271a	0.085 ± 0.052	1.07	11.5	0.28	surface
C270a	0.13 ± 0.09	0.29	14.8	0.79	surface
A63V/S63a	<0.001	0.65	15.5	0.34	surface
**Substitutions (TriNEx)**					
L262P	0.1 ± 0.04	0.83	20.9	0.22	core
R118Q	0.62 ± 0.25	0.71	22.1	0.56	surface
I250K	0.0017 ± 0.0007	0.25	14.4	0.00	core
Y292D	0.7 ± 0.11	0.74	23.4	0.22	core
I349K/T350S	<0.001	0.22	19.2	0.02	core
E115Q	0.63 ± 0.05	0.75	20.5	0.43	surface
Y292S	0.63 ± 0.16	0.72	23.4	0.22	core
H55A	0.32 ± 0.17	0.97	6.1	0.03	core
E144G	0.44 ± 0.17	0.72	25.7	0.51	surface
H55R	<0.001	0.89	6.1	0.03	core
S276C	0.55 ± 0.29	0.99	19.2	0.20	core
G348F	0.37 ± 0.1	0.67	22.6	0.08	core
R280K	0.46 ± 0.07	0.97	16.6	0.04	core
G162A	0.21 ± 0.05	0.56	23.9	0.55	surface
A165V/G166R	0.003 ± 0.0008	0.23	16.1	0.00	core
G42V	0.075 ± 0.008	0.37	22.9	0.25	core

(Table S11 continued)

Variant	Relative PTE activity	Relative soluble expression level	Distance to active site metal (Å)[a]	RSA[b]	Location[b] (surface/core)
A80L	1.2 ± 0.26	1.03	18.6	0.07	core
S62V	0.27 ± 0.06	1.08	13.2	0.23	core
G209N	0.13 ± 0.01	0.71	15.7	0.00	core
P70H/E71K	<0.001	0.40	20.9	0.24	core
G209L	0.067 ± 0.019	0.67	15.7	0.00	core
R89L	0.69 ± 0.19	1.04	19.7	0.47	surface
L262R	0.77 ± 0.1	0.94	20.9	0.22	core
V143E/E144Q	0.19 ± 0.09	0.58	23.4	0.12	core
P197R/V198L	0.0033 ± 0.0007	0.30	15.9	0.00	core
M314K/D315H	0.0045 ± 0.002	1.06	14.1	0.11	core
E159D	0.75 ± 0.06	1.20	21.5	0.42	surface
R36A	0.52 ± 0.04	0.97	24.1	0.47	surface
L262G	0.21 ± 0.03	1.11	20.9	0.22	core
S299R	<0.001	0.96	9.6	0.00	core

[a] Pairwise distances between the active site metals and all the mutated residues (alpha carbons) were calculated from the structure of *wt*PTE (PDB 4PCP) using a python script in PyMol (https://pymolwiki.org/index.php/Pairwise_distances).

[b] RSA: Relative solvent accessibility (see Supplementary Table S10). Residues were classified as core for RSA < 0.25, and surface for RSA ≥ 0.25.

Supplementary Table S12. Effects of InDels (-3 and +3 bp) and substitutions (TriNucleotide Exchange; TriNEx) on activity and soluble expression of *wt*PTE phosphotriesterase.

	Deletions (-3 bp)	Insertions (+3 bp)	Substitutions (TriNEx)
**Number of variants screened** [a]	30	27	30
Fitness (activity) effects [b]			
Beneficial	0	0	0
Neutral	0	0	5
Mildly deleterious	5	4	15
Deleterious	25	23	10
Average fitness change [c]	0.005	0.008	0.08
Minimum fitness change [b] [c]	< 0.001	< 0.001	< 0.001
Maximum fitness change [b] [c]	0.4	0.5	1.2
Effects on soluble expression [d]			
Neutral	11	13	14
Mildly destabilizing	2	3	10
Strongly destabilizing	17	11	6
Average solubility change [e]	0.45	0.54	0.68
Solubility-neutral mutations *(Variants with ≤ 25% decrease in soluble expression)*			
Number of variants	11	13	14
Average distance of mutation to active site metal (Å) [f]	16.4 ± 5.1	17.3 ± 5	16.5 ± 5.6
Median distance to active site metal	18.6	19.1	18.9
Minimum distance to active site metal	6.1	10.9	6.1
Maximum distance to active site metal	22.4	26.9	24.1
Core residues	6	7	11
Surface residues	5	6	3
Solubility-neutral and functionally deleterious mutations *(Variants with ≤ 25% decrease in soluble expression and ≥ 10-fold decrease in PTE activity)*			
Number of variants	7	12	3
Average distance of mutation to active site metal (Å) [f]	13.9 ± 4.8	16.8 ± 5	9.9 ± 3.3
T-test (p-value) [f]	0.17	0.42	0.04
Median distance to active site metal	13.9	18.4	9.6
Minimum distance to active site metal	6.1	10.9	6.1
Maximum distance to active site metal	22.0	26.9	14.1
Core residues	5	7	3
Surface residues	2	5	0

[a] See Supplementary Table S11 for screening conditions and results.

[b] Mutational effects on enzyme fitness relate to changes in phosphotriesterase (native substrate: paraoxon) activities, which are determined relative to those of *wt*PTE by comparing the initial rates in cell lysates measured under identical conditions with 200 µM of the respective substrates, resulting in a dimensionless ratio (see Methods). Mutations are classified as strongly deleterious (>10-fold activity decrease, relative to *wt*PTE), mildly deleterious (10-fold to 1.5-fold decrease), neutral (<1.5-fold change), and beneficial (>1.5-fold increase).

[c] The average fitness change refers to the change in initial rates as a consequence of mutation and is calculated as the geometric mean of the relative activities of the variants for each type of mutation (see Supplementary Table S11).

(Table S12 continued)

[d] Mutational effects on kinetic stability were inferred by the levels of soluble expression for each variant relative to that of the parent enzyme *wt*PTE. Mutations are classified as strongly destabilizing (more than 50% decrease in soluble expression, relative to *wt*PTE), mildly destabilizing (50% to 25% decrease in soluble expression), and neutral (less than 25% decrease in soluble expression).
[e] The average solubility change is calculated as the geometric mean of the relative solubility changes of the variants for each type of mutation (see Supplementary Table S11).
[f] For these variants (see Supplementary Table S11), the average distance of mutations to the active site metals is given as mean ± standard deviation. A T-test was performed to evaluate if the distribution of these distances are significantly different between solubility-neutral mutations (variants with ≤ 25% decrease in soluble expression) and solubility-neutral but functionally-deleterious mutations (variants with ≤ 25% decrease in soluble expression and ≥ 10-fold decrease in PTE activity).

Supplementary Table S13. Cell lysate activity levels of InDel variants of *wt*PTE improved in arylesterase activity.

Changes in phosphotriesterase (PTE; native substrate: paraoxon) and arylesterase (AE; promiscuous substrates: 4-NPB or 2-NH) activities are determined relative to those of *wt*PTE by comparing the initial rates in cell lysates measured under identical conditions with 200 µM of the respective substrates (see Methods). Activities were recorded after expression of wtPTE variants in the presence (+GroEL/ES) or absence (-GroEL/ES) of chaperone over-expression. Data are averages of triplicate values from three biological replicates and error values represent +/- 1 SEM. Part of this data is shown on Figures 6A and 6B which consist of AE *vs.* PTE activity plots in the presence of GroEL/ES chaperone.

Supplementary Table S13A: Promiscuous activity against 4-NPB.

		Activity relative to *wt*PTE				
		AE (4-NPB)		PTE (Paraoxon)		
Library	Protein mutation [a]	+ GroEL/ES	- GroEL/ES	+ GroEL/ES	- GroEL/ES	Relative chaperone dependency [c]
-3 bp	ΔQ206	2.3 ± 0.3	2.1 ± 0.1	0.68 ± 0.15	0.58 ± 0.04	1.1
	ΔD232	2.2 ± 0.1	2.3 ± 0.1	0.06 ± 0.01	0.05 ± 0.01	0.9
	ΔT234	2.3 ± 0.2	1.9 ± 0.2	0.41 ± 0.03	0.33 ± 0.05	1.2
	ΔH254	2.4 ± 0.2	2.3 ± 0.1	< 0.01	<0.01	1.0
	ΔS269	2.0 ± 0.1	2.0 ± 0.1	0.86 ± 0.04	0.97 ± 0.06	1.0
	L272R/ΔG273	2.2 ± 0.2	2.5 ± 0.2	0.22 ± 0.08	0.27 ± 0.12	0.9
-6 bp	ΔH257S258	5.2 ± 0.8	4.4 ± 0.3	0.06 ± 0.01	0.05 ± 0.01	1.2
	ΔS258A259	2.2 ± 0.3	1.5 ± 0.1	0.07 ± 0.02	0.06 ± 0.01	1.4
	ΔL262E263/D264H	2.1 ± 0.7	1.3 ± 0.7	0.10 ± 0.04	0.08 ± 0.02	1.7
	ΔS267A268 or ΔS269A270	2.3 ± 0.1	1.0 ± 0.2	0.25 ± 0.03	0.16 ± 0.03	2.4
	ΔA270L271	2.2 ± 0.2	1.6 ± 0.2	0.18 ± 0.02	0.16 ± 0.07	1.4
	ΔL271L272	3.0 ± 0.3	2.3 ± 0.2	0.16 ± 0.03	0.13 ± 0.02	1.3
	ΔG273I274	3.2 ± 0.3	2.3 ± 0.2	0.12 ± 0.02	0.08 ± 0.01	1.4
-9 bp	ΔG261L262E263 [b]	4.6 ± 0.6	4.0 ± 0.2	0.10 ± 0.05	0.13 ± 0.02	1.1
	ΔA268S269A270	2.6 ± 0.1	2.6 ± 0.1	0.09 ± 0.01	0.09 ± 0.01	1.0
	ΔL272G273I274	2.4 ± 0.2	2.7 ± 0.2	0.24 ± 0.02	0.28 ± 0.03	0.9

(Table S13 continued)

+3 bp	Y255a	2.3 ± 0.4	2.3 ± 0.3	< 0.01	<0.01	1.0
	L256a	14.4 ± 1.3	16.6 ± 1.2	< 0.01	<0.01	0.9
	H257a	2.9 ± 0.3	3.7 ± 0.1	0.17 ± 0.02	0.13 ± 0.01	0.8
	D258a/A259T	3.6 ± 0.7	4.4 ± 0.2	0.34 ± 0.06	0.29 ± 0.03	0.8
	V258a/A259P	3.7 ± 0.1	3.1 ± 0.5	0.79 ± 0.26	0.70 ± 0.04	1.2
	I258a	2.8 ± 0.8	2.3 ± 0.3	0.49 ± 0.13	0.41 ± 0.06	1.2
	P259a	3.4 ± 0.2	3.9 ± 0.3	0.95 ± 0.06	1.12 ± 0.09	0.9
	A259G/S259a	4.1 ± 0.2	3.8 ± 0.2	0.37 ± 0.03	0.30 ± 0.04	1.1
	S261a	2.1 ± 0.2	1.8 ± 0.2	0.78 ± 0.01	0.76 ± 0.02	1.2
	P261a	2.7 ± 0.1	2.7 ± 0.1	0.95 ± 0.01	0.94 ± 0.01	1.0
	W261a	3.1 ± 0.4	1.0 ± 0.2	0.11 ± 0.02	0.05 ± 0.01	3.1
	S319a	2.5 ± 0.1	2.6 ± 0.1	1.24 ± 0.03	1.30 ± 0.04	1.0
+6 bp	G255aA255b	9.4 ± 0.9	8.1 ± 0.5	0.05 ± 0.01	0.03 ± 0.01	1.2
	D258aR258b/A259S	4.0 ± 0.1	3.2 ± 0.2	0.21 ± 0.01	0.16 ± 0.02	1.2
	P261aC261b	2.6 ± 0.1	2.6 ± 0.1	0.21 ± 0.01	0.22 ± 0.01	1.0
	I261aG261b	3.3 ± 0.1	1.9 ± 0.1	1.39 ± 0.11	0.64 ± 0.04	1.7
	E261aS261b	4.0 ± 0.2	2.8 ± 0.2	0.70 ± 0.05	0.41 ± 0.03	1.4
	V262aL262b	4.8 ± 0.2	1.9 ± 0.1	1.25 ± 0.03	0.48 ± 0.02	2.5
	W262aK262b	4.3 ± 0.1	2.9 ± 0.2	0.46 ± 0.04	0.26 ± 0.04	1.5
	L265aP265b	2.8 ± 0.2	1.9 ± 0.1	1.03 ± 0.06	0.62 ± 0.05	1.4
	G265aY265b/A266S	2.9 ± 0.1	2.1 ± 0.2	1.24 ± 0.07	0.70 ± 0.05	1.4
	G266aR266b	2.6 ± 0.1	2.4 ± 0.1	0.79 ± 0.10	0.50 ± 0.15	1.1
	Y268aR268b	2.9 ± 0.2	2.5 ± 0.3	0.16 ± 0.01	0.14 ± 0.01	1.2
	Q271aR271b	3.0 ± 0.2	3.4 ± 0.2	0.13 ± 0.02	0.16 ± 0.02	0.9
	R271aC271b	2.8 ± 0.1	1.9 ± 0.4	0.12 ± 0.01	0.05 ± 0.01	1.4
	Y275aG275b	3.1 ± 0.2	2.7 ± 0.3	0.09 ± 0.01	0.07 ± 0.01	1.2
+9 bp	T121aS121bD121c	2.8 ± 0.7	1.2 ± 0.1	0.21 ± 0.06	0.06 ± 0.01	2.3

(Table S13 continued)

K257aH257bG257c	4.5 ± 0.3	5.3 ± 0.4	0.17 ± 0.02	0.21 ± 0.03	0.9
S258aG258bF258c	2.3 ± 0.3	2.0 ± 0.2	0.25 ± 0.02	0.18 ± 0.02	1.2
C261aK261bL261c	5.4 ± 0.3	6.2 ± 0.5	0.22 ± 0.01	0.23 ± 0.01	0.9
D261aT261bS261c	3.3 ± 0.1	3.7 ± 1.1	1.18 ± 0.10	1.48 ± 0.15	0.9
D261aW261bK261c	2.9 ± 0.1	2.7 ± 0.3	0.43 ± 0.01	0.44 ± 0.01	1.1
H261aI261bL261c	4.9 ± 0.2	2.3 ± 0.7	0.22 ± 0.01	0.08 ± 0.01	2.1
V261aN261bG261c	2.6 ± 0.1	1.2 ± 0.1	0.18 ± 0.01	0.09 ± 0.01	2.1
G262aL262bE262c/E263K	2.3 ± 0.4	2.5 ± 0.2	1.00 ± 0.15	0.95 ± 0.13	0.9
L268aG268bC268c/S269P	2.1 ± 0.1	1.4 ± 0.1	0.13 ± 0.01	0.05 ± 0.01	1.6
S269aG269bS269c	3.0 ± 0.1	2.8 ± 0.1	0.41 ± 0.05	0.38 ± 0.02	1.1
T269aS269bG269c	2.5 ± 0.2	2.0 ± 0.4	0.35 ± 0.04	0.25 ± 0.05	1.3
E276aG276bM276c	2.7 ± 0.3	2.5 ± 0.1	0.12 ± 0.02	0.10 ± 0.02	1.0
A309aA309bA309c	2.1 ± 0.1	1.8 ± 0.2	0.28 ± 0.02	0.24 ± 0.02	1.1

Supplementary Table S13B: Promiscuous activity against 2-NH.

		Activity relative to *wt*PTE				
		AE (2-NH)		PTE (Paraoxon)		
Library	Protein mutation [a]	+ GroEL/ES	- GroEL/ES	+ GroEL/ES	- GroEL/ES	Relative chaperone dependency[c]
-3 bp	ΔG273	3.1 ± 0.3	2.2 ± 0.3	0.20 ± 0.01	0.15 ± 0.02	1.4
	ΔL303	3.3 ± 0.5	5.6 ± 0.2	< 0.01	< 0.01	0.6
	ΔS308	8.7 ± 1.2	2.8 ± 0.1	0.41 ± 0.04	0.15 ± 0.01	3.1
	ΔT311	6.3 ± 0.6	3.2 ± 0.3	0.48 ± 0.04	0.31 ± 0.02	1.9
	ΔV316	4.1 ± 0.6	3.9 ± 0.2	0.04 ± 0.01	< 0.01	1.1
	ΔM317	9.5 ± 0.7	5.3 ± 0.6	< 0.01	< 0.01	1.8
-6 bp	ΔI260G261	2.6 ± 0.1	1.7 ± 0.1	0.02 ± 0.01	< 0.01	1.5

(Table S13 continued)

	I313N/ΔM314D315	5.2 ± 0.2	4.4 ± 0.2	< 0.01	< 0.01	1.2
-9 bp	ΔP256H257S258	8.7 ± 0.5	4.9 ± 0.3	< 0.01	< 0.01	1.8
	ΔG261L262E263 [b]	3.6 ± 0.3	1.6 ± 0.1	0.29 ± 0.01	0.23 ± 0.01	2.3
	ΔA270L271L272G273	10.5 ± 2.0	2.6 ± 0.2	0.58 ± 0.08	0.22 ± 0.03	4.1
+3 bp	A310a	8.1 ± 0.2	5.1 ± 0.3	0.93 ± 0.06	0.79 ± 0.05	1.6
	G311a	15.7 ± 3.2	14.4 ± 3.6	0.28 ± 0.04	0.36 ± 0.10	1.1
	P311a	5.7 ± 0.3	4.6 ± 0.1	0.24 ± 0.02	0.21 ± 0.02	1.2
	I313R/F313a	14.3 ± 0.5	9.7 ± 0.6	0.11 ± 0.03	0.03 ± 0.01	1.5
	I313M/F313a	5.0 ± 0.3	3.7 ± 0.3	< 0.01	< 0.01	1.4
+6 bp	V99G/Q99aI99b	6.5 ± 0.6	5.0 ± 0.4	0.46 ± 0.04	0.30 ± 0.03	1.3
	P256R/G256aA256b	138.6 ± 11.4	102.9 ± 4.7	< 0.01	< 0.01	1.3
	S256aG256b	28.0 ± 1.3	29.6 ± 2.3	< 0.01	< 0.01	0.9
	V256aW256b	9.0 ± 1.8	8.8 ± 1.1	0.07 ± 0.02	0.03 ± 0.01	1.0
	H257Q/T257aY257b	4.9 ± 1.0	2.1 ± 0.2	1.52 ± 0.18	0.85 ± 0.08	2.4
	I313K/V313aV313b	10.6 ± 1.4	12.9 ± 2.1	0.04 ± 0.01	0.12 ± 0.08	0.8
	I313S/S313aL313b	10.0 ± 1.1	7.4 ± 0.6	0.02 ± 0.01	0.02 ± 0.01	1.3
+9 bp	V310A/S310aD310bI310c	5.2 ± 0.6	8.1 ± 0.4	0.31 ± 0.03	0.37 ± 0.02	0.6
	T311K/P311aE311bA311c	3.9 ± 0.1	0.6 ± 0.1	< 0.01	< 0.01	6.6
	T311S/M311aV311bS311c	3.0 ± 0.2	1.9 ± 0.3	0.36 ± 0.03	0.16 ± 0.05	1.6

[a] The symbol Δ before a residue (or a group of residues) signifies that this (or these) residue(s) have been deleted. Inserted residues are labelled using the number of the position after which they are inserted and alphabetical order (e.g., glutamine and tyrosine residues inserted in this order after the residues at position 230 would be labelled Q230aY230b).

[b] This variant (ΔG261L262E263) was found in both screening campaign against 4-NPB and 2-NH.

[c] Relative chaperone dependency refers to the ratio of relative AE activities in the presence *vs.* absence of chaperone over-expression.

Supplementary Table S14. Change in soluble expression of PTE InDel variants improved in arylesterase activity in the presence or absence of chaperone co-expression.

The soluble expression of each PTE variant was analyzed by SDS-PAGE (shown in Supplementary Figure S14). PTE appears as a minor and major band in the insoluble fraction, and the major band only in the soluble fraction. The intensity of both bands combined was quantified using ImageJ and normalized to background intensity. The percentage of soluble expression is given by: soluble band intensity / (soluble band intensity + pellet band intensity).

Soluble expression	Without GroEL/ES	With GroEL/ES
***wt*PTE**	63%	77%
**ΔA270L271L272G273**	90%	81%
**P256R/G256aA256b**	56%	59%
**S256aG256b**	59%	70%
**G311a**	64%	83%

Supplementary Table S15. Sequence analysis of naïve TRIAD libraries focused on Loop 7 of *wt*PTE.

Sequences were determined from randomly chosen variants upon generation of the libraries. Residues are numbered according to the crystal structure of *wt*PTE (PDB: 4PCP). *Occurrence* refers to the number of times that a specific mutation was observed among the sequenced variants.

Library	Variant number	DNA mutation	Length change (bp)	Protein mutation	Occurrence
-3 bp	1	GGT(CTAG)AAG	-4 bp	frameshift	n.a.
	2	C(TGG)GT	-3 bp	L272R/ΔG273	8
	3	AG(TGCG)A	-4 bp	frameshift	n.a.
	4	TC(AGC)C	-3 bp	ΔA270	7
	5	CT(GGGT)A	-4 bp	frameshift	n.a.
	6	GA(CCA)T	-3 bp	ΔH254	3
	7	C(TGG)GT	-3 bp	L272R/ΔG273	8
	8	GG(TCT)A	-3 bp	ΔL262	9
	9	ATT(CCG)CAC	-3 bp	ΔP256	4
	10	CT(AGA)C	-3 bp	ΔD253	2
	11	GA(CCA)T	-3 bp	ΔH254	3
	12	ATT(CCG)CAC	-3 bp	ΔP256	4
	13	A(TTG)GT	-3 bp	I260S/ΔG261	2
	14	C(TGG)GT	-3 bp	L272R/ΔG273	8
	15	CGT(TCG)TGG	-3 bp	ΔS276	3
	16	C(TGG)GT	-3 bp	L272R/ΔG273	8
	17	A(TTG)GT	-3 bp	I260S/ΔG261	2
	18	TC(AGC)C	-3 bp	ΔA270	7
	19	TC(AGC)C	-3 bp	ΔA270	7
	20	GGT(CTA)GAA	-3 bp	ΔL262	9
	21	ATT(GGT)CTA	-3 bp	ΔG261	1
	22	CT(CCT)G	-3 bp	ΔL271/272	1
	23	GGT(CTA)GAA	-3 bp	ΔL262	9
	24	CAC(AGT)GCG	-3 bp	ΔS258	2
	25	GG(TCT)A	-3 bp	ΔL262	9
	26	TCG(TGG)CAA	-3 bp	ΔW277	2
	27	TCA(GCC)CTC	-3 bp	ΔA270	7
	28	TC(AGC)C	-3 bp	ΔA270	7
	29	CTA(GAA)GAT	-3 bp	ΔE263	2
	30	CAC(AGT)GCG	-3 bp	ΔS258	2
	31	GCA(TCAG)CCC	-4 bp	frameshift	n.a.
	32	ATT(CCG)CAC	-3 bp	ΔP256	4
	33	C(TGG)GT	-3 bp	L272R/ΔG273	8
	34	CTA(GAA)GAT	-3 bp	ΔE263	2
	35	T(CGT)GG	-3 bp	ΔS276	3
	36	G(GTA)TT	-3 bp	G273V/ΔI274	1
	37	TC(AGC)C	-3 bp	ΔA270	7
	38	C(TGG)GT	-3 bp	L272R/ΔG273	8

(Table S15 continued)

Library	Variant number	DNA mutation	Length change (bp)	Protein mutation	Occurrence
	39	TCG(TGG)CAA	-3 bp	ΔW277	2
	40	AG(TGCG)A	-4 bp	Frameshift	n.a.
	41	C(TAG)AA	-3 bp	L262Q/ΔE263	2
	42	CGT(TCG)TGG	-3 bp	ΔS276	3
	43	CTA(GAC)CAT	-3 bp	ΔD253	2
	44	AT(TCCG)C	-4 bp	frameshift	n.a.
	45	GG(TCT)A	-3 bp	ΔL262	9
	46	C(TAG)AA	-3 bp	L262Q/ΔE263	2
	47	TCA(GCC)CTC	-3 bp	ΔA270	7
	48	C(TGG)GT	-3 bp	L272R/ΔG273	8
	49	GG(TCT)A	-3 bp	ΔL262	9
	50	GGT(CTAG)AAG	-4 bp	frameshift	n.a.
	51	GG(TCT)A	-3 bp	ΔL262	9
	52	AAT(GCG)AGT	-3 bp	ΔA266	1
	53	C(TGG)GT	-3 bp	L272R/ΔG273	8
	54	GG(TCT)A	-3 bp	ΔL262	9
	55	ATT(CCG)CAC	-3 bp	ΔP256	4
	56	GA(CCA)T	-3 bp	ΔH254	3
	57	AG(TGCG)A	-4 bp	frameshift	n.a.
	58	GG(TCT)A	-3 bp	ΔL262	9
	59	GCC(CTCC)TGG	-4 bp	frameshift	n.a.
-6 bp	1	CTC(CTGGGT)ATT	-6 bp	ΔL272G273	2
	2	GC(GAGTGC)ATCA	-6 bp	ΔS267A268	4
	3	ATC(GGTCTA)GAC	-6 bp	ΔG251L252	2
	4	G(GTCTAG)AA	-6 bp	ΔG261L262	5
	5	CT(CCTGGGT)ATTC	-7 bp	frameshift	n.a.
	6	ATC(GGTCTAGA)CCA	-8 bp	frameshift	n.a.
	7	GG(TCTAGA)AGAT	-6 bp	ΔL262E263	8
	8	T(CGTGGC)AA	-6 bp	S276stop	1
	9	ATT(CGTTCG)TGG	-6 bp	ΔR275S276	1
	10	AT(TGGTCT)A	-6 bp	ΔG261L262	5
	11	GG(TCTAGA)A	-6 bp	ΔL262E263	8
	12	AT(TGGTCTAG)A	-8 bp	frameshift	n.a.
	13	AT(TGGTCT)A	-6 bp	ΔG261L262	5
	14	GC(GAGTGC)A	-6 bp	ΔS267A268	4
	15	CGT(TCGTGG)CAA	-6 bp	ΔS276W277	4
	16	CTC(CTGGGTA)TTC	-7 bp	frameshift	n.a.
	17	GGT(CTAGAA)GAT	-6 bp	ΔL262E263	8
	18	CT(GGGTAT)T	-6 bp	ΔG273I274	3
	19	GG(TCTAGA)A	-6 bp	ΔL262E263	8
	20	C(TAGAAG)AT	-6 bp	L262H/ΔE263D264	6
	21	C(TCCTGG)GT	-6 bp	L271R/ΔL272G273	1
	22	CT(GGGTAT)T	-6 bp	ΔG273I274	3
	23	GG(TCTAGA)A	-6 bp	ΔL262E263	8
	24	GG(TCTAGA)A	-6 bp	ΔL262E263	8

(Table S15 continued)

Library	Variant number	DNA mutation	Length change (bp)	Protein mutation	Occurrence
	25	ATT(CCGCAC)AGT	-6 bp	ΔP256H257	7
	26	AT(CGGTCTA)G	-7 bp	frameshift	n.a.
	27	AT(TGGTCT)A	-6 bp	ΔG261L262	5
	28	C(TAGAAG)AT	-6 bp	L262H/ΔE263D264	6
	29	TCG(TGGCAA)ACA	-6 bp	ΔW276Q277	1
	30	C(TAGAAG)AT	-6 bp	L262H/ΔE263D264	6
	31	CTC(ATCGGTC)TAG	-7 bp	frameshift	n.a.
	32	C(ACAGTG)CG	-6 bp	H257P/ΔS258A259	2
	33	AT(TGGTCT)A	-6 bp	ΔG261L262	5
	34	CTC(CTGGGTA)TTC	-7 bp	frameshift	n.a.
	35	AT(TCCGCA)C	-6 bp	ΔP256H257	7
	36	CTC(CTGGGT)ATT	-6 bp	ΔL272G273	2
	37	ATT(CCGCAC)AGT	-6 bp	ΔP256H257	7
	38	ATT(GGTCTAGA)AG	-7 bp	frameshift	n.a.
	39	T(CAGCCC)TC	-6 bp	S269F/ΔA270L271	2
	40	C(ACAGTG)CG	-6 bp	H257P/ΔS258A259	2
	41	GG(TCTAGA)A	-6 bp	ΔL262E263	8
	42	CGT(TCGTGGC)AAA	-7 bp	frameshift	n.a.
	43	CC(GCACAG)T	-6 bp	ΔH257S258	1
	44	CGT(TCGTGG)CAA	-6 bp	ΔS276W277	4
	45	GC(GAGTGC)A	-6 bp	ΔS267A268	4
	46	TC(AGCCCTC)C	-7 bp	frameshift	n.a.
	47	GGT(CTAGAA)GAT	-6 bp	ΔL262E263	8
	48	GG(TCTAGA)A	-6 bp	ΔL262E264	1
	49	CTA(GACCATA)TTC	-7 bp	frameshift	n.a.
	50	AT(TCCGCA)C	-6 bp	ΔP256H257	7
	51	CGT(TCGTGG)CAA	-6 bp	ΔS276W277	4
	52	GCC(CTCCTG)GGT	-6 bp	ΔL271L272	2
	53	ATT(CCGCAC)AGT	-6 bp	ΔP256H257	7
	54	C(TAGAAG)AT	-6 bp	L262H/ΔE263D264	6
	55	ATT(CCGCAC)AGT	-6 bp	ΔP256H257	7
	56	CAT(ATTCCG)CAC	-6 bp	ΔI255P256	3
	57	CT(AGACCA)T	-6 bp	ΔD253H254	2
	58	CAT(ATTCCG)CAC	-6 bp	ΔI255P256	3
	59	C(TAGAAG)AT	-6 bp	L262H/ΔE263D264	6
	60	ATT(CCGCAC)AGT	-6 bp	ΔP256H257	7
	61	C(TAGAAG)AT	-6 bp	L262H/ΔE263D264	6
	62	GC(CCTCCT)G	-6 bp	ΔL271L272	2
	63	AA(TGCGAGT)G	-7 bp	frameshift	n.a.
	64	CTA(GACCAT)ATT	-6 bp	ΔD253H254	2
	65	GC(GAGTGC)A	-6 bp	ΔS267A268	4
	66	CTC(CTGGGTA)TTC	-7 bp	frameshift	n.a.
	67	C(ACAGTGC)GA	-7 bp	frameshift	n.a.
	68	T(CAGCCC)TC	-6 bp	S269F/ΔA270L271	2
	69	CT(GGGTAT)T	-6 bp	ΔG273I274	3

(Table S15 continued)

Library	Variant number	DNA mutation	Length change (bp)	Protein mutation	Occurrence
	70	G(ACCATA)TT	-6 bp	D253V/ΔH254I255	1
	71	GC(ATCAGC)C	-6 bp	ΔS269A270	1
	72	CAT(ATTCCG)CAC	-6 bp	ΔI255P256	3
	73	CGT(TCGTGG)CAA	-6 bp	ΔS276W277	4
	74	AT(CGGTCT)A	-6 bp	ΔG251L252	2
	75	G(CCCTCC)TG	-6 bp	A270V/ΔL271L272	1
	76	GGT(CTAGACC)ATA	-7 bp	frameshift	n.a.
	77	TC(AGCCCT)C	-6 bp	ΔA270L271	1
+3 bp	1	GGT+A+CT	+1 bp	frameshift	n.a.
	2	CA+AAA+T	+3 bp	H254Q/N254a	1
	3	C+GGT+TA	+3 bp	R261a	1
	4	GGT+TTT+CTA	+3 bp	F251a	1
	5	C+ATT+TG	+3 bp	H271a	2
	6	G+GCT+AA	+3 bp	E263G/Stop	1
	7	CTC+AT+C	+2 bp	frameshift	n.a.
	8	CA+CCA+A	+3 bp	H277a	1
	9	CTA+CTA+GAA	+3 bp	L262a	4
	10	C+GGC+AC	+3 bp	R256a	1
	11	ATT+ATG+CCG	+3 bp	M255a	1
	12	T+TG+CGT	+2 bp	frameshift	n.a.
	13	GA+GCT+C	+3 bp	D253E/L253a	1
	14	CT+TGT+A	+3 bp	V262a	1
	15	GGT+GTT+CTA	+3 bp	V261a	1
	16	GA+GA+CC	+2 bp	frameshift	n.a.
	17	GGTC+A+TAGA	+1 bp	frameshift	n.a.
	18	GA+ATA+CCAT	+3 bp	D253E/Y253a	1
	19	G+AT+CGA	+2 bp	frameshift	n.a.
	20	ATT+AGT+GGT	+3 bp	S260a	1
	21	GG+GTC+T	+3 bp	S261a	1
	22	CT+GTG+C	+3 bp	C271a	1
	23	GGT+TA+CTAG	+2 bp	frameshift	n.a.
	24	G+AGG+CG	+3 bp	E265a	1
	25	CTA+T+GA	+1 bp	frameshift	n.a.
	26	AT+GAT+T	+3 bp	M259a	1
	27	C+TA+CGC	+2 bp	frameshift	n.a.
	28	CTC+CAT+CTG	+3 bp	H271a	2
	29	GGT+TG+CTAG	+2 bp	frameshift	n.a.
	30	CT+CTT+C	+3 bp	F271a	3
	31	ATT+TCC+CCG	+3 bp	S255a	1
	32	C+ATC+TA	+3 bp	H261a	1
	33	CT+CTT+G	+3 bp	L272a	1
	34	CT/A+G+AGA	+1 bp	frameshift	n.a.
	35	T+GTT+CA	+3 bp	C268a	1
	36	GGT+TAG+CTA	+3 bp	Stop	2
	37	CCGC+CGC+AC	+3 bp	P256a	1

(Table S15 continued)

Library	Variant number	DNA mutation	Length change (bp)	Protein mutation	Occurrence
	38	AA+AG+TG	+2 bp	frameshift	n.a.
	39	CGTT+GAT+CG	+3 bp	Stop	2
	40	CTC+TTT+CTG	+3 bp	F271a	3
	41	CGT+A+TC	+1 bp	frameshift	n.a.
	42	CT+TCT+A	+3 bp	L262a	4
	43	C+GT+CGC	+2 bp	frameshift	n.a.
	44	CTA+TCA+GAC	+3 bp	S252a	1
	45	C+AGT+TG	+3 bp	Q271a	1
	46	GG+CAT+T	+3 bp	I261a	1
	47	TCA+TCA+GCC	+3 bp	S269a	1
	48	ATC+AT+GGTC	+2 bp	frameshift	n.a.
	49	CT+TCT+A	+3 bp	L262a	4
	50	GT+AC+CTAG	+2 bp	frameshift	n.a.
	51	GGT+GAA+CTA	+3 bp	E261a	1
	52	A+ATA+CA	+3 bp	N278a	1
	53	ATT+CTT+CCG	+3 bp	L255A	1
	54	CT+TAG+G	+3 bp	R272a	1
	55	GGT+GCT+CTA	+3 bp	A261a	2
	56	GGT+A+CT	+1 bp	frameshift	n.a.
	57	CC+C+GCA	+1 bp	frameshift	n.a.
	58	CT+CG+AG	+2 bp	frameshift	n.a.
	59	CT+TGT+A	+3 bp	V252a	1
	60	ATT+ATT+CCG	+3 bp	I255a	1
	61	CT+TCA+A	+3 bp	Q262a	1
	62	CT+TC+GG	+2 bp	frameshift	n.a.
	63	CT+TTT+A	+3 bp	L262a	4
	64	C+CTC+TA	+3 bp	P251a	1
	65	GAC+TC+C	+2 bp	frameshift	n.a.
	66	GGT+GG+CTAG	+2 bp	frameshift	n.a.
	67	GGTC+CAT+TA	+3 bp	P261a	2
	68	GCGA+CGA+TT	+3 bp	T259a	1
	69	CTC+TTT+CTG	+3 bp	F271a	3
	70	CCG+TC+C	+2 bp	frameshift	n.a.
	71	GCGAT+GAG+T	+3 bp	I260M/S260a	1
	72	CTC+CCC+CTG	+3 bp	P271a	1
	73	GG+CTA+T	+3 bp	Y261a	1
	74	GGT+GCT+CTA	+3 bp	A261a	2
	75	T+CT+GGC	+2 bp	frameshift	n.a.
	76	GGT+CCT+GTA	+3 bp	P261a	2
	77	GGTC+CA+TAG	+2 bp	frameshift	n.a.

Supplementary Table S16. Methods developed for the generation of libraries with random insertions, repeats and/or deletions.

Method	Principle	Mutational scope (Type of mutations/Number per target sequence)	Frameshift InDels (%)	Reference
**RID**	Random Insertion/Deletion mutagenesis. (1) A circular single-stranded DNA (ssDNA) corresponding to the sense chain of the target gene is produced from the linear double-stranded target gene by linker ligation, restriction digestion, circularization by self-ligation and exonuclease digestion to remove the anti-sense chain. (2) Random cleavage (linearization) of the circular ssDNA at single positions by treatment with Ce(IV)‐EDTA complex. (3) Ligation of 5'- and 3'- anchors at both ends of the ssDNA. These anchors are designed differently depending whether a deletion or an insertion is to be introduced. (4) PCR amplification of the DNAs linked to the two anchors at both ends. (5) Digestion by a type IIS restriction enzyme (*e.g.*, BciVI) removes the anchor and leaves a deletion or an insertion in the target gene (depending on how the anchors' sequences have been designed). (6) Reconstitution of the target gene by self-ligation (re-circularization) and linearization by restriction digestion. The resulting products can then be cloned in a vector to finalize the variant library.	One single InDel per variant; the procedure also generates random point substitutions presumably during the PCR step.**[a]**	~10%	20
**Segmental mutagenesis**	(1) The vector is first linearized either at the 5′ or 3′ end of the target gene. (2) Progressive BAL-31 exonuclease action and removal of the remaining vector DNA yields two batches of either 3′ or 5′ truncated gene fragments. (3) Combinatorial assembly of these two ends to generate variants of the target gene yields the segmental mutagenesis library which is then ligated into a vector and transformed into *E.coli*.	One single deletion or one tandem repeat per variant (on a defined region of the target gene)	~66%	21
**RAISE**	RAndom Insertional-deletional Strand Exchange mutagenesis. (1) The target gene is fragmented using DNAseI. (2) The obtained fragments are extended randomly using Terminal deoxynucleotidyl transferase (TdT). (3) Assembly PCR with the TdT-extended fragment results in the shuffling of InDels and substitutions (generated by TdT or during the PCR steps) within the target gene.	Combination of region-exchanged mutations and substitutions. **[b]**	~66%	22
**COBARDE**	Codon-based random deletion mutagenesis. (1) Chemical synthesis (based on the phosphoramidite method) generating a population of mutagenic oligonucleotides with multiple codon deletions in reference to the target gene. The mutagenic process consists of multiple successive cycles of 3-nucleotide extension as follow: (i) transient blockage of a fraction of the synthesized oligos, (ii) extension of the unblocked oligos by 3 nucleotides, (iii) removal of the blocking groups. (2) The resulting oligonucleotide mixture (corresponding the deletion variants) is purified, duplexed using a DNA polymerase and ligated into a vector.	One or multiple combined codon-based deletions per variant (usually on a defined region of the target gene). **[c]**	<5%	23
**TRINS**	Tandem repeat insertion (TRINS). (1) The target gene is fragmented using DNAseI. (2) An aliquot of the generated fragments is converted into single-stranded circular DNA using CircLigase. (3) Tandem repeats are generated by mixing linear fragments together with circularized fragments in an assembly PCR reaction involving rolling-circle polymerisation. (4) Assembly PCR products are then cloned to finalized the TRINS library.	One or multiple tandem repeats per variants. Tandem repeat size variable (depending on the size of the initial DNAseI linear fragment). **[d]**	~66%	24
**Pentapeptide scanning**	(1) An engineered transposon is randomly inserted within the vector containing the target gene by *in vivo* or *in vitro* reaction (depending on the type of transposon	One single insertion of defined size and sequence (5 nucleotide triplets) per variant.	Not reported	

	used). The sub-library consisting of only of the target gene with a single transposon insertion can be isolated by DNA electrophoresis and size selection. (2) Restriction digestion (*e.g.,* with NotI in the case of modified Mu transposon) leaves a 15 bp insertion after self-ligation of the target gene.			25, 26
**TND**	Triplet nucleotide deletion<br>1) An engineered transposon (dubbed MuDel) is randomly inserted within the vector containing the target gene using *in vitro*. The sub-library consisting of only of the target gene with a single transposon insertion can be isolated by DNA electrophoresis and size selection. (2) Digestion with type IIS restriction enzyme MlyI, results in a triplet deletion upon self-ligation of the target gene.	One single nucleotide triplet deletion per variant. **[e]**	Not reported	1, 27
**CDM**	Codon Deletion Mutagenesis<br>1) The target gene is cloned in a vector such as the resulting protein is N-terminally fused to an intein. (2) An engineered transposon (dubbed MuCDM) is then randomly inserted within the target gene using *in vitro*. MuCDM contains an intein sequence fused to an antibiotic resistance (*e.g.*, TEM1), thus enabling selection only if transposon insertion is in the reading frame of the target gene. (3) An inverse PCR reaction with primers based on the transposon's terminal sequences is performed to amplify the vector from the transposon's insertion point. These primers carry a carefully positioned type IIS restrictions site (*e.g.*, for BsgI) to remove a specific number of nucleotides from the resulting inverse PCR product. (3) Digestion by the type IIS restriction enzyme removes 1 to 5 nucleotide codons from the target gene depending on the positioning of the recognition sequence on the primers. (4) CDM libraries are generated upon self-ligation and transformation of the vector carrying the target gene variants in *E. coli*.	Deletions of one to five consecutive codons. **[f]**	<10%	28
**Extensive gene truncation**	1) An engineered transposon (MuDel) is randomly inserted within the target gene by *in vitro* transposition. (2) 5′ and 3′ fragment sub-libraries of the target gene are amplified in two separate PCR reactions. In each reaction, one primer is complementary to the 5′ or 3′ constant regions of the target gene (adding BsaI at these ends), and the other to a sequence located in the transposon. (3) Digestion with BsaI creates unique overhangs in each sub-library complementary to unique overhangs in a DNA linker (free of MlyI sites) to favor directional ligation between these sub-libraries and the linker (4) The product of ligation was digested with MlyI removing the transposon sequence. (5) Intramolecular blunt-end ligation results in a circular product joining the 5′ and 3′ terminal fragments of the target gene. This circularized product is a library corresponding to the target genes with extensive truncation. (7) PCR on this circular library with primers complementary to the termini of the target gene results in a linear version of the extensive truncation library. (8) The final library of truncated variants of the desired size range is isolated by gel electrophoresis and cloned in a vector.	Extensive DNA truncations of desired size range. **[g]**	Not reported	29
**InDel assembly**	Assembly approach relying on successive cycles of DNA restriction and ligation to assemble a DNA library on beads. At each assembly cycle, DNA templates immobilized on beads are restricted with a type IIs endonuclease (*e.g.*, SapI) and building blocks annealed and ligated. After ligation, the cycle can be restarted. Compositional variation is achieved primarily by combining controlled pools of building blocks of various length.	One or multiple combined codon-based insertions and deletions per variant (usually on a defined region of the target gene). **[h]**	Not reported	30

(Table S16 continued)

[a] The RID mutagenesis was validated by randomly replacing three consecutive bases by recognition sequence for BglII (AGATCT) in the GFPuv gene: 17 variants (out of 19 randomly picked variants for sequencing; ~90%) displayed the desired mutations; 2 variants out of the pool of 19 variants (~10%) were frameshifted (deletion of 4 consecutive bases instead of 3); in addition, 6 variants out of 19 (~30%) also displayed single point substitution. The RID mutagenesis has also been applied to randomly replace three consecutive bases by a mixture of 20 codons, effectively resulting in point substitution mutants.

[b] RAISE was validated using TEM-1 beta-lactamase as target gene. After transformation of the library (~2,000 variants), 41 colonies were randomly picked and sequenced leading to the identification of region-exchanged mutations and point substitutions. Twenty-nine region-exchanged mutations were found in 19 variants. The number of the region-exchanged mutations per variant was 1 (12 variants), 2 (6 variants), or 5 (1 variants). Approximately two-thirds of the region-exchanged mutations were frameshifts. Seventy-nine point substitutions were identified over 34 variants, among which 15 had also region-exchanged mutations. Three variants out of the 41 that sequenced were parental sequences, presumably due to vector self-ligation.

[c] COBARDE was validated using a sequence of 9 residues forming the omega loop in TEM-1 beta-lactamase: 4 parental sequences (presumably vector self-ligation) and 1 frame-shift (insertion of 1 bp) were found out of 34 sequenced transformants.

[d] TRINS was validated using three different templates: TEM-1 beta-lactamase, m.HaeIII methyltransferase and KE70 R6 (a laboratory-evolved variant of computationally designed Kemp eliminase). Out of 35 sequenced variants (from the three naïve libraries), 27 carried one insertion per gene, 4 had two and 4 had three. Two-third of the tandem repeats (23 of 35) resulted in frameshift. The sequenced variants also carried ~2 random point substitutions per variant presumably incorporated during PCR steps.

[e] TND was validated using TEM-1 beta-lactamase (Jones, 2005) and eGFP (Baldwin et al., 2009; Arpino et al., 2014) as templates. In the case of TEM-1, the library generation process was combined with two consecutive selection steps: (i) selection for loss of ampicillin resistance upon transposition insertion within *bla*) and (ii) selection for retention of antibiotic resistance upon triplet nucleotide deletion. In the case of eGFP, the final library consisted of ~2,500 variants and 153 variants were chosen for sequencing based on the colony phenotype (88 fluorescent and 65 nonfluorescent). This led to the identification of 87 unique triplet deletions (out of 153): 42 triplet deletions among the 88 fluorescent variants and 45 among the 65 nonfluorescent ones. No additional point substitutions or frameshifts were observed among the sequenced variants.

[f] CMD was validated using super folder GFP (sfGFP) as template. Five libraries, corresponding to the deletion of 1 to 5 consecutive codons, were generated and around 20 variants from each library (amounting to a total of 104 sequences) were sequenced, showing that the majority of the variants (~92%) contained the desired deletions. Eight out of 104 sequences had either no mutations or unwanted mutations, most of them due to incomplete BsgI digestion.

[g] The extensive gene truncation method was validated using an artificial RNA ligase enzyme (DNA size ~ 350 bp) as template and resulted in a library with truncations up to ~235 bp. Next generation sequencing analysis of the library revealed that it contained 9,006 unique deletions (~32% of the 27,730 possible unique deletions in this size range). The distribution of deletion lengths was found to range between 6 and 235 nucleotides in length. Deletions longer than 110 bp were observed at 50% or greater of the number of all possible deletions. The library was subjected to in vitro selection and functional variants with deletions of up to 18 amino acids of the parental enzyme.

[h] InDel assembly was validated using part of TEM-1 beta-lactamase's omega loop (5 residues, $_{164}$RWEPE$_{168}$). The library was designed in order to explore the sequence neighbourhood of a previously reported variant ($_{164}$RYYGE$_{168}$) by using biased mixes of building blocks. The resulting library was analysed by next-generation sequencing before and after selection for ceftazidime resistance and demonstrated selective enrichment of the target sequence ($_{164}$RYYGE$_{168}$) as well as variants with extensions (*e.g.*, $_{164}$RGYMKER$_{168b}$).

Supplementary Table S17. Oligonucleotides used in this study.

Experiment	Oligonucleotide name and sequence
Preparation of SubsNNN by PCR using pUC57-Del2 as template	**Subs-F:** 5'-[Phos]-ATGTCGACTCGACTAGTGCTTGGATTCTCA-3'<br>**Subs-B:** 5'-[Phos]-NNNGGGATGACTCCATGGACTTCGC-3'<br>(MlyI sites underlined)
TransIns adapter to generate pUC57-TransIns from pUC57-TransDel	**TransIns-F:** 5'-[Phos]-AATTCAGATCTGCGGCCGCGCACGAAAAACGCGAAAGCGTTTCACGAT-AAATGCGAAAACGGA -3'<br>**TransIns-R:** 5'-[Phos]-CTAGTCCGTTTTCGCATTTATCGTGAAACGCTTTCGCGTTTTTCGTGCG-CGGCCGCAGATCTG-3'<br>(NotI sites underlined)
Del3 adapter to generate pUC57-Del3 from pUC57-Del2	**Del3-F:** 5'-[Phos]-CATGGAGTCATCCCGGGA-3'<br>**Del3-R:** 5'-[Phos]-AGCTTCCCGGGATGACTC-3'
Ins adapter to generate pUC57-Ins from pUC57-Del2	**Ins-F:** 5'-[Phos]-AATTCTAGATCTGCGGCCGCATCCGTCTTCAGTCGCTGCTGA-3'<br>**Ins-R:** 5'-[Phos]-CTAGTCAGCAGCGACTGAAGACGGATGCGGCCGCAGATCTAG-3'
Ins1/2/3 adapter to generate libraries pUC57-Ins1/2/3 from pUC57-Ins	**Ins1/2/3-F:** 5'-[Phos]-CATGGCTGAAGGCCACTCGAGATCGAT (NNN)$_{1/2/3}$ GCGCTGACTCA-3'<br>**Ins1/2/3-R:** 5'-[Phos]-AGCTTGAGTCAGCGC (NNN)$_{1/2/3}$ ATCGATCTCGAGTGGCCTTCAGC-3'
Removal of MlyI recognition site in the origin of replication of pUC19 by saturation mutagenesis	**Ori-MlyI-F:** 5'-ACCCGGTAAGACACGACTTATCGCCACTGGCA-3'<br>**Ori-MlyI-B:** 5'-GTGTCTTACCGGGTTGGNNTCAAGACGATAGTTACCGGA-3'
Removal of AcuI recognition site in the origin of replication of pUC19 by saturation mutagenesis	**Ori-AcuI-F:** 5'-TATCTGCGCTCTGNNGAAGCCAGTTACCTT-3'<br>**Ori-AcuI-B:** 5'-AGCGCAGATACCAAATACTGTTCTTCTAGTGTAGCCGTA-3'
Amplification of the origin of replication of pUC19 for assembly into the pID vectors	**Ori-AflII:** 5'-GGACTTAAGGAGCAAAAGGCCAGCAAAAGG-3'<br>**Ori-SpeI:** 5'-GCACACTAGTCTCATGACCAAAATCCCTTAACG-3'
(1) Amplification of TetR from pASK-IBA5plus (TetR-F/TetR-B)<br>(2) Amplification of AmpR from pID-T7 (mTEM1-F/mTEM1-B)<br>(3) Overlap PCR to form AmpR-TetR operon (mTEM1-F/TetR-B)	**TetR-F:** 5'-TGATTAAGCATTGGTAGGAATTAATGATGTCTCGTT-3'<br>**TetR-B:** 5'-TTAAACTAGTGAAGTTACCATCACGGA-3'<br>**mTEM1-F:** 5'-CTGAAAGGACGTCAGGTGGCAC-3'<br>**mTEM1-B:** 5'-TAATTCCTACCAATGCTTAATCAGTGAGGCA-3'
Amplification of Tet promoter from pASK-IBA5plus	**Tet-prom-F:** 5'-AGGCTTAAGACATGACCCGACACCATCGA-3'<br>**Tet-prom-B:** 5'-GGCTCATATGTATATCTCCTTCTTAAAG-3'

SUPPLEMENTARY REFERENCES

1. Jones DD. Triplet nucleotide removal at random positions in a target gene: the tolerance of TEM-1 beta-lactamase to an amino acid deletion. *Nucleic Acids Res* **33**, e80 (2005).

2. DePristo MA, Weinreich DM, Hartl DL. Missense meanderings in sequence space: a biophysical view of protein evolution. *Nat Rev Genet* **6**, 678-687 (2005).

3. Mayer S, Rudiger S, Ang HC, Joerger AC, Fersht AR. Correlation of levels of folded recombinant p53 in escherichia coli with thermodynamic stability in vitro. *J Mol Biol* **372**, 268-276 (2007).

4. Wyganowski KT, Kaltenbach M, Tokuriki N. GroEL/ES buffering and compensatory mutations promote protein evolution by stabilizing folding intermediates. *J Mol Biol* **425**, 3403-3414 (2013).

5. Lu Q. Seamless cloning and gene fusion. *Trends Biotechnol* **23**, 199-207 (2005).

6. Afriat-Jurnou L, Jackson CJ, Tawfik DS. Reconstructing a missing link in the evolution of a recently diverged phosphotriesterase by active-site loop remodeling. *Biochemistry* **51**, 6047-6055 (2012).

7. Hoque MA*, et al.* Stepwise Loop Insertion Strategy for Active Site Remodeling to Generate Novel Enzyme Functions. *ACS Chem Biol* **12**, 1188-1193 (2017).

8. Kaltenbach M, Emond S, Hollfelder F, Tokuriki N. Functional Trade-Offs in Promiscuous Enzymes Cannot Be Explained by Intrinsic Mutational Robustness of the Native Activity. *PLoS Genet* **12**, e1006305 (2016).

9. Ikeda RA, Ligman CM, Warshamana S. T7 promoter contacts essential for promoter activity in vivo. *Nucleic Acids Res* **20**, 2517-2524 (1992).

10. Zhang J, Kobert K, Flouri T, Stamatakis A. PEAR: a fast and accurate Illumina Paired-End reAd mergeR. *Bioinformatics* **30**, 614-620 (2014).

11. Langmead B, Salzberg SL. Fast gapped-read alignment with Bowtie 2. *Nat Methods* **9**, 357-359 (2012).

12. Li H*, et al.* The Sequence Alignment/Map format and SAMtools. *Bioinformatics* **25**, 2078-2079 (2009).

13. Rice P, Longden I, Bleasby A. EMBOSS: the European Molecular Biology Open Software Suite. *Trends Genet* **16**, 276-277 (2000).

14. Tokuriki N, Jackson CJ, Afriat-Jurnou L, Wyganowski KT, Tang R, Tawfik DS. Diminishing returns and tradeoffs constrain the laboratory optimization of an enzyme. *Nat Commun* **3**, 1257 (2012).

15. Campbell E*, et al.* The role of protein dynamics in the evolution of new enzyme function. *Nat Chem Biol* **12**, 944-950 (2016).

16. Kaltenbach M, Jackson CJ, Campbell EC, Hollfelder F, Tokuriki N. Reverse evolution leads to genotypic incompatibility despite functional and active site convergence. *Elife* **4**, (2015).

17. Krissinel E, Henrick K. Inference of macromolecular assemblies from crystalline state. *J Mol Biol* **372**, 774-797 (2007).

18. Miller S, Janin J, Lesk AM, Chothia C. Interior and surface of monomeric proteins. *J Mol Biol* **196**, 641-656 (1987).

19. Tokuriki N, Stricher F, Schymkowitz J, Serrano L, Tawfik DS. The stability effects of protein mutations appear to be universally distributed. *J Mol Biol* **369**, 1318-1332 (2007).

20. Murakami H, Hohsaka T, Sisido M. Random insertion and deletion of arbitrary number of bases for codon-based random mutation of DNAs. *Nat Biotechnol* **20**, 76-81 (2002).

21. Pikkemaat MG, Janssen DB. Generating segmental mutations in haloalkane dehalogenase: a novel part in the directed evolution toolbox. *Nucleic Acids Res* **30**, E35-35 (2002).

22. Fujii R, Kitaoka M, Hayashi K. Random insertional-deletional strand exchange mutagenesis (RAISE): a simple method for generating random insertion and deletion mutations. *Methods Mol Biol* **1179**, 151-158 (2014).

23. Osuna J, Yanez J, Soberon X, Gaytan P. Protein evolution by codon-based random deletions. *Nucleic Acids Res* **32**, e136 (2004).

24. Kipnis Y, Dellus-Gur E, Tawfik DS. TRINS: a method for gene modification by randomized tandem repeat insertions. *Protein Eng Des Sel* **25**, 437-444 (2012).

25. Hallet B, Sherratt DJ, Hayes F. Pentapeptide scanning mutagenesis: random insertion of a variable five amino acid cassette in a target protein. *Nucleic Acids Res* **25**, 1866-1867 (1997).

26. Hayes F, Hallet B. Pentapeptide scanning mutagenesis: encouraging old proteins to execute unusual tricks. *Trends Microbiol* **8**, 571-577 (2000).

27. Arpino JA, Reddington SC, Halliwell LM, Rizkallah PJ, Jones DD. Random single amino acid deletion sampling unveils structural tolerance and the benefits of helical registry shift on GFP folding and structure. *Structure* **22**, 889-898 (2014).

28. Liu SS, Wei X, Ji Q, Xin X, Jiang B, Liu J. A facile and efficient transposon mutagenesis method for generation of multi-codon deletions in protein sequences. *J Biotechnol* **227**, 27-34 (2016).

29. Morelli A, Cabezas Y, Mills LJ, Seelig B. Extensive libraries of gene truncation variants generated by in vitro transposition. *Nucleic Acids Res* **45**, e78 (2017).

30. Tizei PAG, Harris E, Renders M, Pinheiro VB. InDel assembly: A novel framework for engineering protein loops through length and compositional variation (2017), bioarxiv, doi.org/10.1101/127829.
